# Synthesis and Characterization of Poly(hydrogen halide) Halogenates (–I)

**DOI:** 10.1002/chem.202001864

**Published:** 2020-09-16

**Authors:** Patrick Voßnacker, Simon Steinhauer, Julia Bader, Sebastian Riedel

**Affiliations:** ^1^ Fachbereich Biologie, Chemie, Pharmazie Institut für Chemie und Biochemie–Anorganische Chemie Freie Universität Berlin Fabeckstrasse 34/36 14195 Berlin Germany

**Keywords:** acidity, hydrogen bonds, hydrogen halide, quantum-chemical calculations, X-ray structures

## Abstract

Herein, we report the synthesis and characterization of a variety of novel poly(hydrogen halide) halogenates (−I). The bifluoride ion, which is known to have the highest hydrogen bond energy of ≈160 kJ mol^−1^, is the most famous among many examples of [X(HX)_*n*_]^−^ anions (X=F, Cl) known in the literature. In contrast, little is known about poly(hydrogen halide) halogenates containing two different halogens, ([X(HY)_*n*_]^−^). In this work we present the synthesis of anions of the type [X(HY)_*n*_]^−^ (X=Br, I, ClO_4_; Y=Cl, Br, CN) stabilized by the [PPh_4_]^+^ and [PPN]^+^ cation. The obtained compounds have been characterized by single‐crystal X‐ray diffraction, Raman spectroscopy and quantum‐chemical calculations. In addition, the behavior of halide ions in hydrogen fluoride was investigated by using experimental and quantum‐chemical methods in order to gain knowledge on the acidity of hydrogen halides in HF.

## Graphical abstract

**An alternative anion**: Poly(hydrogen halide) halogenates have been shown to be a structurally diverse class of compounds with many applications in various fields of chemistry. The synthesis and characterization of various novel anions of the type [X(HY)_*n*_]^−^ (X=Br, I, ClO_4_; Y=Cl, Br, CN, X≠Y) is presented in this work. Additionally, the acidity of hydrogen halides in anhydrous HF was investigated.

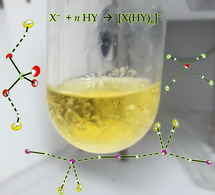

## Introduction

Polyhalides ([X(X_2_)_*n*_]^−^), which are prominent examples for halogen‐bonded systems, show a large structural diversity as well as a variety of possible applications.^1^ A related class of compounds, the poly(hydrogen halide) halogenates (−I) ([X(HY)_*n*_]^−^), consists of a central halide (X^−^) that is coordinated to hydrogen halide molecules (HY). This combination of halide anions with hydrogen halides, which has a large positive charge on the hydrogen atom, yields compounds with quite strong hydrogen‐bonding interactions. The most prominent example for this class of compounds, the bifluoride ion [FHF]^−^, has the highest known hydrogen bond energy of 160 kJ mol^−1^_._ 2 To gain knowledge on strongly hydrogen‐bonded systems, the synthesis of novel compounds containing [X(HY)_*n*_]^−^ anions, while X and Y are halogens, is of interest.

**Scheme 1:**
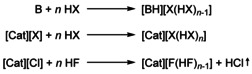
Synthetic routes for the synthesis of poly(hydrogen halide) halogenates (−I).

Three different synthetic routes have been used to synthesize a variety of different [X(HX)_*n*_]^−^ anions (Scheme 1).^3^, ^4^, ^5^, ^6^, ^7^, ^8^, ^9^

Mootz′ group prepared and characterized salts containing [X(HX)_*n*_]^−^ anions (X=F (*n=*1–6), Cl (*n=*2–5)). Crystal structures of [Cat][F(HF)_*n*_] ([Cat]=[NO]^+^ (*n=*3, 4),^8^ [NMe_3_H]^+^ (*n=*2–6),^6^ [NMe_4_]^+^ (*n=*2, 3, 5)^3^) as well as [Cat][Cl(HCl)_*n*_] (Cat=[SMe_2_H]^+^ (*n=*3, 4),^7^ [C_5_H_5_NH]^+^ (*n=*1, 4, 5)^5^) were determined by X‐ray diffraction (XRD) using a miniature zone‐melting technique for crystallization. Structures containing a protonated base as a cation, like [NMe_3_H]^+^, show strong hydrogen bonding between anion and cation, whereas less coordinating cations, for example, [NMe_4_]^+^, yield more isolated anions. For the heavier homologues only [BrHBr]^−[10]^ and [Br(HBr)_2_]^−[11]^ have been characterized by X‐ray diffraction while no molecular structure in the solid state is known for [I(HI)_*n*_]^−^ anions. The behavior of [X(HX)_*n*_]^−^ anions in solution was investigated using NMR spectroscopy by several groups. The group of Limbach performed ^1^H and ^19^F NMR experiments on a 1:2 mixture of [NBu_4_]F and HF in the temperature range between 110 and 150 K and was able to identify [F(HF)_*n*_]^−^ (*n*=1–4) which coexist in solution.^12^ Campbell and Johnson conducted ^1^H NMR experiments on the [Im][Cl(HCl)_*n*_] ([Im]^+^=1‐ethyl‐3‐methyl‐1*H*‐imidazolium) system at room temperature, and only observed one signal, which shifts to a higher field when the concentration of HCl is increased. They explained this finding with a fast equilibrium between different [Cl(HCl)_*n*_]^−^ species.^13^ Further studies on the ternary HCl:[Im]Cl:AlCl_3_ systems have shown that various species of the type [X(HCl)]^−^ are present in solution (X=Cl^−^, [ClHCl]^−^, [Cl(HCl)_2_]^−^, [AlCl_4_]^−^, [Al_2_Cl_7_]^−^. By changing the ratios between the components solutions with various acidities from weakly acidic solutions (high concentration of [Im]Cl; [ClHCl]^−^ as dominant acidic species) to super‐acidic solutions (high concentrations of AlCl_3_; [Al_2_Cl_7_]^−^⋅HCl as dominant acidic species) can be obtained.^14^ Electrochemical studies suggests that upon dissolution of HCl in chloride free ionic liquids also [ClHCl]^−^ is formed.^15^ Enthalpies, entropies and free energies for the formation of poly(hydrogen halide) halogenates (−I) ([X(HY)_*n*−1_]^−^+HY→[X(HY)_*n*_]^−^) have been determined using high pressure mass spectrometry. In general, the free energies of formation are highest for the combination of the most basic hydrogen bond acceptor (F^−^) with the best hydrogen bond donor (HF). Vice versa, the weakest hydrogen bond is formed by the least basic hydrogen bond acceptor (I^−^) with the worst hydrogen bond donor (HI). This tendency is observed for all possible combinations of X and Y.^16^, ^17^

Poly(hydrogen halide) halogenates (−I), especially [F(HF)_*n*_]^−^, show some useful properties which lead to a variety of applications. K[F(HF)_2_] is electrolyzed in the synthesis of elemental fluorine to avoid the low conductivity of anhydrous HF (aHF).^18^ In addition [Cat][F(HF)_*n*_] (Cat=for example, [NEt_3_H]^+^,^19^, ^20^ [C_5_H_5_NH]^+^,^21^, ^22^, ^23^ [EMIM]^+^ (1‐ethyl‐3‐methylimidazolium)^9^, ^11^) have been used as a reagent in organic chemistry which are safer and more convenient to handle when compared to highly toxic anhydrous HF with its boiling point at 19.5 °C. They can be used to perform hydro‐ and halofluorination reactions as well as epoxide ring openings and deprotection of silyl ethers.^9^, ^19^, ^20^, ^21^, ^22^, ^23^ Recently Sharpless et al. proposed the sulfur(VI) fluoride exchange (SuFEx) as a new kind of “click chemistry”.^24^ Since then, the reaction has been used in the synthesis of pharmaceutically important triflones and bis(trifluoromethyl)sulfur oxyimines^25^ as well as in the synthesis of polysulfates and sulfonates.^26^ For many SuFEx reactions bifluoride anions as well as higher poly(hydrogen fluoride) fluorates (−I) have been tested as catalysts and it was shown that they have a significantly higher activity compared to previously used organosuperbases.^26^

Even though poly(hydrogen halide) halogenates have been studied in detail over the last decades little is known about the [X(HY)_*n*_]^−^ anion. In addition the known [Cl(HCl)_*n*_]^−^ (*n*>2) compounds exhibit low melting points and in general a low stability. Therefore, they were only characterized by X‐ray diffraction. Herein we present the synthesis of more stable [Cat][Cl(HCl)_4_] salts (Cat=[PPN]^+^ (bis(triphenylphosphoranylidene)iminium), [PPh_4_]^+^, [AsPh_4_]^+^) which allow further investigation by low temperature Raman spectroscopy. In addition a variety of [Cat][X(HY)_*n*_] ([Cat]=[PPh_4_]^+^, [PPN]^+^; X=Cl, Br, I, ClO_4_; Y=F, Cl, Br, CN) compounds were synthesized and characterized by X‐ray diffraction and Raman spectroscopy.

## Results and Discussion

### Tetraphenylphosphonium salts [PPh_4_][X(HCl)_4_]

**Scheme 2:**
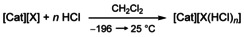
Synthesis of poly(hydrogen chloride) halogenates (−I) ([Cat]=[PPN]^+^, [PPh_4_]^+^; X=Cl, Br, I).

Poly(hydrogen chloride) halogenates (−I) were prepared by condensing stoichiometric amounts of hydrogen chloride onto a dichloromethane solution of the respective halide salts (Scheme 2).

By slowly cooling saturated solutions of the reaction mixture to −40 °C single crystals of the respective poly(hydrogen chloride) halogenate (−I) salts were obtained. The obtained crystals are stable at −40 °C under a hydrogen chloride atmosphere but show a noticeable loss of hydrogen chloride when handled under a nitrogen stream of −40 °C.

**Figure 1:**
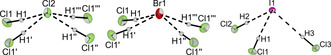
Molecular structure of the anions in [PPh_4_][Cl(HCl)_4_], [PPh_4_][Br(HCl)_4_] and [PPh_4_][I(HCl)_3_] in the solid state with thermal ellipsoids shown at 50 % probability. Cations and disorders have been omitted for clarity (see Figures S1–S3 for representations including cations and disorders). Selected interatomic distances [pm]: Cl1‐Cl2 340.1(1), Cl1‐Cl2‐Cl1′′ 144.3(1), Cl1‐Br1 353.4(1), Cl1‐Br1‐Cl1′′ 145.1(1), Cl1‐I1 374.1(1), Cl2‐I1 374.1(1), Cl3‐I1 377.2(1), Cl1‐I1‐Cl3 70.7(1), Cl1‐I1‐Cl2 89.1(1), Cl2‐I1‐Cl3 113.0(1).

Using [PPh_4_]X (X=Cl, Br, I) as a starting material single crystals of [PPh_4_][Cl(HCl)_4_], [PPh_4_][Br(HCl)_4_] and [PPh_4_][I(HCl)_3_] were obtained by using the method described above. [PPh_4_][Cl(HCl)_4_] and [PPh_4_][Br(HCl)_4_] are isostructural and crystallize in the tetragonal space group *P*4/*n* while [PPh_4_][I(HCl)_3_] crystallizes in the monoclinic space group *P*2_1_/*c* (Figure 1)

Since the position of hydrogen atoms cannot be determined accurately by XRD only halogen–halogen distances will be discussed. [Cl(HCl)_4_]^−^ as well as [Br(HCl)_4_]^−^ show a square pyramidal structure with *R*(Cl−Cl)=340.1(1) pm and *R*(Br−Cl)=353.4(1) pm. Those values are in good agreement with the halogen–halogen distances from quantum‐chemical calculations in the gas phase on the B3LYP(D3BJ)/def2‐TZVPP (SCS‐MP2/def2‐TZVPP) level of theory (*R*(Cl−Cl)=341.1 pm (342.0 pm) and *R*(Cl−Br)=358.1 pm (358.5 pm)). The observed increase of the halogen–halogen distance going from [Cl(HCl)_4_]^−^ to [Br(HCl)_4_]^−^ also correlates well with the increase in ion radii when going from chlorine to bromine (Δ*R*(X−Cl)=13.3 pm, Δ*R*_Ion_(Br, Cl)=15 pm^27^).

When [PPh_4_]I is used as starting material the reaction with four equivalents of HCl yields [PPh_4_][I(HCl)_3_]. [PPh_4_][I(HCl)_4_] could not be obtained with this cation even when higher amounts of HCl were used. Quantum‐chemical calculations on the B3LYP(D3BJ)/def2‐TZVPP (SCS‐MP2/def2‐TZVPP) level of theory show that the addition of the fourth HCl molecule to [I(HCl)_3_]^−^ is only by −8.8 (−4.5) kJ mol^−1^ exergonic in the gas phase. In comparison the formation of [Cl(HCl)_4_]^−^ (−13.6 (−8.8) kJ mol^−1^) and [Br(HCl)_4_]^−^ (−11.6 (−8.4) kJ mol^−1^) is more favored. Free reaction energies were calculated for the formation of the most favorable geometry which is a tetrahedral structure for all [X(HCl)_4_]^−^ anions (Figure 4). These calculations indicate a lower stability of the [I(HCl)_4_]^−^ anion which might explain why it could not be isolated as a [PPh_4_]^+^ salt. The [I(HCl)_3_]^−^ anion shows a distorted trigonal pyramidal structure with *R*(I−Cl)=374.1(1)–377.2(1) pm that are in good agreement with the values obtained from quantum‐chemical calculations of *R*(I−Cl)=375.8 pm (377.9 pm).

**Figure 2:**
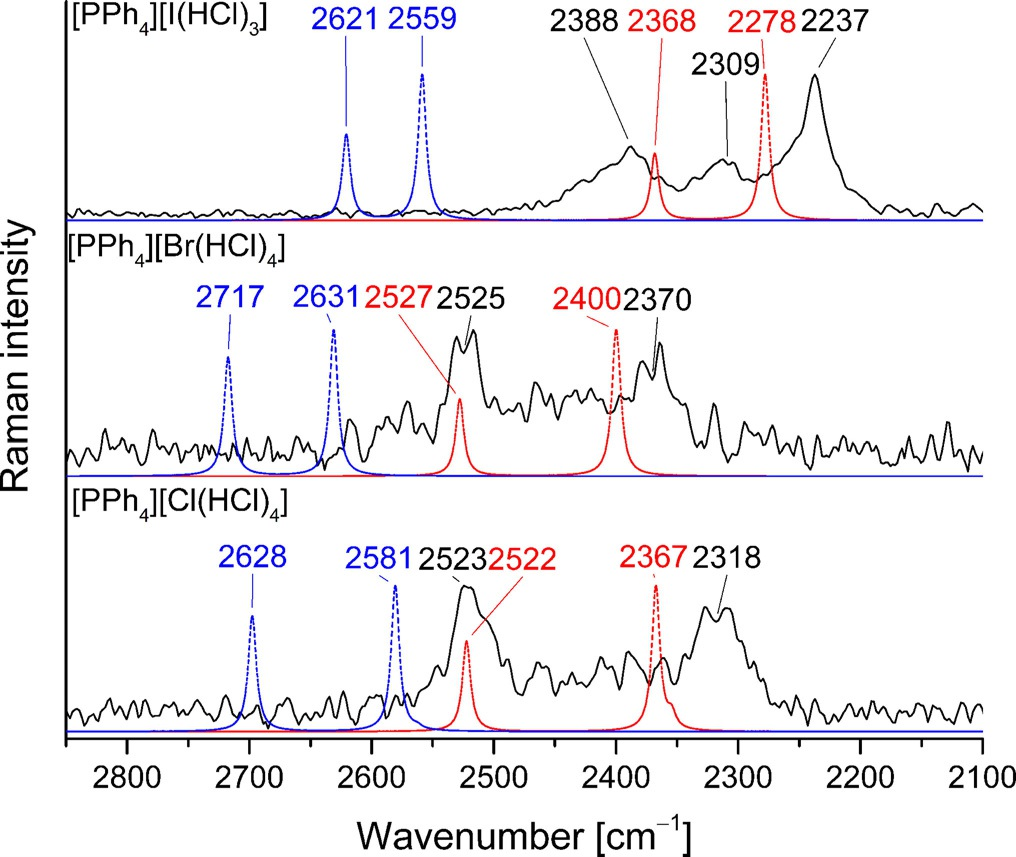
Raman spectra of single crystals of [PPh_4_][X(HCl)_*n*_] recorded at −196 °C (black) as well as calculated gas phase spectra (B3LYP/def2‐TZVPP (red) and MP2/def2‐TZVPP (blue)) of [X(HCl)_*n*_]^−^ (*n=*4 for X=Cl, Br and *n=*3 for X=I).

The most important contributions to the binding energy for strong hydrogen bonding are charge transfer, electrostatic and Pauli repulsion. For hydrogen bonding between halide ions and hydrogen halides the charge transfer contribution can be described as a donation of electron density from the lone pair of the halide ion into the σ* orbital of the HX bond. The partial occupation of the σ* orbital leads to a weakening of the HX bond. This results in an increase of the H−X distance as well as a shift of the H−X stretching frequency to lower wavenumbers which can be observed in the Raman spectrum.^28^ The experimental Raman spectra recorded at −196 °C as well as the calculated spectra (B3LYP/def2‐TZVPP and MP2/def2‐TZVPP) of [PPh_4_][Cl(HCl)_4_], [PPh_4_][Br(HCl)_4_] and [PPh_4_][I(HCl)_3_] are shown in Figure 2. For the *C*_4*v*_ symmetric [Cl(HCl)_4_]^−^ and [Br(HCl)_4_]^−^ anions three H−Cl stretching modes are expected (A_1_, B_2_, E). In the experimental spectra, two bands are observed. The bands at 2517 cm^−1^ ([Br(HCl)_4_]^−^) and 2523 cm^−1^ ([Cl(HCl)_4_]^−^) can be assigned to the symmetric stretching mode of the HCl molecules (A_1_) while the bands at 2370 cm^−1^ ([Br(HCl)_4_]^−^) and 2318 cm^−1^ ([Cl(HCl)_4_]^−^) can be assigned to one antisymmetric H−Cl stretching mode (*B*_1_). The second antisymmetric stretching mode (*E*) is calculated to have a significantly lower intensity and to be in close proximity to the B_1_ symmetric stretching mode. This might explain why only one band is visible for the antisymmetric stretching modes. In general the calculated spectra on the B3LYP(D3BJ) level of theory agree well with the experimental ones even though the calculation predicts the symmetric and asymmetric stretching bands to be closer together. As known for calculations at the MP2 level of theory, the HCl bond strength is overestimated and therefore the calculated wavenumbers for the HCl stretching vibration are significantly too high.

For [PPh_4_][I(HCl)_3_] three bands at 2390, 2312, 2237 cm^−1^ are observed in the H‐Cl stretching region. This can be explained by a reduced symmetry of the [I(HCl)_3_]^−^ anion within the crystal when compared to the *C*_3*v*_ symmetric optimized gas‐phase structure. Therefore the degeneracy of the asymmetric stretching mode (*E*) is removed and the three crystallographically independent HCl molecules in the crystal and contribute each, one band in the vibrational spectrum. The experimental spectra again agree well with the calculated spectra on the B3LYP level of theory.

All observed bands are significantly shifted to lower wavenumbers compared to crystalline HCl at 2705 and 2748 cm^−1^ (measured at −196 °C)^29^ which indicates hydrogen bond interactions. Three major trends can be emphasized in the recorded Raman spectra:

1. The splitting between the symmetric and asymmetric stretching modes is larger for [Cl(HCl)_4_]^−^ compared to [Br(HCl)_4_]^−^. This tendency is observed in the experiment as well as in the calculations.
2. The average wavenumber for the H−Cl stretching is lower for [Cl(HCl)_4_]^−^ compared to [Br(HCl)_4_]^−^. This is expected since chloride is the stronger base and can donate more electron density into the LUMO of the H−Cl bond. Quantum‐chemical calculations (B3LYP(D3BJ)/def2‐TZVPP (SCS‐MP2/def2‐TZVPP)) show a decrease of the H−Cl bond length from 132.3 pm (130.3 pm) for [Cl(HCl)_4_]^−^ to 132.0 pm (130.0 pm) for [Br(HCl)_4_]^−^ which matches the observed shift of the H−Cl stretching vibration.
3. For [I(HCl)_3_]^−^ the shift towards lower wavenumbers is more pronounced compared to the four times coordinated [Cl(HCl)_4_]^−^ and [Br(HCl)_4_]^−^. This indicates that the coordination number has a stronger influence on the weakening of the H−Cl bond than the change of the central halide. Quantum‐chemical calculations (B3LYP(D3BJ)/def2‐TZVPP (SCS‐MP2/def2‐TZVPP)) predict a shortening of the H−Cl bond by 2.1 pm (1.6 pm; X=Cl), 1.7 pm (1.2 pm; X=Br) and 1.3 pm (0.9 pm; X=I) going from [X(HCl)_4_]^−^ to [X(HCl)_3_]^−^ (X=Cl, Br, I). In contrast the difference in the H−Cl bond length is calculated to be 0.3 pm (0.3 pm) between [Cl(HCl)_4_]^−^ and [Br(HCl)_4_]^−^ and 0.2 pm (0.4 pm) between [Br(HCl)_4_]^−^ and [I(HCl)_4_]^−^. These calculations support the experimental results.

### Bis(triphenylphosphoranylidene)iminium salts [PPN][X(HCl)_4_]

**Figure 3:**
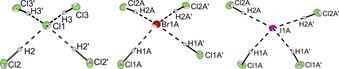
Molecular structure of [PPN][Cl(HCl)_4_], [PPN][Br(HCl)_4_], and [PPN][I(HCl)_4_] in the solid state with thermal ellipsoids shown at 50 % probability. Cation and disorders have been omitted for clarity (see Figures S5–S9 for representation including cations and disorders). Selected interatomic distances [pm]: Cl2‐Cl1 334.8(1), Cl3‐Cl1 341.6(1), Cl1A‐Br1A 347.7(1), Cl2A‐Br1A 354.0(2), Cl1A‐I1A 370.4(2), Cl2A‐I1A 374.8(2).

The weakly coordinating [PPN]^+^ cation has been used to stabilize large anions like [Cl_13_]^−^,^30^ [Cl(BrCl)_6_]^−[31]^ and [Br(BrCN)_3_]^−^.^32^ Therefore [PPN]X was used as a starting material to investigate whether higher coordinated poly(hydrogen halide) halogenates (−I) can be stabilized. Single crystals of [PPN][X(HCl)_4_] (X=Cl, Br, I) were obtained using the method described above. The three isostructural salts crystallize in the monoclinic space group *C*2/*c* (Figure 3).

**Figure 4:**
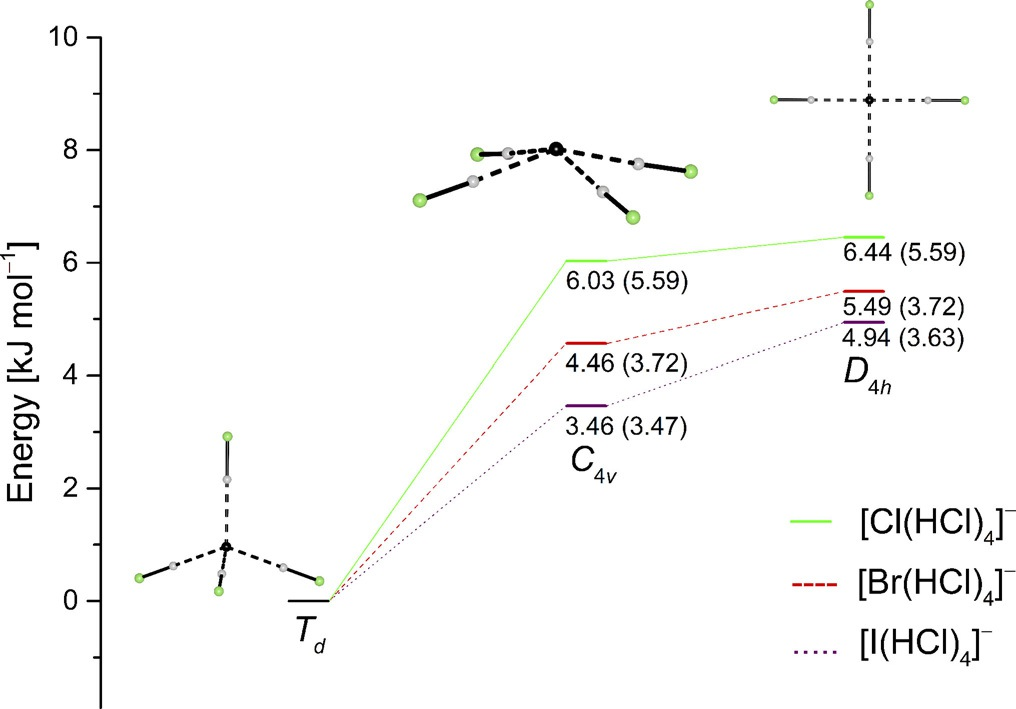
Relative energies of possible geometries for [X(HCl)_4_]^−^ calculated at the B3LYP(D3BJ)/def2‐TZVPP (SCS‐MP2/def2‐TZVPP) level of theory. The pyramidal structures (*C*_4*v*_) are transition states (one imaginary frequency) between tetrahedral structures, while the planar structures (*D*_4*h*_) are saddle points (two imaginary frequencies) at the B3LYP level of theory and either transitions states ([Cl(HCl)_4_]^−^) or minima ([Br(HCl)_4_]^−^, [I(HCl)_4_]^−^) at the MP2 level of theory.

In contrast to the [PPh_4_][X(HCl)_4_] (X=Cl, Br) salts the [X(HCl)_4_]^−^ (X=Cl, Br, I) anions show a distorted tetrahedral geometry when [PPN]^+^ is used as a cation. The halogen–halogen distances of 334.8(1)–341.6(1) pm (X=Cl), 347.7(1)–354.0(2) pm (X=Br) and 370.4(2)–374.8(2) (X=I) are comparable with those obtained for [PPh_4_][X(HCl)_4_] (X=Cl, Br) and are again in good agreement with the calculated distances. Quantum‐chemical calculations show that the tetrahedral structure is the global minimum in the gas phase while the *C*_4*v*_ symmetric pyramidal structure is only 3.46 kJ mol^−1^ (for X=I) to 6.03 kJ mol^−1^ (for X=Cl) higher in energy (Figure 4).

The Raman spectra of the single crystals of [PPN][I(HCl)_4_] show three bands in the HCl stretching region (2472, 2415, 2301 cm^−1^, Figure S17). The bands at 2472 and 2415 cm^−1^ are in good agreement with the calculated bands at the B3LYP(D3BJ)/def2‐TZVPP level of theory for the [I(HCl)_4_]^−^ anion (Table S33). Within the crystal the position of the [I(HCl)_4_]^−^ anion is sometimes occupied by a [I(HCl)_3_]^−^ anion due to a disorder (ratio [I(HCl)_4_]^−^/[I(HCl)_3_]^−^=78:22; Figure S9). This explains a third broad band at 2301 cm^−1^_._ For [PPN][Cl(HCl)_4_] and [PPN][Br(HCl)_4_] no bands are observed in the HCl stretching region which might be due to the low intensity of these bands (Figure S17).

### Halides in aHF

**Scheme 3:**
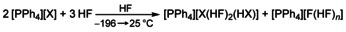
Synthesis of poly(hydrogen fluoride) halogenates (−I) (X=Cl, Br, I).

Even though poly(hydrogen fluoride) fluorates (−I) have been extensively characterized and applied in various fields of chemistry^6^, ^12^, ^20^, ^24^ little is known about adducts between the heavier halide ions and HF. Slowly cooling a solution of [PPh_4_]X (X=Br, I) in anhydrous HF to −80 °C yields single crystals of [PPh_4_][X(HF)_2_(HX)] (Scheme 3).

**Figure 5:**
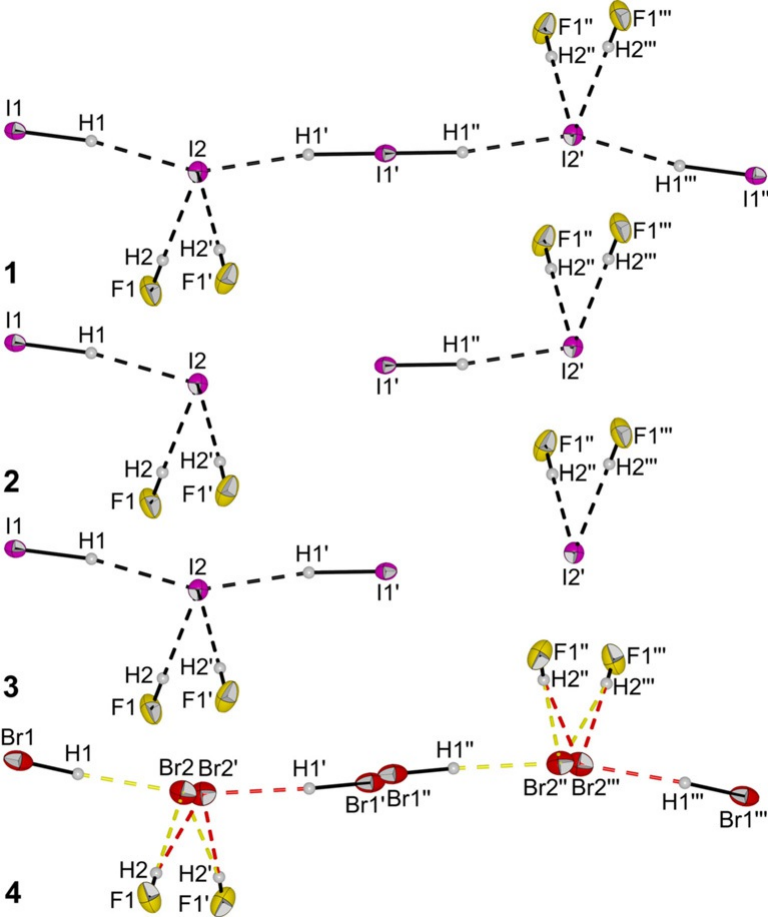
Molecular structure of [PPh_4_][I(HF)_2_(HI)] (**1**) and [PPh_4_][Br(HF)_2_(HBr)] (**4**) in the solid state with thermal ellipsoids shown at 50 % probability. Cations have been omitted for clarity (see Figures S10 and S11 for representation including cations). The positions of Br1, Br2 and H1 (in both structures) have an occupation number of 0.5. Compounds **2** and **3** show two possible descriptions of the anion in [PPh_4_][I(HF)_2_(HI)] as either isolated [I(HF)_2_(HI)]^−^ anions (**2**) or alternating [I(HF)_2_]^−^ and [I(HF)_2_(HI)_2_]^−^ anions (**3**). Selected interatomic distances [pm]: Br2‐F1 300.0(3), Br2‐F1′ 320.2(3), Br2‐Br1 371.9(3), Br2‐Br1′ 416.1(3), I1‐I2 422.1(1), I2‐F1 323.7(2).

The two isostructural salts crystallize in the monoclinic space group *P*2/*n* (Figure 5).

Analyzing the molecular structure of [PPh_4_][I(HF)_2_(HI)] (Figure 5. **1**) in detail reveals that there are different possibilities to describe the structure of the anion. The position of H1 can only have an occupation number of 0.5 to obtain an overall neutral compound. Different occupations of the H1 positions lead to two possible descriptions of the anion structure. An alternating occupation of H1 (**1**: H1, H1′′ occupied, H1′, H1′′′ unoccupied) leads to isolated [I(HF)_2_(HI)]^−^ units (**2**). In comparison, when two neighboring H1 positions are occupied (**1**: H1, H1′ occupied, H1′′ and H1′′′ unoccupied) alternating [I(HF)_2_]^−^ and [I(HF)_2_(HI)_2_]^−^ anions are present (**3**). Vibrational spectroscopy is the method of choice for a further analysis of the described compound. The Raman spectrum of a single crystal of [PPh_4_][I(HF)_2_(HI)] shows three bands (2017, 1963 and 1904 cm^−1^) in the region of the HI stretching mode (Figure S18). The position of these bands correlates well with the calculated bands (MP2/def2‐TZVPP) for the [I(HF)_2_(HI)_2_]^−^ anion while the number of bands indicates a mixture of [I(HF)_2_(HI)]^−^ and [I(HF)_2_(HI)_2_]^−^ anions within the crystal.

[PPh_4_][Br(HF)_2_(HBr)] is isostructural to [PPh_4_][I(HF)_2_(HI)]. Besides the occupational disorder of H1, the molecular structure of [PPh_4_][Br(HF)_2_(HBr)] exhibits a positional disorder of Br1 and Br2. The disorder of the bromine atoms results in two symmetry‐equivalent, pyramidal anions (Figure 5. **4**, red and yellow lines). Due to the disorder of Br2 a [Br(HF)_2_(HBr)_2_]^−^ anion is conceivable which would have two Br‐Br distances of *R*(Br1‐Br2)=371.9(3) pm and *R*(Br2‐Br1′)=416.1(3) pm which differ by 45 pm. The calculated (B3LYP(D3BJ)/def2‐TZVPP (SCS‐MP2/def2‐TZVPP)) distances of 358.7 (355.2) pm for [Br(HF)_2_(HBr)]^−^ and 367.8 (369.5) pm for [Br(HF)_2_(HBr)_2_]^−^ are significantly shorter than the Br2‐Br1′ distance which might indicate only a weak bonding interaction. Therefore the pyramidal description as [Br(HF)_2_(HBr)]^−^ anions seems to be more reasonable. Unfortunately, no bands for the HBr stretching modes were observed in the Raman spectrum of [PPh_4_][Br(HF)_2_(HBr)] which might be due to the low Raman intensity of these bands (Figure S20). Therefore, there is no clear evidence whether isolated [Br(HF)_2_(HBr)]^−^ anions or alternating [Br(HF)_2_(HBr)_2_]^−^ and [Br(HF)_2_]^−^ units are present within the crystal.

**Scheme 4:**
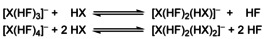
Equilibrium between the homoleptic [X(HF)_*n*_]^−^ and the heteroleptic [X(HF)_*n*−*m*)_(HX)_*m*_]^−^.

The formation of heteroleptic adducts between halides and halogen halides is unexpected as the hydrogen bond donor strength of the halogen halides decreases from HF to HI due to the decreasing polarization of the HX bond. An adduct between the halide ion and the strongest hydrogen bond donor should be most favorable which should favor the formation of homoleptic complexes. The exchange of one HF against one HX molecule (X=Cl, Br, I) was calculated to be 2 to 7 kJ mol^−1^ endergonic for [X(HF)_3_]^−^ while the exchange of two HF against two HX in [X(HF)_4_]^−^ was calculated to be 10 to 24 kJ mol^−1^ endergonic (Scheme 4, Table S12 and S13). This indicates that in solution the most stable anions are of the [X(HF)_*n*_]^−^ type while the small energy differences between the homoleptic and heteroleptic complexes can be compensated by small interactions within the crystal.

When halide salts are dissolved in aHF there are in general three possible scenarios depending on the relative acidities of the hydrogen halide and HF:

1. *HF is a stronger acid than the hydrogen halide*: The halide will be completely protonated forming HX and F^−^. Therefore, ions of the type [F(HF)_*n*_]^−^ should be the dominant anionic species in solution; HX is also present in solution.
2. *HF is a weaker acid than the hydrogen halide*: The halide will not be protonated but solvated by the HF molecules. Ions of the type [X(HF)_*n*_]^−^ should be the dominant anionic species in solution; no HX is present.
3. *HF and HX in HF have similar acidities*: The halide ion will be partially protonated forming HX and F^−^. As a consequence, ions of the type [X(HF)_*n*_]^−^ and [F(HF)_*n*_]^−^ should be present in solution; HX is present as well.

**Scheme 5:**
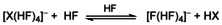
Equilibrium between solvated X^−^ and F^−^ in HF.

The obtained molecular structure of [PPh_4_][X(HF)_2_(HX)] (X=Br, I) indicates that scenario 3 is the preferred one for HBr and HI. To enable the crystallization of [PPh_4_][X(HF)_2_(HX)] (X=Br, I) sufficient quantities of X^−^, HX and HF have to be present in solution. When [PPh_4_]X and HF are used as starting materials this can only be the case when there is an equilibrium between X^−^ and HX in solution. Because the concentration of the salt in HF is very large (8 equiv HF per X^−^) the influence of the concentration on the equilibrium should be rather small. The formed HX is mostly dissolved in HF and since the reaction is performed in a closed reaction vessel the shift of the equilibrium by removal of HX by evaporation can also be neglected. Therefore, it can be concluded that HBr/HI in aHF should have similar acidities as aHF. To further verify this hypothesis the free reaction energies and equilibrium constants for the protonation reaction of X^−^ in HF were calculated (Tables S7–S10). Gas‐phase calculations on the B3LYP(B3BJ)/def2‐TZVPP (SCS‐MP2/def2‐TZVPP) level of theory predict a free reaction energy of 171.81 (173.96) kJ mol^−1^ (X=Cl) to 266.50 (270.64) kJ mol^−1^ (X=I) for the protonation reaction of X^−^ with HF (see Table S 9). This large discrepancy from the expected ≈0 kJ mol^−1^ for an equilibrium reaction can be explained by not taking solvation into account. The high acidity of aHF results from the high solvation energy of the fluoride ion in HF, which is formed during the protonation reaction. Therefore, a proper consideration of solvation effects is essential for a correct description of this system. The solvation of the molecules was therefore modeled by an explicit calculation of the first solvation shell of the halide ions, which was estimated to consist of four HF molecules, and solvation was additional treated using the Cosmo model (Scheme 5).

Free reaction energies of 3.0 kJ mol^−1^ for X=Cl (*K*_eq_=0.29, ratio HCl/Cl^−^=0.54 for *c*_0_(HF)=*c*_0_(Cl^−^), Scheme S1), 14.8 kJ mol^−1^ for X=Br (*K*_eq_=0.0025, ratio HBr/Br^−^=0.05 for *c*_0_(HF)=*c*_0_(X^−^) and 7.4 kJ mol^−1^ for X=I (*K*_eq_=0.050, ratio HI/I^−^=0.22 for *c*_0_(HF)=*c*_0_(X^−^)) have been calculated (SCS‐MP2(COSMO)/def2‐TZVPP) for the protonation reactions in HF. The calculated free reaction energies are decently close to 0; this supports the thesis that all hydrogen halides have similar acidities in HF. In addition, the calculated ratios between HX and X^−^ indicate that in solution sufficient quantities of X^−^, HF and HX should be present to enable the crystallization of [PPh_4_][X(HF)_2_(HX)].

### Further investigations on hydrogen bond donors and acceptors

**Scheme 6:**
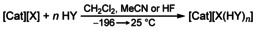
Synthesis of hydrogen‐bonded adducts between anions (X=Br, I, ClO_4_) and HY (Y=F, Br, CN). [Cat]=[PPh_4_]^+^, [PPN]^+^.

Besides the halide‐HCl and halide‐HF systems, further investigations have been carried out on weaker hydrogen bond donors and acceptors. Therefore, hydrogen bromide and hydrogen cyanide have been tested as hydrogen bond donors. In addition, the hydrogen bond acceptor abilities of the perchlorate anion have been investigated (Scheme 6).

**Figure 6:**
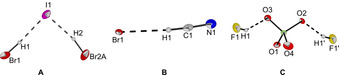
Molecular structures of A) [PPh_4_][I(HBr)_2_], B) [PPN][Br(HCN)] and C) [PPh_4_][ClO_4_(HF)_2_] in the solid state with thermal ellipsoids shown at 50 % probability. Cations and disorders have been omitted for clarity (see Figures S13 to S15 for representations including cations and disorders). Selected interatomic distances [pm]: Br1‐I1 387.1(1), Br2A‐I1 373.4(2), Br1‐C1 340.5(6), F1‐O3 256.1(4), F1′‐O2 255.7(5), Cl1‐O1 143.2(6), Cl1‐O2 145.9(7), Cl1‐O3 145.0(6), Cl1‐O4 143.6(4), F1‐Cl1 353.3(4), F1′‐Cl1 352.3(4).

The molecular structures of [PPh_4_][I(HBr)_2_] (*Cc*), [PPN][Br(HCN)] (*Pca*2_1_) and [PPh_4_][ClO_4_(HF)_2_] (*C*2/*c*) have been determined by XRD (Figure 6).

Hydrogen bromide is a weaker hydrogen bond donor compared to HCl and HF and only two examples of halide HBr adducts are known in the literature ([BrHBr]^−[10]^ and [Br(HBr)_2_]^−[11]^). The reaction of bromide salts with over‐stoichiometric amounts of HBr leads to the formation of rather instable compounds which could not be characterized while the reaction of [PPh_4_]I with four equivalents of HBr and slowly cooling to −40 °C led to the crystallization of [PPh_4_][I(HBr)_2_]. In the crystal the [I(HBr)_2_]^−^ anion shows an asymmetrically V‐shaped structure. The obtained Br−I distances of 373.4(2) and 387.1(1) pm are in good agreement with the distance calculated on the B3LYP(D3BJ)/def2‐TZVPP (SCS‐MP2/def2‐TZVPP) level of theory of 379.5 pm (381.7 pm).

**Scheme 7:**
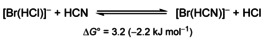
Comparison of the stabilities of [Br(HCN]^−^ and [Br(HCl)].

Recently it was shown that BrCN forms adducts with bromide to form [Br(BrCN)_*n*_]^−^ anions (*n=*1, 3) by halogen bonding interactions.^32^ Therefore it was tested whether similar hydrogen‐bonded compounds can be synthesized. [PPN][Br(HCN)] was obtained by the reaction of [PPN]Br with four equivalents of HCN. The nearly linear geometry of the anion is in agreement with a strong hydrogen bond interaction. This observation is also supported by quantum‐chemical calculations (B3LYP(D3BJ)/def2‐TZVPP (SCS‐MP2/def2‐TZVPP)) which show that the bromide HCN adduct has a similar stability as [BrHCl]^−^ (Scheme 7) of which the hydrogen bond energy was experimentally determined to be 54 kJ mol^−1^.^16^

The equilibrium constant for the protonation reaction of the perchlorate ion in HF forming bifluoride and perchloric acid was determined to be *K*_eq_=(7.5±1.5) 10^−5^ L mol^−1^ by Raman spectroscopy.^33^ Therefore perchlorate is a promising candidate to act as a hydrogen bond acceptor in HF without forming higher amounts of fluoride ions. When [PPh_4_][ClO_4_] was treated with 8 equivalents of HF and cooled to −80 °C single crystals of [PPh_4_][ClO_4_(HF)_2_] were obtained. The short F−O distances of 255.7(5) to 256.1(4) pm are in good agreement with the calculated distances (B3LYP/def2TZVPP and MP2/def2‐TZVPP) of 259.6 (261.5) pm and indicate a strong hydrogen bond interaction. This is also in agreement with thermochemical calculations on the B3LYP(D3BJ)/def2‐TZVPP (SCS‐MP2/def2‐TZVPP) level of theory which predict [ClO_4_(HF)_2_]^−^ to be −75.5 (−61.1) kJ mol^−1^ more stable with respect to the decomposition into HF and ClO_4_^−^.

## Conclusions

By using weakly coordinating cations ([PPh_4_]^+^, [PPN]^+^) eight hitherto unknown poly(hydrogen halide) halogenate (−I) anions ([X(HCl)_4_]^−^ (X=Br, I), [I(HCl)_3_]^−^], [I(HBr_2_]^−^, [X(HF)_2_(HX)]^−^ (X=Br, I), [Br(HCN)]^−^ and [ClO_4_(HF)_2_]^−^) were synthesized and thoroughly characterized by X‐ray diffraction, Raman spectroscopy, and quantum‐chemical calculations. The measured Raman spectra of [PPh_4_][X(HCl)_*n*_] (X=Cl, Br (*n*=4), I (*n*=3)) were used to investigate the influence of the central base and the coordination number on the hydrogen bond strength. It was observed that the hydrogen bond energy decreases from chloride to iodide as a central base, and with increasing coordination number. In addition, quantum‐chemical calculations on halide ions in aHF were carried out. These calculations show that HF and HX (X=Cl, Br, I) have a similar acidity in aHF, which explains why mixed poly(hydrogen halide) halogenate anions [X(HF)_2_(HX)]^−^ (X=Br, I) were obtained starting from [PPh_4_][X] and HF. Furthermore, it was shown that weaker hydrogen‐bond donors, like HBr and HCN can form adducts with halide ions. Additionally, it was observed that even the perchlorate anion, which is often used as a weakly coordinating anion, forms hydrogen‐bonded adducts with HF.

## Experimental Section

**Apparatus and materials**: All substances sensitive to water and oxygen were handled under an argon atmosphere using standard Schlenk techniques and oil pump vacuum up to 10^−3^ mbar. Reactions with HF as a solvent or reactant were performed on a stainless steel vacuum line in self‐built reactors consisting of 8 mm o. d. PFA (perfluoroalkoxy alkanes) tubing which where heat sealed on one end and connected to a steel valve on the other end. Dry MeCN and CH_2_Cl_2_ were obtained by distillation from P_4_O_10_. *n*‐Pentane was dried over molecular sieve. All solvents were stored over activated 3 Å molecular sieves. Commercially available [AsPh_4_]Cl, [PPh_4_]X (X=Cl, Br, I), [PPN]Cl (PPN=bis(triphenylphosphoranylidene)iminium), HF, HCl and HBr were used without further purification. [PPN]Br.^34^ [PPN]I,^35^ [PPh_4_][ClO_4_]^36^ and HCN^37^ were prepared according to literature procedures. All salts were dried in vacuo at 100 °C for 10 min prior to use. Raman spectra were recorded on a Bruker MultiRAM II equipped with a low‐temperature Ge detector (1064 nm, 100–180 mW, resolution of 4 cm^−1^). Spectra of single crystals were recorded at −196 °C using the Bruker RamanScope III (see part g of the Supporting Information for a description of the method used). X‐ray diffraction data were collected on a Bruker D8 Venture CMOS area detector (Photon 100) diffractometer with Mo_Kα_ radiation. Single crystals were coated with perfluoroether oil at low temperature (−40/−80 °C) and mounted on a 0.1–0.2 mm Micromount. The structures were solved with the ShelXT^38^ structure solution program using intrinsic phasing and refined with the ShelXL^39^ refinement package using least squares on weighted F2 values for all reflections using OLEX2.^40^ For quantum chemical calculations (structure optimization (with and without solvent model COSMO^41^) and frequency calculations (including Raman intensities)) the program package TURBOMOLE 7.3^42^ was used. Functionals (B3LYP(D3BJ)^43^ and SCS‐MP2^44^) and the basis set (def2‐TZVPP)^45^ were used as implemented in TURBOMOLE. Minima on the potential energy surface were characterized by harmonic vibrational frequency analysis. Thermochemistry was provided with zero‐point vibration correction, Δ*G* values were calculated at 298.15 K and 1.0 bar.

**[Cat][X(HCl)**_***n***_ **)]**: In a typical experiment 0.4 mmol [Cat]X (Cat=PPN^+^, PPh_4_^+^, AsPh_4_^+^ (only for X=Cl^−^); X=Cl^−^, Br^−^, I^−^) were dissolved in 0.2 mL CH_2_Cl_2_. 1.6 mmol HCl were condensed onto the obtained suspension and the reaction mixture was allowed to warm to room temperature. A clear solution was obtained after carefully heating to a maximum of 40 °C and mechanically agitating. Colorless single crystals of [PPN][X(HCl)_4_] (X=Cl^−^, Br^−^, I^−^), [PPh_4_][X(HCl)_4_] (X=Cl^−^, Br^−^), [PPh_4_][I(HCl)_3_] and [AsPh_4_][Cl(HCl)_4_] were obtained by slowly cooling the reaction mixture to −40 °C.

**[AsPh_4_][Cl(HCl)_4_]**: CCDC number: 1995595

**[PPh_4_][Cl(HCl)_4_]**: Raman (−196 °C): ṽ=3083 (w), 3068 (m), 3059 (m), 2523* (w) 2318* (w), 1589 (m), 1576 (w), 1187 (w), 1164 (w), 1114 (w), 1102 (w), 1024 (w), 1001 (s), 727 (w), 682 (w), 615 (w), 295 (w), 251 (w), 200 cm^−1^ (w). CCDC number: 1995600.

**[PPh_4_][Br(HCl)_4_]**: Raman (−196 °C): ṽ=3081 (w), 3068 (m), 3059 (m), 2525* (w) 2370* (w), 1589 (m), 1577 (w), 1186 (w), 1164 (w), 1114 (w), 1102 (w), 1025 (w), 1001 (s), 728 (w), 702 (w) 682 (w), 616 (w), 296 (w), 252 (w), 199 cm^−1^ (w). CCDC number: 1995596.

**[PPh_4_][I(HCl)_3_]**: Raman (−196 °C): ṽ=3084 (w), 3063 (s), 2388* (m), 2309* (m), 2232* (m), 1588 (m), 1575 (w), 1184 (w), 1164 (w), 1112 (w), 1102 (w), 1028 (w), 1000 (s), 681 (w), 292 (w), 253 (w), 198 cm^−1^ (w). CCDC number: 1995597.

**[PPN][Cl(HCl)_4_]**: Raman (−196 °C): ṽ=3067 (m), 3057 (m), 1589 (m), 1114 (w), 1027 (w), 1001 (s) 669 (w), 616 (w), 240 cm^−1^ (w). CCDC number: 1995602.

**[PPN][Br(HCl)_4_]**: Raman (−196 °C): ṽ=3065 (s), 3056 (s), 1590 (m), 1576 (w), 1114 (w), 1026 (w), 1001 (s) 701 (w), 669 (w), 617 (w), 287 (w), 269 (w), 240 cm^−1^ (w). CCDC number: 1995603.

**[PPN][I(HCl)_4_]**: Raman (−196 °C): ṽ=3058 (s), 2472 (w), 2415 (w), 2301 (w), 1590 (m), 1575 (w), 1182 (w), 1114 (w), 1026 (w), 1001 (s), 809 (w), 751 (w), 668 (w), 617 (w), 238 cm^−1^ (w) CCDC number: 1995604.

Bands marked with an asterisk belong to the [X(HCl)_*n*_]^−^ species.

**[PPh_4_][I(HBr)_2_]**: In a typical experiment [PPh_4_]I (0.4 mmol, 186 mg, 1 equiv) was dissolved in 0.2 mL CH_2_Cl_2_. HBr (1.6 mmol, 4 equiv) was condensed onto the obtained suspension and the reaction mixture was allowed to warm to room temperature. A clear solution was obtained after carefully heating to a maximum of 40 °C and mechanically agitating. Colorless single crystals of [PPh_4_][I(HBr)_2_] and [AsPh_4_][Cl(HCl)_4_] were obtained by slowly cooling the reaction mixture to −40 °C. CCDC number: 1997012.

**[PPN][BrHBr]⋅CH_2_Cl_2_**: In a typical experiment [PPN]Br (0.4 mmol, 246 mg, 1 equiv) was dissolved in 2 mL CH_2_Cl_2_. HBr (1.6 mmol, 4 equiv) was condensed onto the obtained solution, and the reaction mixture was allowed to warm to room temperature. Colorless single crystals of [PPN][BrHBr]⋅CH_2_Cl_2_ were obtained by vapor diffusion of *n*‐pentane into the CH_2_Cl_2_ solution at room temperature. CCDC number: 1995599.

**[PPh_4_][X(HX)(HF)_2_]**: In a typical experiment 0.4 mmol [PPh4]X (X=Cl^−^, Br^−^, I^−^) were filled into an 8 mm o.d. PFA tubing which was heat sealed on one end. The PFA tube was connected to a steel valve and HF (3.2 mmol, 64 mg, 8 equiv) was condensed onto the solid. The reactor was flame‐sealed and mechanically agitated until a clear solution was obtained. Colorless single crystals of [PPh_4_][X(HX)(HF)_2_] (X=Cl^−^, Br^−^, I^−^) were obtained by slowly cooling the reaction mixture to −80 °C.

**[PPh_4_][I(HF)_2_(HI)]**: Raman (−196 °C): ṽ=3065 (m), 2017* (m), 1963* (m), 1904* (m), 1589 (m), 1575 (w), 1189 (w), 1165 (w), 1103 (w), 1028 (w), 1001 (s), 671 (w), 616 (w), 329 (w), 252 (w), 199 (w), 164 cm^−1^ (w). CCDC number: 1995598.

**[PPh_4_][Br(HF)_2_(HBr)]**: Raman (−196 °C): ṽ=3070 (m), 1590 (s), 1577 (w), 1487 (vw), 1439 (vw), 1382 (w), 1343 (vw), 1319 (vw), 1305 (vw), 1215 (vw), 1191 (w), 1166 (vw), 1112 (w), 1101 (m), 1028 (m), 1002 (vs.), 733 (s), 681 (w), 616 (w), 576 (vw), 385 (w), 294 (m), 250 (m), 198 (m). CCDC number: 1995605.

Bands marked with an asterisk belong to the [X(HF)_2_(HX)]^−^ species.

**[PPh_4_][ClO_4_(HF)_2_]**: In a typical experiment [PPh4][ClO_4_] (0.4 mmol, 175 mg, 1 equiv) was filled into an 8 mm o.d. PFA tubing which was heat sealed on one end. The PFA tube was connected to a steel valve and HF (3.2 mmol, 64 mg, 8 equiv) was condensed onto the solid. The reactor was flame‐sealed and mechanically agitated until a clear solution was obtained. Colorless single crystals of [PPh_4_][ClO_4_(HF)_2_] were obtained by slowly cooling the reaction mixture to −80 °C. CCDC number: 1995606.

**[PPN][Br(HCN)]**: In a typical experiment [PPN]Br (0.4 mmol, 246 mg, 1 equiv) was dissolved in 0.4 mL MeCN. HCN (1.6 mmol, 4 equiv) was condensed onto the obtained suspension and the reaction mixture was allowed to warm to room temperature. Colorless single crystals of [PPN][Br(HCN)] were obtained by slowly cooling to −40 °C. CCDC number: 1995601.

**Crystallographic data**: Deposition numbers 1995595 (for [AsPh_4_][Cl(HCl)_4_]), 1995596 (for [PPh_4_][Br(HCl)_4_]), 1995597 (for [PPh_4_][I(HCl)_3_]), 1995598 (for [PPh_4_][I(HF)_2_(HI)]), 1995599 (for [PPN][BrHBr]⋅CH_2_Cl_2_), 1995600 (for [PPh_4_][Cl(HCl)_4_]), 1995601 (for [PPN][Br(HCN)]), 1995602 (for [PPN][Cl(HCl)_4_]), 1995603 (for [PPN][Br(HCl)_4_]), 1995604 (for [PPN][I(HCl)_4_]), 1995605 (for [PPh_4_][Br(HF)_2_(HBr)]), 1995606 (for [PPh_4_][ClO_4_(HF)_2_]), and 1997012 (for [PPh_4_][I(HBr)_2_]) contain the supplementary crystallographic data for this paper. These data are provided free of charge by the joint Cambridge Crystallographic Data Centre and Fachinformationszentrum Karlsruhe Access Structures service www.ccdc.cam.ac.uk/structures.

## Conflict of interest

The authors declare no conflict of interest.

## Supporting information

As a service to our authors and readers, this journal provides supporting information supplied by the authors. Such materials are peer reviewed and may be re‐organized for online delivery, but are not copy‐edited or typeset. Technical support issues arising from supporting information (other than missing files) should be addressed to the authors.

Supplementary
