# Supplementary material for: Synthesis and Characterization of Poly(hydrogen halide) Halogenates (–I)
Source: Chemistry. 2020 Sep 16;26(58):13256–63. doi: 10.1002/chem.202001864 (PMC7693257; doi:10.1002/chem.202001864)
Supplement: Supplementary file 1 — Supplementary [file CHEM-26-13256-s001.pdf]

Chemistry–A European Journal

Supporting Information

Synthesis and Characterization of Poly(hydrogen halide) Halogenates (–I)

Patrick Voßnacker, Simon Steinhauer, Julia Bader, and Sebastian Riedel*[a]

Table of Contents

a) Molecular Structures Including Cations and Disorders 2

a1. $[PPh_4][X(HCl)_n]$ and $[AsPh_4][Cl(HCl)_4]$ 2

a2. $[PPN][X(HCl)_4]$ 4

a3. $[PPh_4][X(HF)_2(HX)]$ 7

a4. Further Hydrogen Bonded Structures 8

b) Experimental and Calculated Raman Spectra 10

c) Quantum Chemically Optimized Structures 14

d) Calculated Energies and Free Reaction Energies 16

d1) B3LYP(D3BJ)/def2-TZVPP Energies 16

d2) SCS-MP2/def2-TZVPP Energies 18

d3) SCS-MP2(COSMO)/def2-TZVPP Energies 20

d4) Free Reaction Energy Calculation 20

d5) Comparison of Different Geometries for the $[X(HCl)_4]^-$ Anion 24

e) Coordinates of Optimized Structures 25

f) Calculated Vibrational Spectra 55

g) Raman Spectra of Samples Cooled to –196 °C 74

a) Molecular Structures Including Cations and Disorders

a1. $[PPh_4][X(HCl)_n]$ and $[AsPh_4][Cl(HCl)_4]$

Table S 1. Comparison of interatomic distances and angles for $[PPh_4][X(HCl)_4]$ (X = Cl, Br).

Property	Cl	Br
Cl-X distance [pm]	340.1(1)	353.4(1)
Cl-X-Cl'' angle [°]	144.3 (1)	145.1(1)

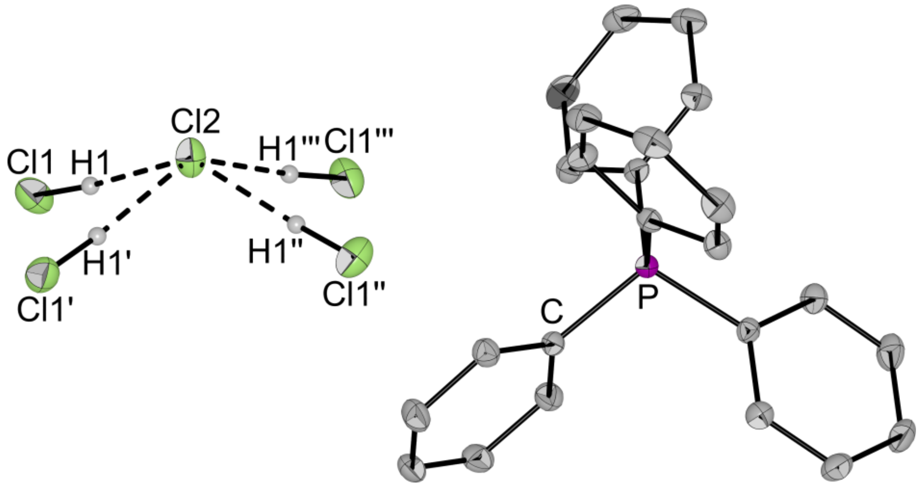

Figure S 1. Molecular structure of $[PPh_4][Cl(HCl)_4]$ in the solid state with thermal ellipsoids shown at 50 % probability.

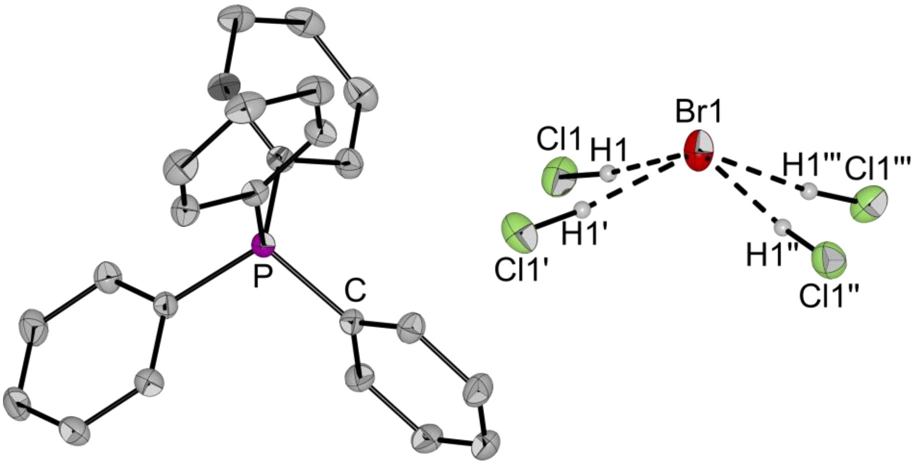

Figure S 2. Molecular structure of $[PPh_4][Br(HCl)_4]$ in the solid state with thermal ellipsoids shown at 50 % probability. A chloride ion occupies the position of Br1 with a probability of 13.7(3) %, which might be explained by a protonation of bromide ions and the formation of Cl^- and HBr.

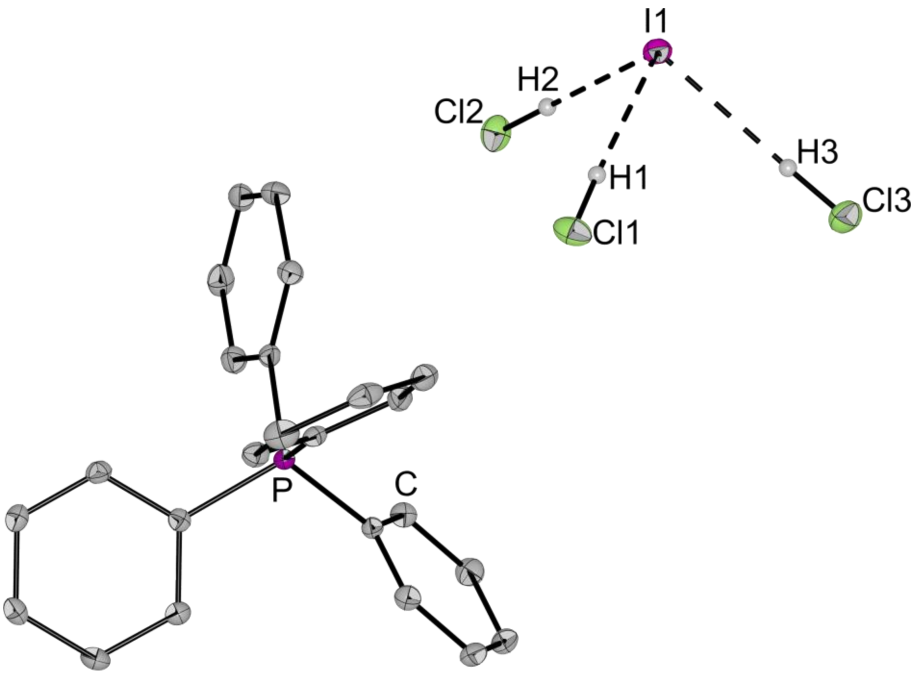

Figure S 3. Molecular structure of [PPh_4][I(HCl)$_3$] in the solid state with thermal ellipsoids shown at 50 % probability.

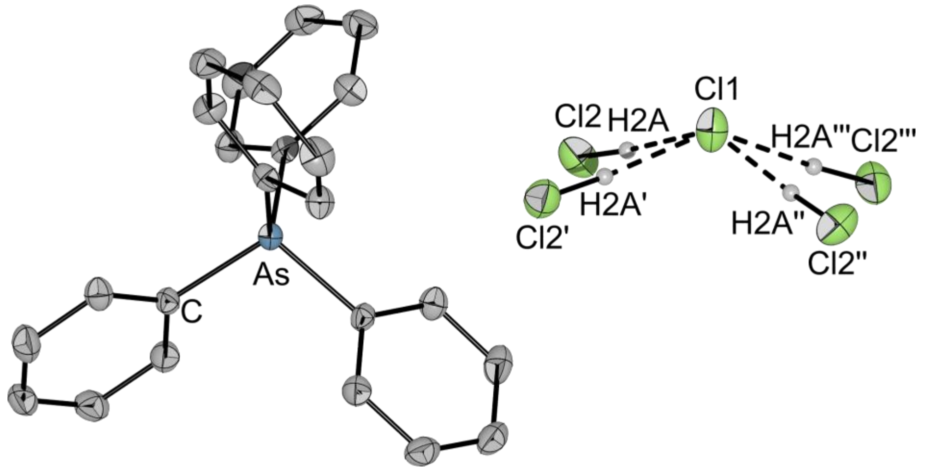

Figure S 4. Molecular structure of [$AsPh_4$][Cl(HCl)$_4$] in the solid state with thermal ellipsoids shown at 50 % probability. A bromide ion occupies the position of Cl1 with a probability of 13.5(3) %, which might be explained by a contamination of the starting material.

a2. [PPN][X(HCl)$_4$]

Table S 2. Comparison of interatomic distances and angles for [PPN][X(HCl)$_4$] (X = Cl, Br,I).

Property	Cl	Br	I
Cl-X distance (1) [pm]	334.8(1)	347.6(1)	370.4(2)
Cl-X distance (2) [pm]	341.6(1)	354.0(2)	374.8(2)
Cl-X angle (1) [°]	97.7(1)	97.7(1)	91.6(1)
Cl-X angle (2) [°]	144.1(1)	141.6(1)	138.0(1)
Cl-X angle (3) [°]	84.3(1)	84.3	89.2(1)
Cl-X angle (4) [°]	122.1(1)	122.1(1)	120.9(1)

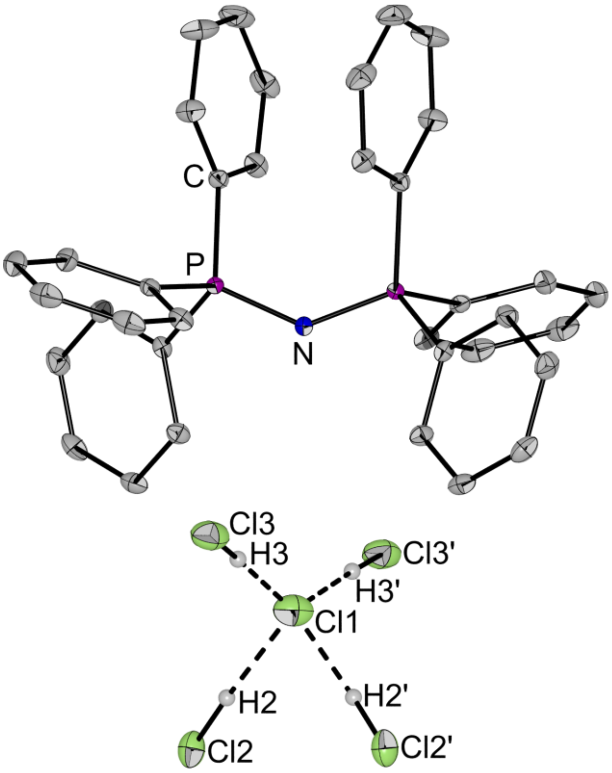

Figure S 5. Molecular structure of [PPN][Cl(HCl)$_4$] in the solid state with thermal ellipsoids shown at 50 % probability.

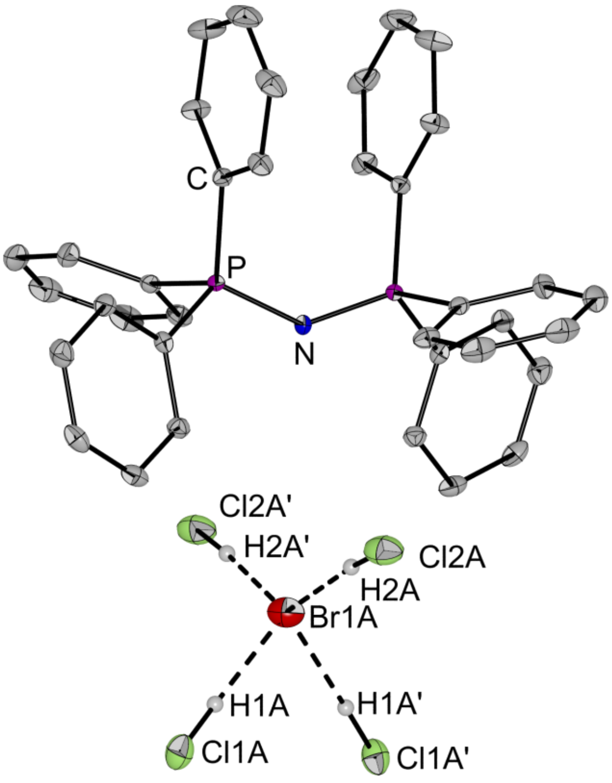

Figure S 6. Molecular structure of [PPN][Br(HCl)$_4$] in the solid state with thermal ellipsoids shown at 50 % probability.

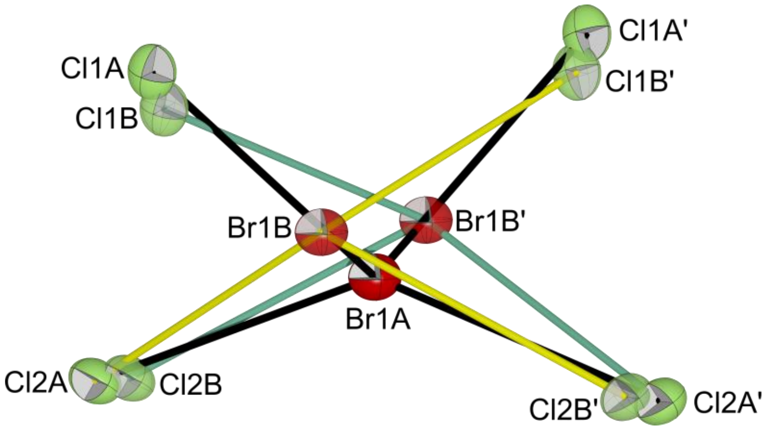

Figure S 7. Representation of the disorder of the anion in the solid state structure of [PPN][Br(HCl)$_4$]. Thermal ellipsoids are shown at 50 % probability. The occupation numbers for the halide atoms were determined to be 0.928(1) for Br1A and Cl1A, 0.964(1) for Cl2A and 0.036(1) for Br1B, Cl1B and Cl2B. Therefore the anion position within the crystal is occupied by a [Br(HCl)$_4$]$^-$ anion (black lines) with a probability of 92.8(1) % and occupied by a [Br(HCl)$_3$]$^-$ anion (yellow and green lines) with 7.2(1) % probability.

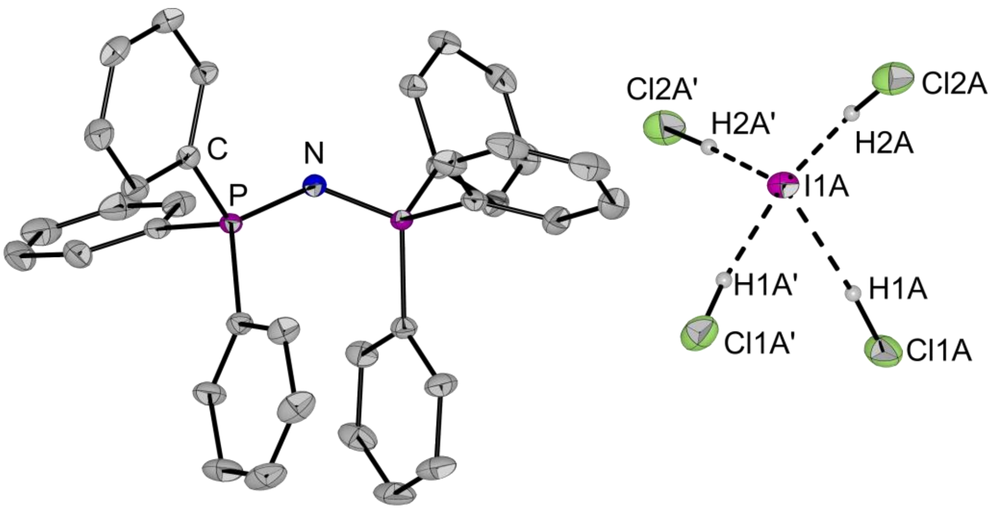

Figure S 8. Molecular structure of [PPN][I(HCl)$_4$] in the solid state with thermal ellipsoids shown at 50 % probability.

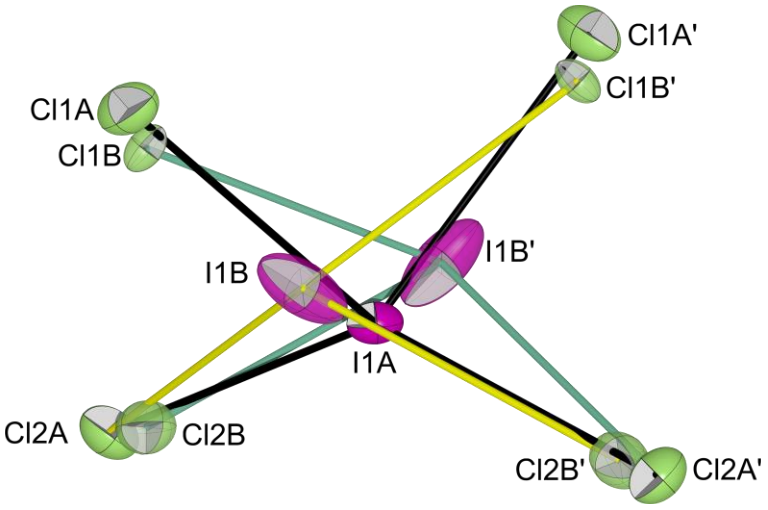

Figure S 9. Representation of the disorder of the anion in the molecular structure of [PPN][Br(HCl)$_4$] in the solid state. Thermal ellipsoids are shown at 50 % probability. The occupation numbers for the halogen atoms were determined to be 0.777(2) for I1A and Cl1A, 0.889(1) for Cl2A and 0.111(1) for I1B, Cl1B and Cl2B. Therefore the anion position within the crystal is occupied by a [I(HCl)$_4$]$^{-}$ anion (black lines) with a probability of 77.7(2) % and occupied by a [I(HCl)$_3$]$^{-}$ anion (yellow and green lines) with 22.2(1) % probability.

a3. [PPh_4][$X(HF)_2(HX)$]

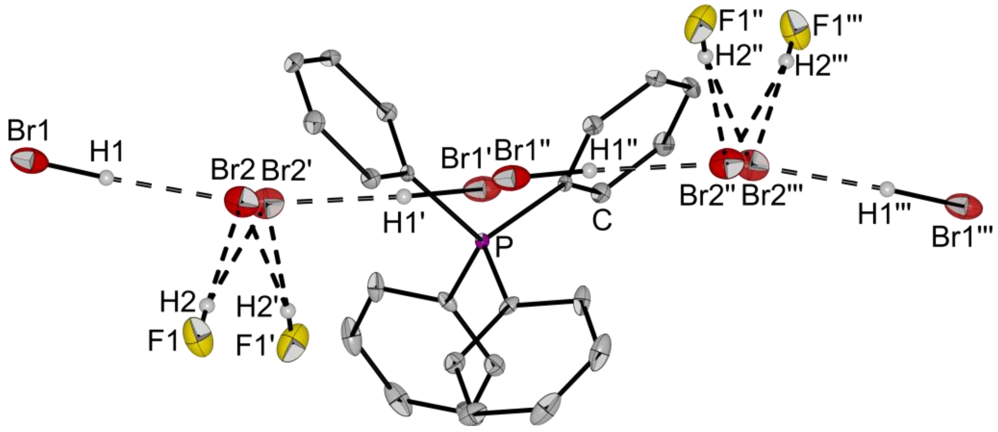

Figure S 10. Molecular structure of [PPh_4][$Br(HF)_2(HBr)$] in the solid state with thermal ellipsoids shown at 50 % probability.

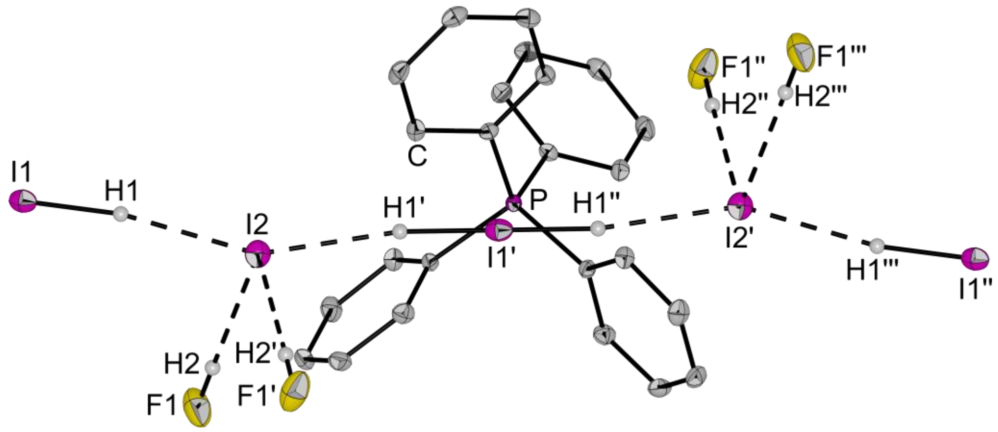

Figure S 11. Molecular structure of [PPh_4][$I(HF)_2(HI)$] in the solid state with thermal ellipsoids shown at 50 % probability.

a4. Further Hydrogen Bonded Structures

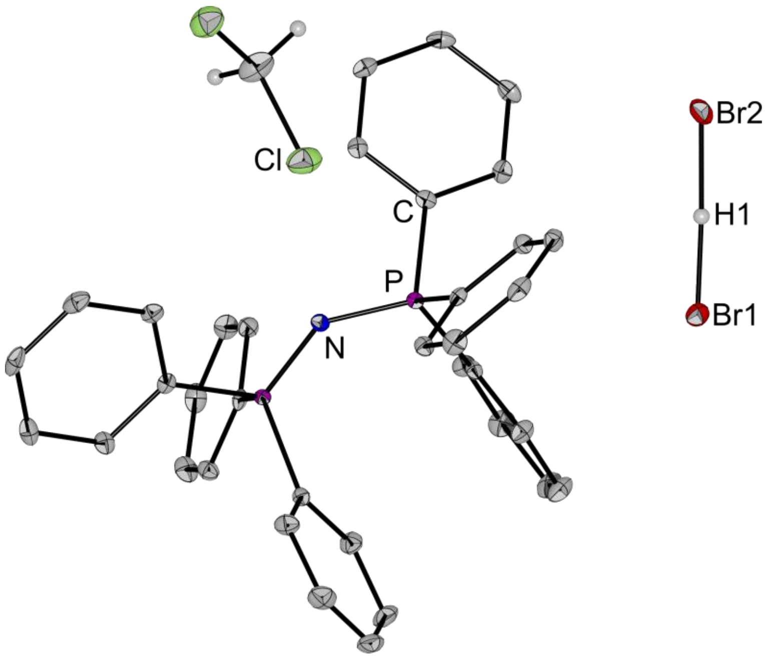

Figure S 12. Molecular structure of [PPN][BrHBr]·CH_2Cl_2 in the solid state with R(Br1-Br2) = 341.6(1) pm. Thermal ellipsoids are shown at 50 % probability.

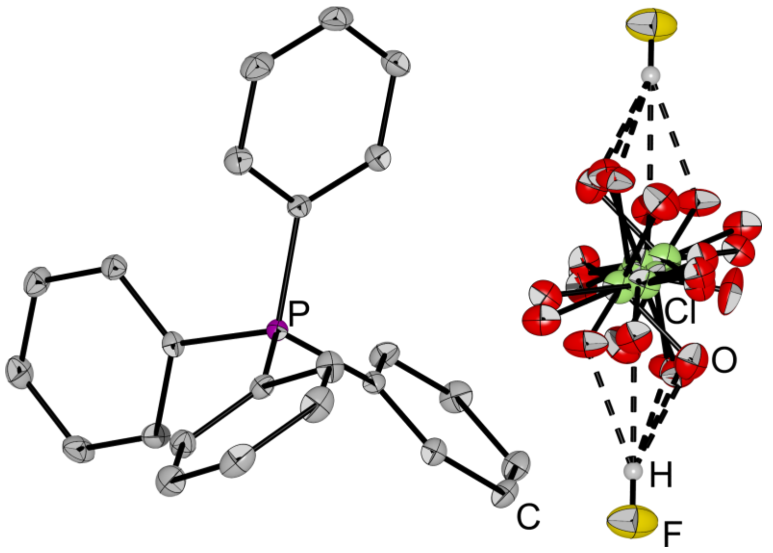

Figure S 13. Molecular structure of [PPh_4][$ClO_4(HF)_2$] in the solid state including the disorder of the perchlorate anion. Thermal ellipsoids are shown at 50 % probability.

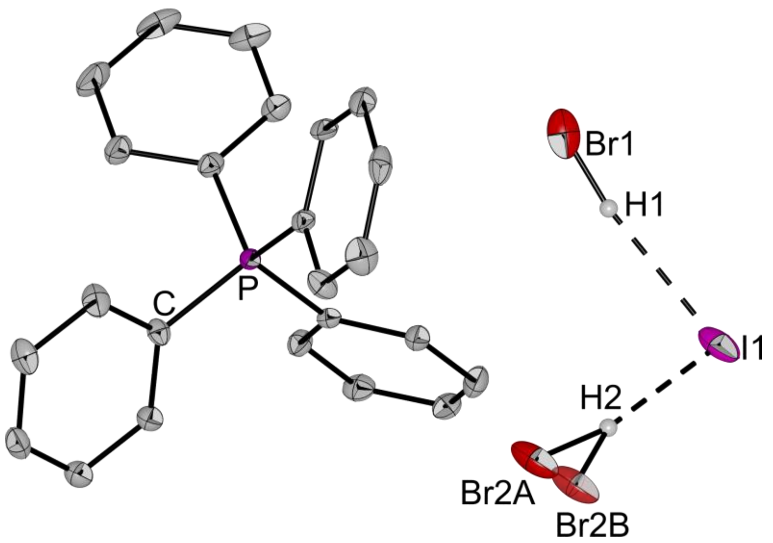

Figure S 14. Molecular structure of $[PPh_4][I(HBr)_2]$ in the solid state including the disorder of the anion. The occupation number of Br2A equals 0.741(3). Thermal ellipsoids are shown at 50 % probability.

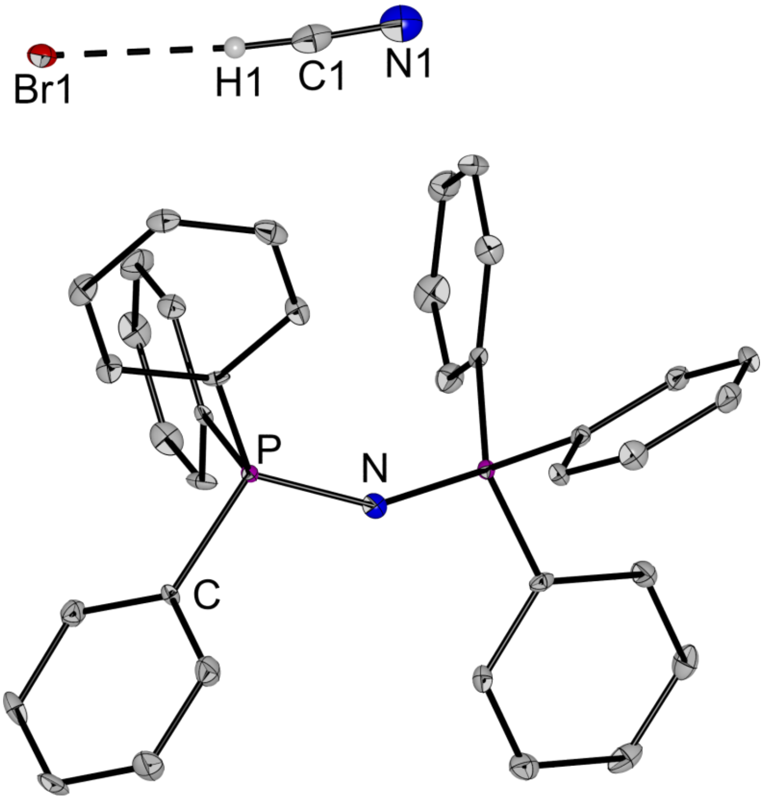

Figure S 15. Molecular structure of [PPN][Br(HCN)] in the solid state. Thermal ellipsoids are shown at 50 % probability.

b) Experimental and Calculated Raman Spectra

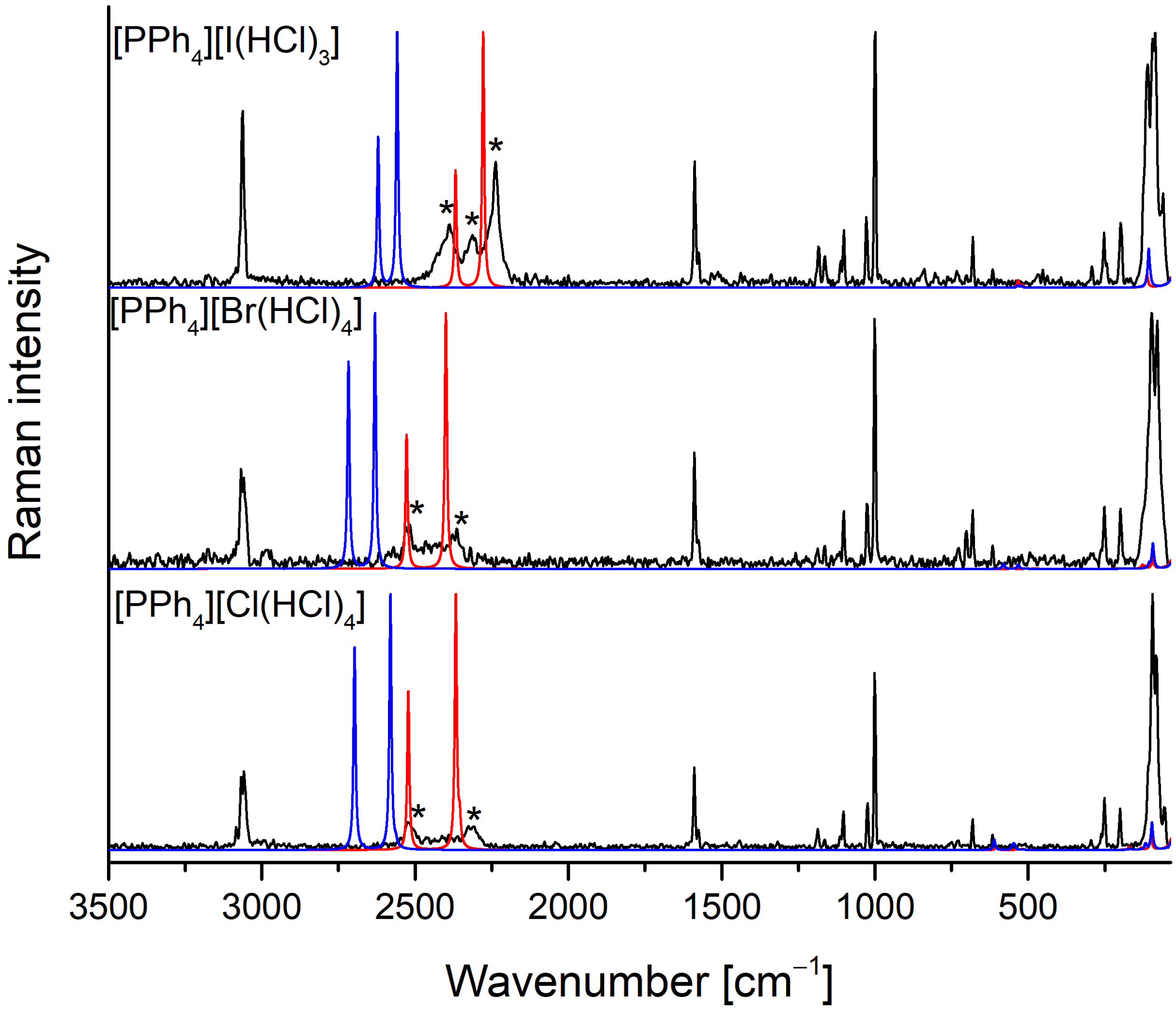

Figure S 16. Experimental (black) Raman spectrum of $[PPh_4][X(HCl)_n]$ (X = Cl, Br, I) and calculated (B3LYP/def2-TZVPP (red) and MP2/def2-TZVPP (blue)) spectra. Bands highlighted with asterisk are associated to $[X(HCl)_n]^-$.

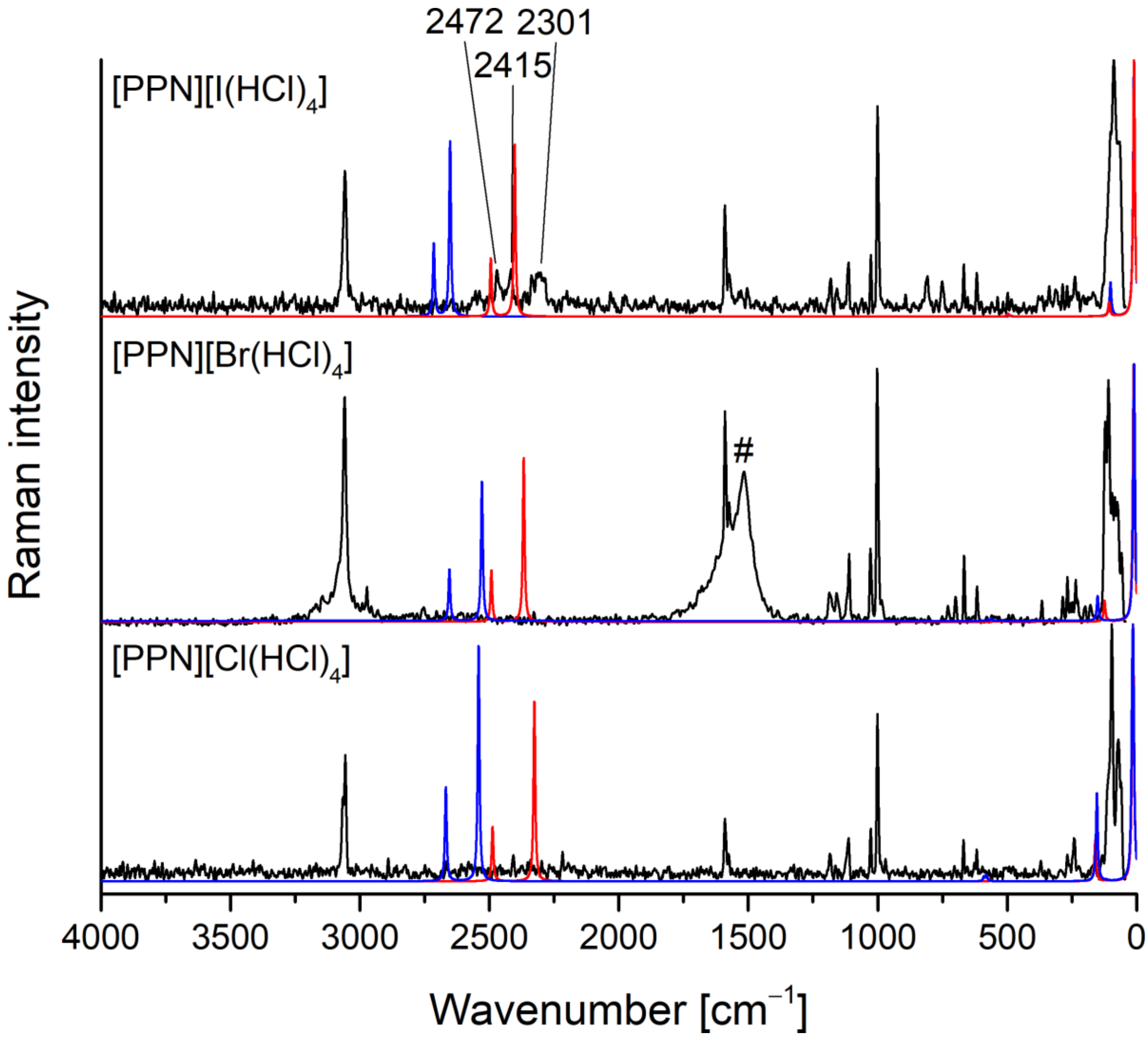

Figure S 17. Experimental (black) Raman spectrum of [PPN][X(HCl)$_4$] (X = Cl, Br, I) and calculated (B3LYP/def2-TZVPP (red) and MP2/def2-TZVPP (blue)) spectra. The band marked with a dagger is due to liquid oxygen resulting from the measurement at –196 °C.

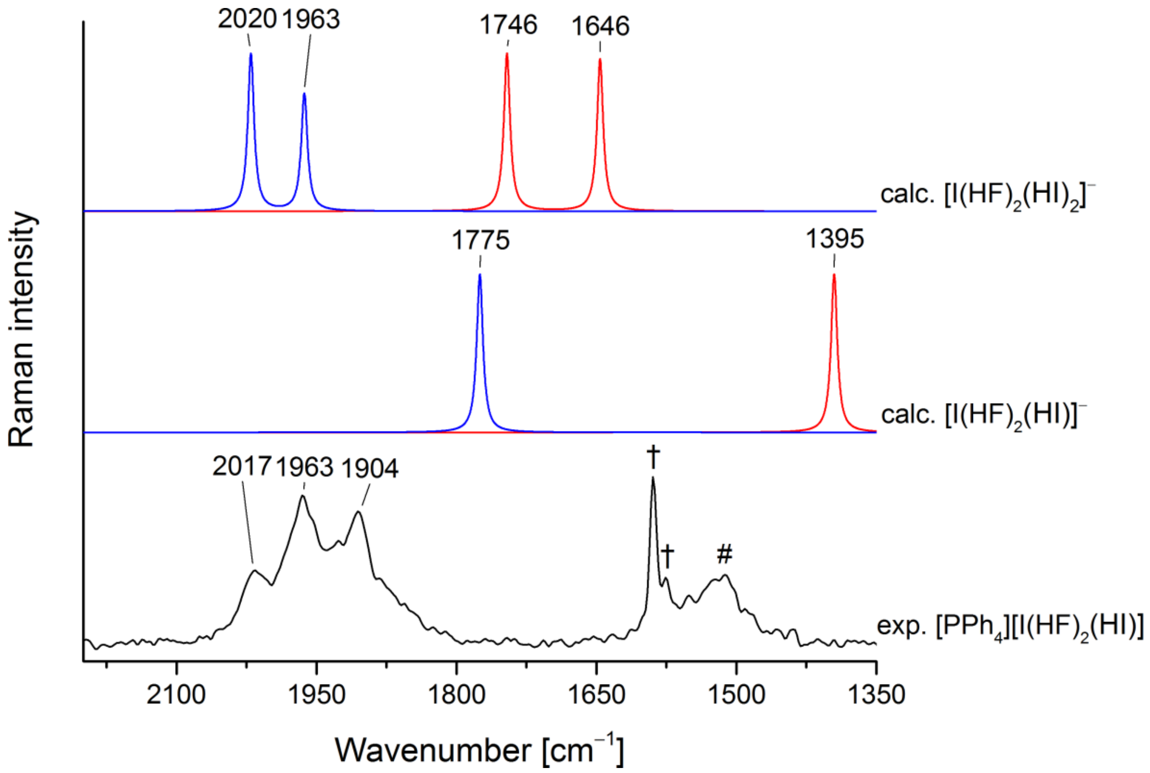

Figure S 18. Experimental (black) Raman spectrum of $[PPh_4][I(HF)_2(HI)]$ and calculated (B3LYP/def2-TZVPP (red) and MP2/def2-TZVPP (blue)) spectra of $[I(HF)_2(HI)]^-$, $[I(HF)_2(HI)_2]^-$. Bands highlighted with a hash belong to the cation, while bands highlighted with a dagger are due to liquid oxygen resulting from the measurement at –196 °C.

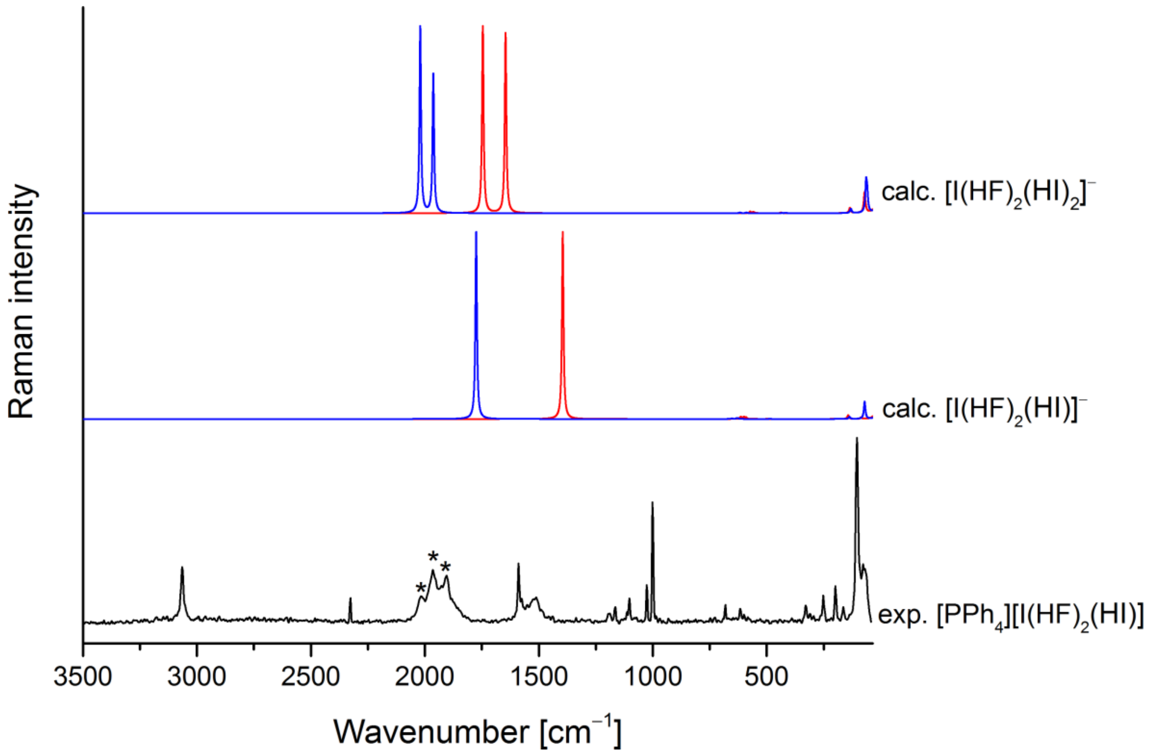

Figure S 19. Experimental (black) Raman spectrum of $[PPh_4][I(HF)_2(HI)]$ and calculated (B3LYP/def2-TZVPP (red) and MP2/def2-TZVPP (blue)) spectra of $[I(HF)_2(HI)]^-$, $[I(HF)_2(HI)_2]^-$. Bands highlighted with an asterisk are associated to $[I(HF)_2(HI)]^-$.

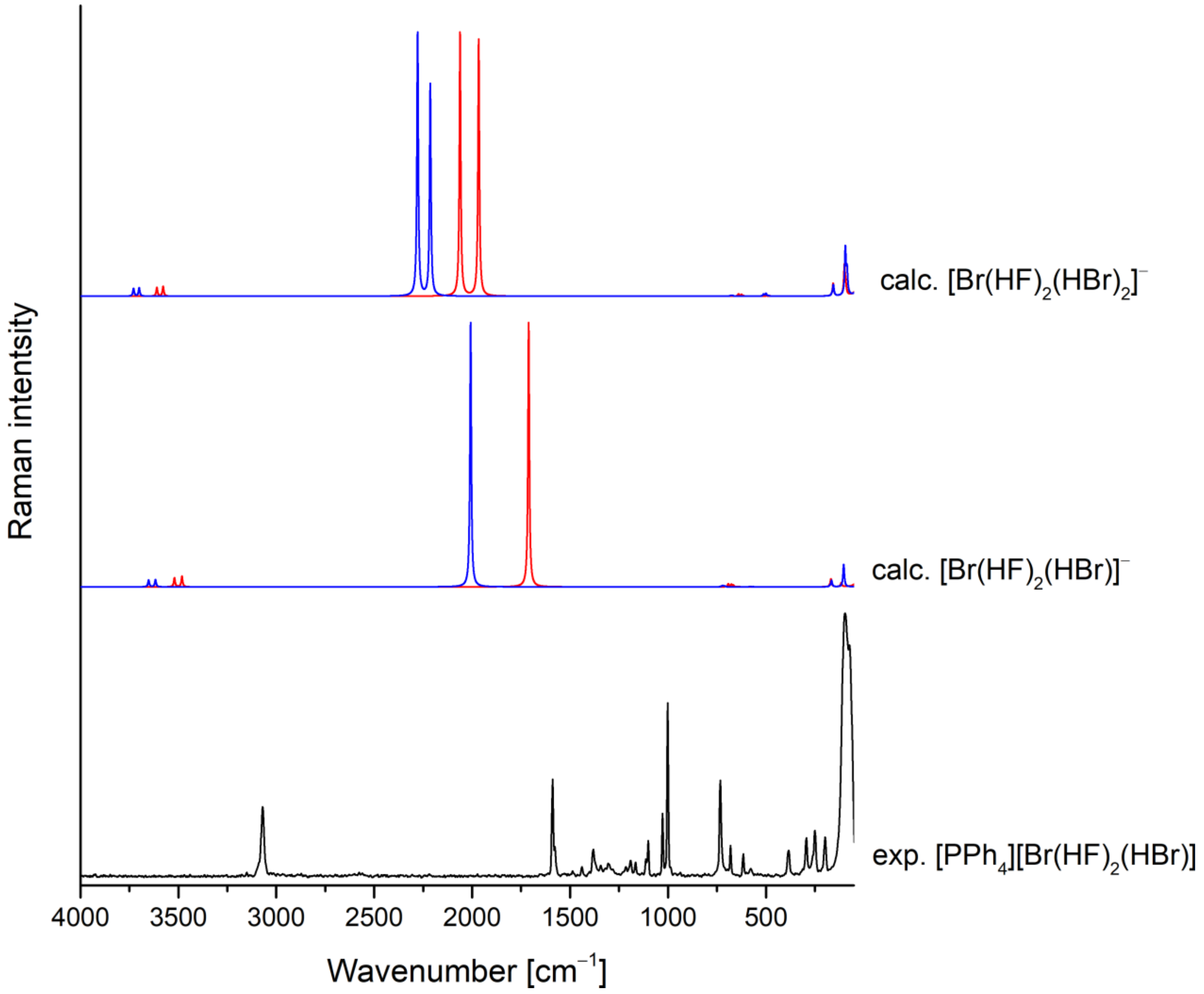

Figure S 20. Experimental (black) Raman spectrum of $[PPh_4][Br(HF)_2(HBr)]$ and calculated (B3LYP/def2-TZVPP (red) and MP2/def2-TZVPP (blue)) spectra of $[Br(HF)_2(HBr)]^-$.

c) Quantum Chemically Optimized Structures

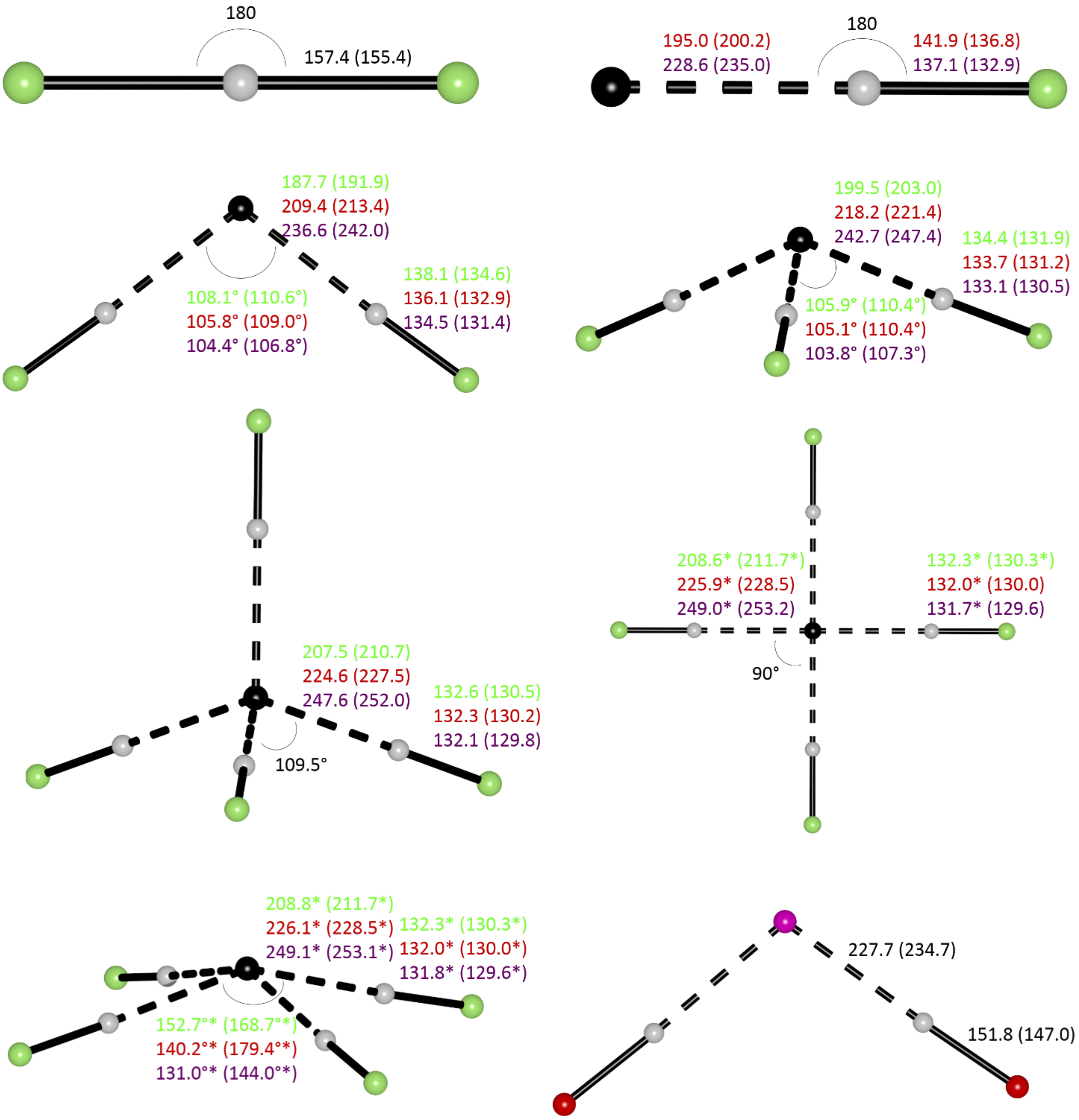

Figure S 21. Optimized structures of $[X(HY)_n]^-$ (n = 1, 3, 4 (X = Cl, Br, I; Y = Cl), n = 2 (X = Cl, Br, I; Y = Cl, Br (only for X = I)) calculated on the B3LYP(D3BJ)/def2-TZVPP (MP2/def2-TZVPP) level of theory. Bond distances are given in pm. For structures with colored annotations, the central atom of the structure is a chloride (green), bromide (red) or iodide (violet). Bond length marked with an asterisk belong to non-minimum structures.

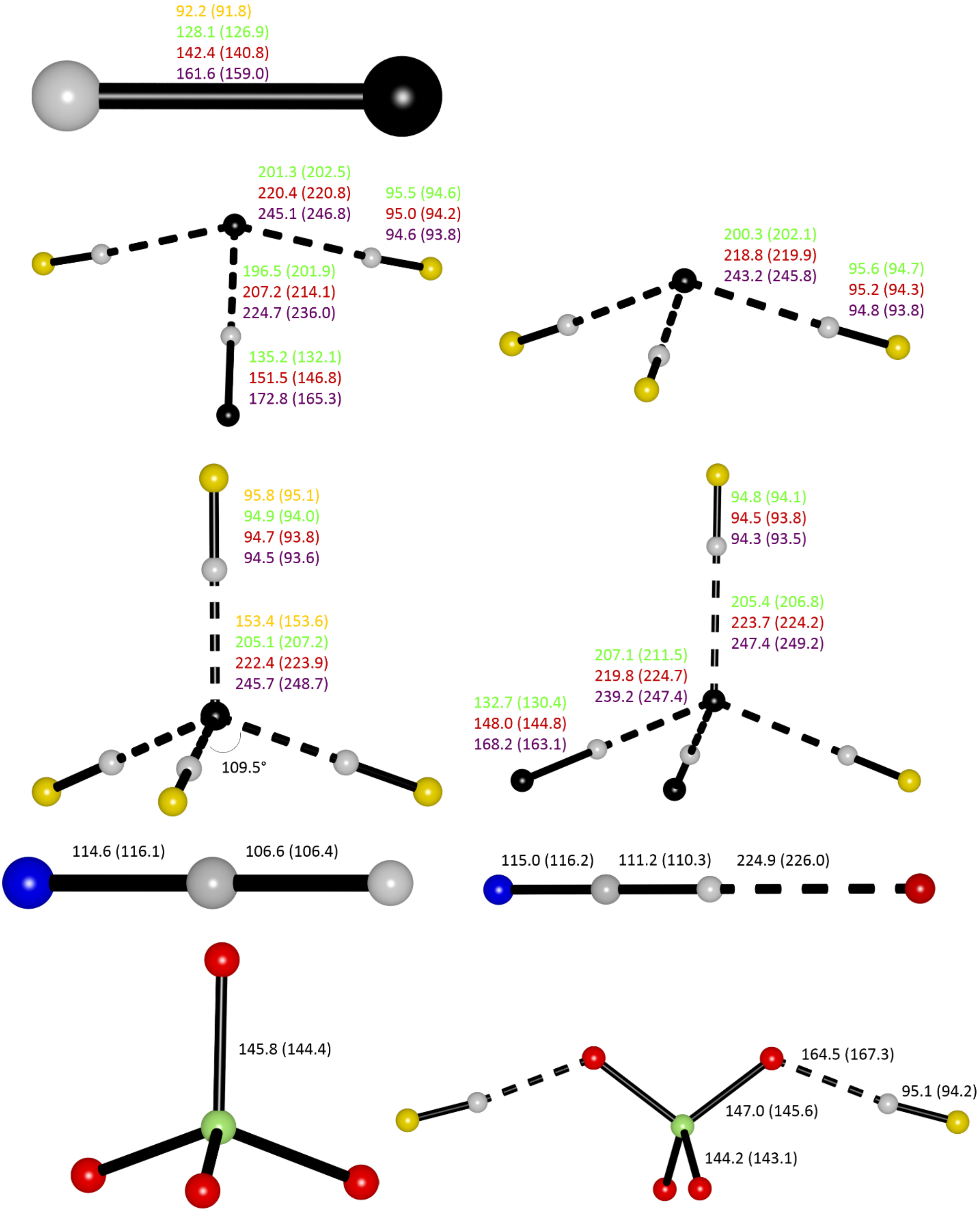

Figure S 22. Optimized structures of HX (X = F, Cl, Br, I, CN), $[X(HY)_n]^-$ (*n* = 1 (X = Br; Y = CN), *n* = 2 (X = ClO_4; Y = F)) and $[X(HF)_2(HX)_n]^-$ (*n* = 1,2; X = Cl, Br, I) calculated on the B3LYP(D3BJ)/def2-TZVPP (MP2/def2-TZVPP) level of theory. Bond distances are given in pm. For structures with colored annotations, the black atoms are equal to chlorine (green), bromine (red) or iodine (violet).

d) Calculated Energies and Free Reaction Energies

d1) B3LYP(D3BJ)/def2-TZVPP Energies

All Free Enthalpy calculations were carried out for T = 298.15 K and p = 1.0 bar if not stated otherwise.

Table S 3. Calculated energies on the B3LYP(D3BJ)/def2-TZVPP level of theory.

Verbindung	E_{tot} [E_H]	E_{tot} [kJ/mol^{-1}]	G (kJ mol^{-1})
F^-	–99.83878290	–262126.72	–262163.89
Cl^-	–460.22009153	–1208307.85	–1208347.34
Br^-	–2574.12130509	–6758355.49	–6758398.00
I^-	–297.79901555	–781871.32	–781915.54
$[ClHCl]^-$	–921.03498878	–2418177.36	–2418221.40
$[Br(HCl)]^-$	–3034.92480079	–7968195.06	–7968242.33
$[I(HCl)]^-$	–758.59431186	–1991689.37	–1991738.45
$[Cl(HCl)_2]^-$	–1381.83086281	–3627996.93	–3628038.71
$[Br(HCl)_2]^-$	–3495.71844848	–9178008.79	–9178054.78
$[I(HCl)_2]^-$	–1219.38440676	–3201493.76	–3201543.55
$[Cl(HCl)_3]^-$	–1842.62129282	–4837802.20	–4837839.27
$[Br(HCl)_3]^-$	–3956.50712263	–10387809.45	–10387852.55
$[I(HCl)_3]^-$	–1680.17097613	–4411288.90	–4411337.29
$[Cl(HCl)_4]^-$ (T_d)	–2303.40766790	–6047596.83	–6047628.85
$[Br(HCl)_4]^-$ (T_d)	–4417.29227743	–11597600.87	–11597640.15
$[I(HCl)_4]^-$ (T_d)	–2140.95474183	–5621076.67	–5621122.09
$[Cl(HCl)_4]^-$ (C_{4v})	–2303.40537183	–6047590.80	–
$[Br(HCl)_4]^-$ (C_{4v})	–4417.29053744	–11597596.31	–
$[I(HCl)_4]^-$ (C_{4v})	–2140.95342236	–5621073.21	–
$[Cl(HCl)_4]^-$ (D_{4h})	–2303.40521150	–6047590.38	–
$[Br(HCl)_4]^-$ (D_{4h})	–4417.29018688	–11597595.39	–
$[I(HCl)_4]^-$ (D_{4h})	–2140.95285991	–5621071.73	–

Verbindung	E_{tot} [E_H]	E_{tot} [kJ/mol^{-1}]	G (kJ mol^{-1})
HF	–100.45529924	–263745.39	–263764.34
HCl	–460.76800069	–1209746.39	–1209775.98
HBr	–2574.64739822	–6759736.74	–6759771.82
HI	–298.30894626	–783210.14	–783249.49
$[Cl(HF)_3]^-$	–761.69417796	–1999828.06	–1999828.94
$[Br(HF)_3]^-$	–2875.57908480	–7549832.89	–7549840.04
$[I(HF)_3]^-$	–599.24170540	–1573309.10	–1573321.72
$[F(HF)_4]^-$	–501.86273987	–1317640.62	–1317602.35
$[Cl(HF)_4]^-$	–862.17285192	–2263634.82	–2263617.09
$[Br(HF)_4]^-$	–2976.05574034	–7813634.35	–7813623.94
$[I(HF)_4]^-$	–699.71613583	–1837104.71	–1837101.53
$[Cl(HF)_2(HCl)]^-$	–1122.00327912	–2945819.61	–2945835.14
$[Br(HF)_2(HBr)]^-$	–5349.76873758	–14045817.82	–14045844.97
$[I(HF)_2(HI)]^-$	–797.09272783	–2092766.96	–2092803.93
$[Cl(HF)_2(HCl)_2]^-$	–1582.79017767	–4155615.61	–4155626.89
$[Br(HF)_2(HBr)_2]^-$	–7924.43294153	–20805598.69	–20805628.50
$[I(HF)_2(HI)_2]^-$	–1095.41551949	–2876013.45	–2876059.60
HCN	–93.41448394	–245259.73	–245268.06
$[Br(HCN)]^-$	–2667.57045814	–7003706.24	–7003731.25
$[ClO_4]^-$	–760.88102990	–1997693.14	–1997717.60
$[ClO_4(HF)_2]^-$	–961.84615092	–2525327.07	–2525321.74
$[I(HBr)_2]^-$	–5447.14613091	–14301482.17	–14301543.31

d2) SCS-MP2/def2-TZVPP Energies

Table S 4. Calculated energies on the SCS-MP2/def2-TZVPP level of theory.

Verbindung	E_{tot} [E_H]	E_{MP2} [E_H]	$E_{tot+MP2}$ [kJ/mol^{-1}]	G (kJ mol^{-1})
F^-	–99.44317906	–0.28553667	–261837.74	–261874.91
Cl^-	–459.55562243	–0.34621209	–1207472.27	–1207511.75
Br^-	–2572.48103042	–0.3269252	–6754907.29	–6754949.80
I^-	–296.74067393	–0.52332097	–780466.62	–780510.85
$[ClHCl]^-$	–919.68258054	–0.71488027	–2416503.53	–2416548.15
$[Br(HCl)]^-$	–3032.60126224	–0.69261723	–7963913.08	–7963958.39
$[I(HCl)]^-$	–756.85498183	–0.88706869	–1989451.75	–1989498.66
$[Cl(HCl)_2]^-$	–1379.80304795	–1.07440499	–3625493.75	–3625532.33
$[Br(HCl)_2]^-$	–3492.71796406	–1.05412848	–9172898.63	–9172941.03
$[I(HCl)_2]^-$	–1216.96744012	–1.24878262	–3198426.69	–3198472.71
$[Cl(HCl)_3]^-$	–1839.91936951	–1.43369196	–4834472.46	–4834505.44
$[Br(HCl)_3]^-$	–3952.83175140	–1.41445618	–10381873.42	–10381911.75
$[I(HCl)_3]^-$	–1677.07819556	–1.60943753	–4407394.38	–4407437.56
$[Cl(HCl)_4]^-$ (T_d)	–2300.03278462	–1.79243470	–6043442.11	–6043466.68
$[Br(HCl)_4]^-$ (T_d)	–4412.94331240	–1.77412492	–11590840.63	–11590872.65
$[I(HCl)_4]^-$ (T_d)	–2137.18750295	–1.96940329	–5616356.46	–5616394.51
$[Cl(HCl)_4]^-$ (C_{4v})	–2300.03034246	–1.79274818	–6043436.52	–
$[Br(HCl)_4]^-$ (C_{4v})	–4412.94147674	–1.77454505	–11590836.92	–
$[I(HCl)_4]^-$ (C_{4v})	–2137.18601756	–1.96956624	–5616352.99	–
$[Cl(HCl)_4]^-$ (D_{4h})	–2300.03039555	–1.79269401	–6043436.52	–
$[Br(HCl)_4]^-$ (D_{4h})	–4412.94147688	–1.77454523	–11590836.92	–11590864.57
$[I(HCl)_4]^-$ (D_{4h})	–2137.18623150	–1.96929017	–5616352.82	–5616396.26
HF	–100.06531033	–0.28555788	–263471.20	–263489.53
HCl	–460.09919729	–0.35546237	–1208923.71	–1208952.42
HBr	–2573.00161502	–0.33803472	–6756303.25	–6756337.53
HI	–297.24261157	–0.53552182	–781816.49	–781854.83

Verbindung	E_{tot} [E_H]	E_{MP2} [E_H]	$E_{tot+MP2}$ [kJ/mol^{-1}]	G (kJ mol^{-1})
$[Cl(HF)_3]^-$	–759.83733362	–1.21746339	–1998149.37	–1998145.70
$[Br(HF)_3]^-$	–2872.74826668	–1.19844224	–7545547.08	–7545550.13
$[I(HF)_3]^-$	–596.99304707	–1.39365936	–1571064.30	–1571071.78
$[F(HF)_4]^-$	–499.88269487	–1.44276685	–1316230.00	–1316187.06
$[Cl(HF)_4]^-$	–859.92224572	–1.50526207	–2261677.92	–2261655.64
$[Br(HF)_4]^-$	–2972.83074047	–1.48706458	–7809071.40	–7809055.51
$[I(HF)_4]^-$	–697.07269585	–1.68251934	–1834581.82	–1834577.63
$[Cl(HF)_2(HCl)]^-$	–1119.86466933	–1.28949297	–2943590.25	–2943603.66
$[Br(HF)_2(HBr)]^-$	–5345.67730279	–1.25411993	–14038368.45	–
$[I(HF)_2(HI)]^-$	–794.16045856	–1.64842598	–2089396.23	–2089430.07
$[Cl(HF)_2(HCl)_2]^-$	–1579.97762209	–1.64863075	–4152559.73	–4152564.22
$[Br(HF)_2(HBr)_2]^-$	–7918.68950195	–1.59711763	–20794712.52	–20794735.56
$[I(HF)_2(HI)_2]^-$	–1091.40906448	–2.18995181	–2871244.22	–2871284.31
HCN	–92.91031160	–0.38185792	–244938.59	–244947.72
$[Br(HCN)]^-$	–2665.42020359	–0.71296727	–6999932.64	–6999955.88
$[ClO_4]^-$	–758.83986267	–1.36504250	–1995917.98	–1995939.28
$[ClO_4(HF)_2]^-$	–959.01336844	–1.94300522	–2522990.96	–2522979.41
$[I(HBr)_2]^-$	–5442.77003718	–1.21681424	–14293187.48	–14293245.47

d3) SCS-MP2(COSMO)/def2-TZVPP Energies

Table S 5. Calculated energies on the SCS-MP2/def2-TZVPP level of theory including a COSMO solvent model (ε_r = 83.6).

Verbindung	$E_{tot+MP2+Cosmo}$ [E_H]	G [kJ mol^{-1}]	$G^{220\ K}$ [kJ mol^{-1}]
HF	–100.35975152	–263463.48	–263500.3976
HCl	–460.45952135	–1208735.39	–1208951.353
HBr	–2573.34382115	–6755062.13	–6756333.602
HI	–297.78122862	–781714.29	–781847.3757
$[F(HF)_4]^-$	–501.40579715	–1316154.36	–1316374.78
$[Cl(HF)_4]^-$	–861.50159252	–2261429.29	–2261825.281
$[Br(HF)_4]^-$	–2974.39012800	–7807767.83	–7809219.091
$[I(HF)_4]^-$	–698.82498418	–1834412.56	–1834723.916
F^-	–99.87812886	–262217.26	–262216.13
Cl^-	–460.02518246	–1207605.59	–1207604.46
Br^-	–2572.92321899	–6753965.96	–6753964.83
I^-	–297.36910044	–780638.12	–780636.99

d4) Free Reaction Energy Calculation

Table S 6. ΔE and ΔG for the reaction of $[X(HCl)_n]^-$ + HCl → $[X(HCl)_{n+1}]^-$ calculated on the B3LYP(D3BJ)/def2-TZVPP and SCS-MP2/def2-TZVPP level of theory.

X	n	ΔE_{B3LYP} [kJ mol^{-1}]	ΔG_{B3LYP} [kJ mol^{-1}]	ΔE_{MP2} [kJ mol^{-1}]	ΔG_{MP2} [kJ mol^{-1}]
Cl	1	–73.18	–41.33	–66.51	–31.76
Cl	2	–58.89	–24.59	–55.00	–20.69
Cl	3	–48.24	–13.60	–45.94	–8.82
Br	1	–67.34	–36.47	–61.84	–30.22
Br	2	–54.28	–21.80	–51.08	–18.30
Br	3	–45.04	–11.63	–43.50	–8.48
I	1	–58.01	–29.13	–51.23	–21.63
I	2	–48.75	–17.76	–43.98	–12.43
I	3	–41.39	–8.83	–38.37	–4.53

Table S 7. ΔE and ΔG for the reaction of $[X(HF)_4]^-$ + HF → $[F(HF)_4]^-$ + HX calculated on the B3LYP(D3BJ)/def2-TZVPP and SCS-MP2/def2-TZVPP level of theory.

X	ΔE_{B3LYP} [kJ mol^{-1}]	ΔG_{B3LYP} [kJ mol^{-1}]	ΔE_{MP2} [kJ mol^{-1}]	ΔG_{MP2} [kJ mol^{-1}]
Cl	–6.80	3.10	–4.58	5.70
Br	2.37	14.10	9.35	20.45
I	–0.66	14.03	6.53	25.27

Table S 8. ΔE, ΔG (298 K and 220 K) and K_{eq} (298 K and 220 K) for the reaction of $[X(HF)_4]^-$ + HF → $[F(HF)_4]^-$ + HX calculated on the SCS-MP2(COSMO)/def2-TZVPP level of theory.

X	$\Delta E_{COSMO,MP2}$ [kJ mol^{-1}]	$\Delta G^{298K}_{COSMO,MP2}$ [kJ mol^{-1}]	K_{eq} (298 K)	$\Delta G^{220K}_{COSMO,MP2}$ [kJ mol^{-1}]	K_{eq} (220 K)
Cl	–10.43	3.02	0.2959	–0.45	1.2824
Br	0.69	14.82	0.0025	11.11	0.0023
I	–6.01	7.40	0.0505	2.16	0.3074

Table S 9. ΔE and ΔG for the reaction of X^- + HF → F^- + HX calculated on the B3LYP(D3BJ)/def2-TZVPP and SCS-MP2/def–TZVPP level of theory.

X	ΔE_{B3LYP} [kJ mol^{-1}]	ΔG_{B3LYP} [kJ mol^{-1}]	ΔE_{MP2} [kJ mol^{-1}]	ΔG_{MP2} [kJ mol^{-1}]
Cl	180.13	171.81	182.02	173.96
Br	237.41	226.62	237.50	226.89
I	279.84	266.50	283.59	270.64

Table S 10. ΔE, ΔG (298 K and 220 K) and K_{eq} (298 K and 220 K) for the reaction of X^- + HF → F^- + HX calculated on the SCS-MP2(COSMO)/def2-TZVPP level of theory.

X	$\Delta E_{COSMO,MP2}$ [kJ mol^{-1}]	$\Delta G^{298K}_{COSMO,MP2}$ [kJ mol^{-1}]	K_{eq} (298 K)	$\Delta G^{220K}_{COSMO,MP2}$ [kJ mol^{-1}]	K_{eq} (220 K)
Cl	124,14	116,44	3,88E-21	117,45	1,295E-28
Br	160,21	150,08	4,92E-27	152,02	8,026E-37
I	182,46	170,09	1,53E-30	172,63	1,026E-41

Table S 11. ΔE and ΔG for the reaction of X^- + 4 HF → $[X(HF)_4]^-$ calculated on the B3LYP(D3BJ)/def2-TZVPP and SCS-MP2/def-TZVPP level of theory.

X	ΔE_{B3LYP} [kJ mol^{-1}]	ΔG_{B3LYP} [kJ mol^{-1}]	ΔE_{MP2} [kJ mol^{-1}]	ΔG_{MP2} [kJ mol^{-1}]
F	–532.35	–381.11	–507.44	–354.01
Cl	–345.42	–212.40	–320.84	–185.75
Br	–297.31	–168.59	–279.29	–147.57
I	–251.85	–128.64	–230.38	–108.64

Table S 12. ΔE and ΔG for the reaction of $[X(HF)_4]^-$ + 2 HX → $[X(HF)_2(HX)_2]^-$ + 2 HF calculated on the B3LYP(D3BJ)/def2-TZVPP and SCS-MP2/def-TZVPP level of theory.

X	ΔE_{B3LYP} [kJ mol^{-1}]	ΔG_{B3LYP} [kJ mol^{-1}]	ΔE_{MP2} [kJ mol^{-1}]	ΔG_{MP2} [kJ mol^{-1}]
Cl	–21.21	–13.48	–23.20	–17.19
Br	–18.37	–10.41	–22.97	–15.94
I	–20.77	–12.24	–28.17	–23.91

Table S 13. ΔE and ΔG for the reaction of $[X(HF)_3]^-$ + HX → $[X(HF)_2(HX)]^-$ + HF calculated on the B3LYP(D3BJ)/def2-TZVPP and SCS-MP2/def–TZVPP level of theory.

X	ΔE_{B3LYP} [kJ mol^{-1}]	ΔG_{B3LYP} [kJ mol^{-1}]	ΔE_{MP2} [kJ mol^{-1}]	ΔG_{MP2} [kJ mol^{-1}]
Cl	–9.45	–5.44	–11.62	–4.92
Br	–6.42	–2.55	–10.68	–
I	–6.89	–2.94	–13.36	–7.01

Table S 14. ΔE and ΔG for the reaction of $[Br(HCl)]^-$ + HCN → $[Br(HCN)]^-$ + 2 HCl calculated on B3LYP(D3BJ)/def2-TZVPP and SCS-MP2/def-TZVPP level of theory.

ΔE_{B3LYP} [kJ mol^{-1}]	ΔG_{B3LYP} [kJ mol^{-1}]	ΔE_{MP2} [kJ mol^{-1}]	ΔG_{MP2} [kJ mol^{-1}]
2.17	3.17	–4.68	–2.19

Table S 15. ΔE and ΔG for the reaction of $[ClO_4]^-$ + 2 HF → $[ClO_4(HF)_2]^-$ calculated on B3LYP(D3BJ)/def2-TZVPP and SCS-MP2/def–TZVPP level of theory.

ΔE_{B3LYP} [kJ mol^{-1}]	ΔG_{B3LYP} [kJ mol^{-1}]	ΔE_{MP2} [kJ mol^{-1}]	ΔG_{MP2} [kJ mol^{-1}]
–143.15	–75.46	–130.57	–61.06

$$X^- + HF \rightarrow F^- + HX$$

$$K_{eq} = \frac{c(F^-) * c(HX)}{c(X^-) * c(HF)}$$

$$c_0(X^-) = c_0(HF);\ c_0(F^-) = c_0(HX) = 0$$

$$K_{eq} = \frac{c^2(HX)}{c^2(X^-)}$$

$$\sqrt{K_{eq}} = \frac{c(HX)}{c(X^-)}$$

Scheme S 1. Calculation of the ratio of $c(HX)/c(X^-)$ from the K_{eq}.

d5) Comparison of Different Geometries for the $[X(HCl)_4]^-$ Anion

Table S 16. Comparison of different geometries for the $[X(HCl)_4]^-$ anion calculated on the B3LYP(D3BJ)/def2-TZVPP and SCS-MP2/def–TZVPP level of theory.

X	Structure	ΔE_{B3LYP}	ΔG_{B3LYP}	ΔE_{MP2}	ΔG_{B3LYP}
Cl	T_d	0.00	0.00	0.00	0.00
Cl	C_{4v}	6.03	-	5.59	-
Cl	D_{4h}	6.45	-	5.59	-
Br	T_d	0.00	0.00	0.00	0.00
Br	C_{4v}	4.57	-	3.72	-
Br	D_{4h}	5.49	-	3.72	8.09
I	T_d	0.00	0.00	0.00	0.00
I	C_{4v}	3.46	-	3.47	-
I	D_{4h}	4.94	-	3.64	–1.75

e) Coordinates of Optimized Structures

HF

B3LYP(D3BJ)/def2-TZVPP

H 0.000000 0.000000 –0.461179

F 0.000000 0.000000 0.461179

SCS-MP2/def2-TZVPP

H 0.000000 0.000000 –0.458911

F 0.000000 0.000000 0.458911

SCS-MP2(Cosmo)/def2-TZVPP

H 0.000000 0.000000 –0.461744

F 0.000000 0.000000 0.461744

HCl

B3LYP(D3BJ)/def2-TZVPP

Cl 0.000000 0.000000 –0.640328

H 0.000000 0.000000 0.640328

SCS-MP2/def2-TZVPP

Cl 0.000000 0.000000 –0.634481

H 0.000000 0.000000 0.634481

SCS-MP2(Cosmo)/def2-TZVPP

Cl 0.000000 0.000000 –0.636892

H 0.000000 0.000000 0.636892

HBr

B3LYP(D3BJ)/def2-TZVPP

Br 0.000000 0.000000 –0.712233

H 0.000000 0.000000 0.712233

SCS-MP2/def2-TZVPP

Br 0.000000 0.000000 –0.704014

H 0.000000 0.000000 0.704014

SCS-MP2(Cosmo)/def2-TZVPP

Br 0.000000 0.000000 –0.705872

H 0.000000 0.000000 0.705872

HI

B3LYP(D3BJ)/def2-TZVPP

I 0.000000 0.000000 –0.808006

H 0.000000 0.000000 0.808006

SCS-MP2/def2-TZVPP

I 0.000000 0.000000 –0.795165

H 0.000000 0.000000 0.795165

SCS-MP2(Cosmo)/def2-TZVPP

I 0.000000 0.000000 –0.796356

H 0.000000 0.000000 0.796356

[ClHCl]⁻

B3LYP(D3BJ)/def2-TZVPP

H	0.000000	0.000000	0.000000
Cl	0.000000	0.000000	1.573967
Cl	0.000000	0.000000	–1.573967

SCS-MP2/def2-TZVPP

H	0.000000	0.000000	0.000000
Cl	0.000000	0.000000	1.553706
Cl	0.000000	0.000000	–1.553706

[Br(HCl)]⁻

B3LYP(D3BJ)/def2-TZVPP

Br	0.000000	0.000000	–1.772801
H	0.000000	0.000000	0.177039
Cl	0.000000	0.000000	1.595761

SCS-MP2/def2-TZVPP

Br	0.000000	0.000000	–1.790977
H	0.000000	0.000000	0.211286
Cl	0.000000	0.000000	1.579691

$[I(HCl)]^-$

B3LYP(D3BJ)/def2-TZVPP

I	0.000000	0.000000	–1.981020
H	0.000000	0.000000	0.305121
Cl	0.000000	0.000000	1.675899

SCS-MP2/def2-TZVPP

I	0.000000	0.000000	–2.009612
H	0.000000	0.000000	0.340109
Cl	0.000000	0.000000	1.669504

$[Cl(HCl)_2]^-$

B3LYP(D3BJ)/def2-TZVPP

Cl	0.000000	0.000000	–1.189832
H	1.519875	0.000000	–0.087011
Cl	2.666892	0.000000	0.681932
H	–1.519875	0.000000	–0.087011
Cl	–2.666892	0.000000	0.681932

SCS-MP2/def2-TZVPP

Cl	0.000000	0.000000	–1.167151
H	1.577917	0.000000	–0.075525
Cl	2.705578	0.000000	0.659105
H	–1.577917	0.000000	–0.075525
Cl	–2.705578	0.000000	0.659105

[Br(HCl)$_2$]$^–$

B3LYP(D3BJ)/def2-TZVPP

Br 0.000000 0.000000 –1.325421

H 1.670105 0.000000 –0.061451

Cl 2.781437 0.000000 0.724181

H –1.670105 0.000000 –0.061451

Cl –2.781437 0.000000 0.724181

SCS-MP2/def2-TZVPP

Br 0.000000 0.000000 –1.291113

H 1.736878 0.000000 –0.051867

Cl 2.834406 0.000000 0.697444

H –1.736878 0.000000 –0.051867

Cl –2.834406 0.000000 0.697444

[I(HCl)$_2$]$^–$

B3LYP(D3BJ)/def2-TZVPP

I 0.000000 0.000000 –1.477137

H 1.869464 0.000000 –0.026730

Cl 2.957217 0.000000 0.765467

H –1.869464 0.000000 –0.026730

Cl –2.957217 0.000000 0.765467

SCS-MP2/def2-TZVPP

I 0.000000 0.000000 –1.459194

H 1.943319 0.000000 –0.017041

Cl 3.012660 0.000000 0.746805

H –1.943319 0.000000 –0.017041

Cl –3.012660 0.000000 0.746805

$[Cl(HCl)_3]^-$

B3LYP(D3BJ)/def2-TZVPP

Cl	0.000000	0.000000	–0.879072
H	–0.919089	1.591909	–0.103460
H	–0.919089	–1.591909	–0.103460
H	1.838178	0.000000	–0.103460
Cl	–1.545523	2.676924	0.384014
Cl	–1.545523	–2.676924	0.384014
Cl	3.091046	0.000000	0.384014

SCS-MP2/def2-TZVPP

Cl	0.000000	0.000000	–0.725716
H	–0.962451	1.667013	–0.082150
H	–0.962451	–1.667013	–0.082150
H	1.924901	0.000000	–0.082150
Cl	–1.591766	2.757019	0.311586
Cl	–1.591766	–2.757019	0.311586
Cl	3.183531	0.000000	0.311586

$[Br(HCl)_3]^-$

B3LYP(D3BJ)/def2-TZVPP

Br	0.000000	0.000000	–0.947131
H	–0.999944	1.731953	–0.075508
H	–0.999944	–1.731953	–0.075508
H	1.999887	0.000000	–0.075508
Cl	–1.621384	2.808319	0.417815
Cl	–1.621384	–2.808319	0.417815
Cl	3.242768	0.000000	0.417815

SCS-MP2/def2-TZVPP

Br	0.000000	0.000000	–0.759237
H	–1.049633	1.818017	–0.056990
H	–1.049633	–1.818017	–0.056990
H	2.099265	0.000000	–0.056990
Cl	–1.675478	2.902013	0.336665
Cl	–1.675478	–2.902013	0.336665
Cl	3.350956	0.000000	0.336665

$[I(HCl)_3]^-$

B3LYP(D3BJ)/def2-TZVPP

I	0.000000	0.000000	–1.094932
H	–1.102429	1.909463	–0.079987
H	–1.102429	–1.909463	–0.079987
H	2.204858	0.000000	–0.079987
Cl	–1.716120	2.972407	0.434200
Cl	–1.716120	–2.972407	0.434200
Cl	3.432240	0.000000	0.434200

SCS-MP2/def2-TZVPP

I	0.000000	0.000000	–1.954157
H	–0.747144	1.294092	–0.182502
H	–0.747144	–1.294092	–0.182502
H	1.494289	0.000000	–0.182502
Cl	–1.165476	2.018664	0.823123
Cl	–1.165476	–2.018664	0.823123
Cl	2.330953	0.000000	0.823123

[Cl(HCl)$_4$]$^-$ (T_d)

B3LYP(D3BJ)/def2-TZVPP

Cl	0.000000	0.000000	0.000000
H	–1.197810	1.197810	1.197810
H	1.197810	–1.197810	1.197810
H	1.197810	1.197810	–1.197810
H	–1.197810	–1.197810	–1.197810
Cl	–1.963477	1.963477	1.963477
Cl	1.963477	–1.963477	1.963477
Cl	–1.963477	–1.963477	–1.963477
Cl	1.963477	1.963477	–1.963477

SCS-MP2/def2-TZVPP

Cl	0.000000	0.000000	0.000000
H	–1.216419	1.216419	1.216419
H	1.216419	–1.216419	1.216419
H	1.216419	1.216419	–1.216419
H	–1.216419	–1.216419	–1.216419
Cl	–1.970050	1.970050	1.970050
Cl	1.970050	–1.970050	1.970050
Cl	–1.970050	–1.970050	–1.970050
Cl	1.970050	1.970050	–1.970050

[Br(HCl)$_4$]$^-$ (T_d)

B3LYP(D3BJ)/def2-TZVPP

Br	0.000000	0.000000	0.000000
H	–1.296774	1.296774	1.296774
H	1.296774	–1.296774	1.296774
H	1.296774	1.296774	–1.296774
H	–1.296774	–1.296774	–1.296774
Cl	–2.060803	2.060803	2.060803
Cl	2.060803	–2.060803	2.060803
Cl	–2.060803	–2.060803	–2.060803
Cl	2.060803	2.060803	–2.060803

SCS-MP2/def2-TZVPP

Br	0.000000	0.000000	0.000000
H	–1.313284	1.313284	1.313284
H	1.313284	–1.313284	1.313284
H	1.313284	1.313284	–1.313284
H	–1.313284	–1.313284	–1.313284
Cl	–2.065142	2.065142	2.065142
Cl	2.065142	–2.065142	2.065142
Cl	–2.065142	–2.065142	–2.065142
Cl	2.065142	2.065142	–2.065142

[I(HCl)$_4$]$^-$ (T_d)

B3LYP(D3BJ)/def2-TZVPP

I 0.000000 0.000000 0.000000
H –1.429803 1.429803 1.429803
H 1.429803 –1.429803 1.429803
H 1.429803 1.429803 –1.429803
H –1.429803 –1.429803 –1.429803
Cl –2.192207 2.192207 2.192207
Cl 2.192207 –2.192207 2.192207
Cl –2.192207 –2.192207 –2.192207
Cl 2.192207 2.192207 –2.192207SCS-MP2

SCS-MP2/def2-TZVPP

I 0.000000 0.000000 0.000000
H –1.454771 1.454771 1.454771
H 1.454771 –1.454771 1.454771
H 1.454771 1.454771 –1.454771
H –1.454771 –1.454771 –1.454771
Cl –2.204221 2.204221 2.204221
Cl 2.204221 –2.204221 2.204221
Cl –2.204221 –2.204221 –2.204221
Cl 2.204221 2.204221 –2.204221

[Cl(HCl)$_4$]$^–$ (C_{4v})

B3LYP(D3BJ)/def2-TZVPP

Cl 2.343520 –2.343520 0.209007

Cl 2.343520 2.343520 0.209007

Cl –2.343520 2.343520 0.209007

Cl –2.343520 –2.343520 0.209007

H 1.430237 –1.430237 –0.078022

H 1.430237 1.430237 –0.078022

H –1.430237 1.430237 –0.078022

H –1.430237 –1.430237 –0.078022

Cl 0.000000 0.000000 –0.596460

SCS-MP2/def2-TZVPP

I 0.000000 0.000000 –0.858598

Cl –2.580905 2.580905 0.291250

Cl –2.580905 –2.580905 0.291250

Cl 2.580905 –2.580905 0.291250

H –1.701759 1.701759 –0.076619

H –1.701759 –1.701759 –0.076619

H 1.701759 –1.701759 –0.076619

H 1.701759 1.701759 –0.076619

Cl 2.580905 2.580905 0.291250

[Br(HCl)$_4$]$^-$ (C_{4v})

B3LYP(D3BJ)/def2-TZVPP

Br	0.000000	0.000000	–0.895160
Cl	–2.381303	2.381303	0.322298
Cl	–2.381303	–2.381303	0.322298
Cl	2.381303	–2.381303	0.322298
H	–1.496408	1.496408	–0.098527
H	–1.496408	–1.496408	–0.098527
H	1.496408	–1.496408	–0.098527
H	1.496408	1.496408	–0.098527
Cl	2.381303	2.381303	0.322298

SCS-MP2/def2-TZVPP

Br	0.000000	0.000000	–0.014293
Cl	–2.535096	2.535096	0.005359
Cl	–2.535096	–2.535096	0.005359
Cl	2.535096	–2.535096	0.005359
H	–1.615808	1.615808	–0.001804
H	–1.615808	–1.615808	–0.001804
H	1.615808	–1.615808	–0.001804
H	1.615808	1.615808	–0.001804
Cl	2.535096	2.535096	0.005359

[I(HCl)$_4$]$^-$ (C_{4v})

B3LYP(D3BJ)/def2-TZVPP

I	0.000000	0.000000	–1.175025
Cl	–2.450390	2.450390	0.404631
Cl	–2.450390	–2.450390	0.404631
Cl	2.450390	–2.450390	0.404631
H	–1.592900	1.592900	–0.110894
H	–1.592900	–1.592900	–0.110894
H	1.592900	–1.592900	–0.110894
H	1.592900	1.592900	–0.110894
Cl	2.450390	2.450390	0.404631

SCS-MP2/def2-TZVPP

I	0.000000	0.000000	–1.696015
Cl	–1.995239	1.995239	0.621679
Cl	–1.995239	–1.995239	0.621679
Cl	1.995239	–1.995239	0.621679
H	–1.280962	1.280962	–0.197694
H	–1.280962	–1.280962	–0.197694
H	1.280962	–1.280962	–0.197694
H	1.280962	1.280962	–0.197694
Cl	1.995239	1.995239	0.621679

$[Cl(HCl)_4]^-$ (D_{4h})

B3LYP(D3BJ)/def2-TZVPP

Cl	0.000000	0.000000	0.000000
H	–1.475152	–1.475152	0.000000
H	–1.475152	1.475152	0.000000
H	1.475152	1.475152	0.000000
H	1.475152	–1.475152	0.000000
Cl	2.410450	–2.410450	0.000000
Cl	2.410450	2.410450	0.000000
Cl	–2.410450	2.410450	0.000000
Cl	–2.410450	–2.410450	0.000000

SCS-MP2/def2-TZVPP

Cl	0.000000	0.000000	0.000000
H	–1.497088	–1.497088	0.000000
H	–1.497088	1.497088	0.000000
H	1.497088	1.497088	0.000000
H	1.497088	–1.497088	0.000000
Cl	2.418555	–2.418555	0.000000
Cl	2.418555	2.418555	0.000000
Cl	–2.418555	2.418555	0.000000
Cl	–2.418555	–2.418555	0.000000

[Br(HCl)$_4$]$^-$ (D_{4h})

B3LYP(D3BJ)/def2-TZVPP

Br	0.000000	0.000000	0.000000
H	–1.597153	–1.597153	0.000000
H	–1.597153	1.597153	0.000000
H	1.597153	1.597153	0.000000
H	1.597153	–1.597153	0.000000
Cl	2.530408	–2.530408	0.000000
Cl	2.530408	2.530408	0.000000
Cl	–2.530408	2.530408	0.000000
Cl	–2.530408	–2.530408	0.000000

SCS-MP2/def2-TZVPP

Br	0.000000	0.000000	0.000000
H	–1.615832	–1.615832	0.000000
H	–1.615832	1.615832	0.000000
H	1.615832	1.615832	0.000000
H	1.615832	–1.615832	0.000000
Cl	2.535133	–2.535133	0.000000
Cl	2.535133	2.535133	0.000000
Cl	–2.535133	2.535133	0.000000
Cl	–2.535133	–2.535133	0.000000

$[I(HCl)_4]^-$ (D_{4h})

B3LYP(D3BJ)/def2-TZVPP

I	0.000000	0.000000	0.000000
H	–1.760388	–1.760388	0.000000
H	–1.760388	1.760388	0.000000
H	1.760388	1.760388	0.000000
H	1.760388	–1.760388	0.000000
Cl	2.691606	–2.691606	0.000000
Cl	2.691606	2.691606	0.000000
Cl	–2.691606	2.691606	0.000000
Cl	–2.691606	–2.691606	0.000000

SCS-MP2/def2-TZVPP

I	0.000000	0.000000	0.000000
H	–1.790623	–1.790623	0.000000
H	–1.790623	1.790623	0.000000
H	1.790623	1.790623	0.000000
H	1.790623	–1.790623	0.000000
Cl	2.707147	–2.707147	0.000000
Cl	2.707147	2.707147	0.000000
Cl	–2.707147	2.707147	0.000000
Cl	–2.707147	–2.707147	0.000000

$[Cl(HF)_3]^-$

B3LYP(D3BJ)/def2-TZVPP

Cl	0.000000	0.000000	–0.720767
H	–0.940389	1.628802	–0.030464
H	–0.940389	–1.628802	–0.030464
H	1.880779	0.000000	–0.030464
F	–1.394358	2.415099	0.270722
F	–1.394358	–2.415099	0.270722
F	2.788716	0.000000	0.270722

SCS-MP2/def2-TZVPP

Cl	0.000000	0.000000	–0.659658
H	–0.959930	1.662647	–0.027505
H	–0.959930	–1.662647	–0.027505
H	1.919860	0.000000	–0.027505
F	–1.413022	2.447426	0.247393
F	–1.413022	–2.447426	0.247393
F	2.826044	0.000000	0.247393

$[Br(HF)_3]^-$

B3LYP(D3BJ)/def2-TZVPP

Br	0.000000	0.000000	–0.799669
H	–1.022128	1.770378	–0.019595
H	–1.022128	–1.770378	–0.019595
H	2.044256	0.000000	–0.019595
F	–1.473093	2.551472	0.286154
F	–1.473093	–2.551472	0.286154
F	2.946186	0.000000	0.286154

SCS-MP2/def2-TZVPP

Br	0.000000	0.000000	–0.717639
H	–1.042639	1.805904	–0.018553
H	–1.042639	–1.805904	–0.018553
H	2.085279	0.000000	–0.018553
F	–1.493501	2.586820	0.257769
F	–1.493501	–2.586820	0.257769
F	2.987003	0.000000	0.257769

$[I(HF)_3]^-$

B3LYP(D3BJ)/def2-TZVPP

I	0.000000	0.000000	–0.957356
H	–1.119553	1.939122	–0.007921
H	–1.119553	–1.939122	–0.007921
H	2.239106	0.000000	–0.007921
F	–1.563212	2.707563	0.327042
F	–1.563212	–2.707563	0.327042
F	3.126425	0.000000	0.327042

SCS-MP2/def2-TZVPP

I	0.000000	0.000000	–0.927171
H	–1.140239	1.974952	–0.008867
H	–1.140239	–1.974952	–0.008867
H	2.280479	0.000000	–0.008867
F	–1.580235	2.737048	0.317926
F	–1.580235	–2.737048	0.317926
F	3.160471	0.000000	0.317926

[F(HF)$_4$]$^{–}$

B3LYP(D3BJ)/def2-TZVPP

F	0.000000	0.000000	0.000000
H	–0.885406	0.885406	0.885406
H	0.885406	–0.885406	0.885406
H	0.885406	0.885406	–0.885406
H	–0.885406	–0.885406	–0.885406
F	–1.438722	1.438722	1.438722
F	1.438722	–1.438722	1.438722
F	–1.438722	–1.438722	–1.438722
F	1.438722	1.438722	–1.438722

SCS-MP2/def2-TZVPP

F	0.000000	0.000000	0.000000
H	–0.886007	0.886007	0.886007
H	0.886007	–0.886007	0.886007
H	0.886007	0.886007	–0.886007
H	–0.886007	–0.886007	–0.886007
F	–1.435248	1.435248	1.435248
F	1.435248	–1.435248	1.435248
F	–1.435248	–1.435248	–1.435248
F	1.435248	1.435248	–1.435248

SCS-MP2(Cosmo)/def2-TZVPP

F	0.000000	0.000000	0.000000
H	–0.871966	0.871966	0.871966
H	0.871966	–0.871966	0.871966
H	0.871966	0.871966	–0.871966
H	–0.871966	–0.871966	–0.871966
F	–1.421939	1.421939	1.421939
F	1.421939	–1.421939	1.421939
F	–1.421939	–1.421939	–1.421939
F	1.421939	1.421939	–1.421939

[Cl(HF)$_4$]$^-$

B3LYP(D3BJ)/def2-TZVPP

Cl	0.000000	0.000000	0.000000
H	–1.183878	1.183878	1.183878
H	1.183878	–1.183878	1.183878
H	1.183878	1.183878	–1.183878
H	–1.183878	–1.183878	–1.183878
F	–1.731777	1.731777	1.731777
F	1.731777	–1.731777	1.731777
F	–1.731777	–1.731777	–1.731777
F	1.731777	1.731777	–1.731777

SCS-MP2/def2-TZVPP

Cl	0.000000	0.000000	0.000000
H	–1.196327	1.196327	1.196327
H	1.196327	–1.196327	1.196327
H	1.196327	1.196327	–1.196327
H	–1.196327	–1.196327	–1.196327
F	–1.739316	1.739316	1.739316
F	1.739316	–1.739316	1.739316
F	–1.739316	–1.739316	–1.739316
F	1.739316	1.739316	–1.739316

SCS-MP2(Cosmo)/def2-TZVPP

Cl	0.000000	0.000000	0.000000
H	–1.177918	1.177918	1.177918
H	1.177918	–1.177918	1.177918
H	1.177918	1.177918	–1.177918
H	–1.177918	–1.177918	–1.177918
F	–1.721341	1.721341	1.721341
F	1.721341	–1.721341	1.721341
F	–1.721341	–1.721341	–1.721341
F	1.721341	1.721341	–1.721341

$[Br(HF)_4]^-$

B3LYP(D3BJ)/def2-TZVPP

Br	0.000000	0.000000	0.000000
H	–1.284047	1.284047	1.284047
H	1.284047	–1.284047	1.284047
H	1.284047	1.284047	–1.284047
H	–1.284047	–1.284047	–1.284047
F	–1.830661	1.830661	1.830661
F	1.830661	–1.830661	1.830661
F	–1.830661	–1.830661	–1.830661
F	1.830661	1.830661	–1.830661

SCS-MP2/def2-TZVPP

Br	0.000000	0.000000	0.000000
H	–1.292553	1.292553	1.292553
H	1.292553	–1.292553	1.292553
H	1.292553	1.292553	–1.292553
H	–1.292553	–1.292553	–1.292553
F	–1.834306	1.834306	1.834306
F	1.834306	–1.834306	1.834306
F	–1.834306	–1.834306	–1.834306
F	1.834306	1.834306	–1.834306

SCS-MP2(Cosmo)/def2-TZVPP

Br	0.000000	0.000000	0.000000
H	–1.273450	1.273450	1.273450
H	1.273450	–1.273450	1.273450
H	1.273450	1.273450	–1.273450
H	–1.273450	–1.273450	–1.273450
F	–1.815565	1.815565	1.815565
F	1.815565	–1.815565	1.815565
F	–1.815565	–1.815565	–1.815565
F	1.815565	1.815565	–1.815565

$[I(HF)_4]^–$

B3LYP(D3BJ)/def2-TZVPP

I	0.000000	0.000000	0.000000
H	–1.418277	1.418277	1.418277
H	1.418277	–1.418277	1.418277
H	1.418277	1.418277	–1.418277
H	–1.418277	–1.418277	–1.418277
F	–1.963592	1.963592	1.963592
F	1.963592	–1.963592	1.963592
F	–1.963592	–1.963592	–1.963592
F	1.963592	1.963592	–1.963592

SCS-MP2/def2-TZVPP

I	0.000000	0.000000	0.000000
H	–1.435630	1.435630	1.435630
H	1.435630	–1.435630	1.435630
H	1.435630	1.435630	–1.435630
H	–1.435630	–1.435630	–1.435630
F	–1.975744	1.975744	1.975744
F	1.975744	–1.975744	1.975744
F	–1.975744	–1.975744	–1.975744
F	1.975744	1.975744	–1.975744

SCS-MP2(Cosmo)/def2-TZVPP

I	0.000000	0.000000	0.000000
H	–1.413038	1.413038	1.413038
H	1.413038	–1.413038	1.413038
H	1.413038	1.413038	–1.413038
H	–1.413038	–1.413038	–1.413038
F	–1.953566	1.953566	1.953566
F	1.953566	–1.953566	1.953566
F	–1.953566	–1.953566	–1.953566
F	1.953566	1.953566	–1.953566

$[Cl(HF)_2(HCl)]^-$

B3LYP(D3BJ)/def2-TZVPP

Cl	–0.603826	–0.476276	0.000000
H	1.142484	–1.377394	0.000000
H	–0.684001	0.698976	1.631766
H	–0.684001	0.698976	–1.631766
Cl	2.327111	–2.029983	0.000000
F	–0.748873	1.242994	–2.414068
F	–0.748873	1.242994	2.414068

SCS-MP2/def2-TZVPP

Cl	–0.526306	–0.402665	0.000000
H	1.215126	–1.424323	0.000000
H	–0.706374	0.728021	1.670581
H	–0.706374	0.728021	–1.670581
Cl	2.340112	–2.116489	0.000000
F	–0.808081	1.243861	–2.457551
F	–0.808081	1.243861	2.457551

$[Br(HF)_2(HBr)]^-$

B3LYP(D3BJ)/def2-TZVPP

Br	–0.686766	–0.551129	0.000000
H	1.177838	–1.455566	0.000000
H	–0.726065	0.764258	1.768316
H	–0.726065	0.764258	–1.768316
Br	2.521607	–2.155385	0.000000
F	–0.780265	1.316925	–2.539056
F	–0.780265	1.316925	2.539056

SCS-MP2/def2-TZVPP

Br	–0.567480	–0.435697	0.000000
H	1.281867	–1.513991	0.000000
H	–0.759827	0.800865	1.819516
H	–0.759827	0.800865	–1.819516
Br	2.533362	–2.282132	0.000000
F	–0.864036	1.315188	–2.601834
F	–0.864036	1.315188	2.601834

$[I(HF)_2(HI)]^-$

B3LYP(D3BJ)/def2-TZVPP

I	–0.794966	–0.631007	0.000000
H	1.245197	–1.573076	0.000000
H	–0.788879	0.857573	1.947342
H	–0.788879	0.857573	–1.947342
I	2.790506	–2.346627	0.000000
F	–0.831479	1.417925	–2.708239
F	–0.831479	1.417925	2.708239

SCS-MP2/def2-TZVPP

I	–0.733837	–0.564453	0.000000
H	1.361298	–1.650223	0.000000
H	–0.828860	0.894000	1.988542
H	–0.828860	0.894000	–1.988542
I	2.811635	–2.443970	0.000000
F	–0.890678	1.435466	–2.751708
F	–0.890678	1.435466	2.751708

$[Cl(HF)_2(HCl)_2]^-$

B3LYP(D3BJ)/def2-TZVPP

Cl	0.000000	0.000000	0.069724
H	0.000000	–1.684662	1.244022
H	0.000000	1.684662	1.244022
H	–1.679324	0.000000	–1.142979
H	1.679324	0.000000	–1.142979
F	0.000000	–2.463116	1.786024
F	0.000000	2.463116	1.786024
Cl	2.754443	0.000000	–1.921247
Cl	–2.754443	0.000000	–1.921247

SCS-MP2/def2-TZVPP

Cl	0.000000	0.000000	0.058175
H	0.000000	–1.685960	1.256328
H	0.000000	1.685960	1.256328
H	–1.726910	0.000000	–1.162551
H	1.726910	0.000000	–1.162551
F	0.000000	–2.455018	1.798515
F	0.000000	2.455018	1.798515
Cl	2.788182	0.000000	–1.920697
Cl	–2.788182	0.000000	–1.920697

[Br(HF)$_2$(HBr)$_2$]$^-$

B3LYP(D3BJ)/def2-TZVPP

Br	0.000000	0.000000	0.092030
H	0.000000	–1.842430	1.360038
H	0.000000	1.842430	1.360038
H	–1.767674	0.000000	–1.214027
H	1.767674	0.000000	–1.214027
F	0.000000	–2.617982	1.900232
F	0.000000	2.617982	1.900232
Br	2.959508	0.000000	–2.091576
Br	–2.959508	0.000000	–2.091576

SCS-MP2/def2-TZVPP

Br	0.000000	0.000000	0.070083
H	0.000000	–1.827120	1.369936
H	0.000000	1.827120	1.369936
H	–1.830186	0.000000	–1.234317
H	1.830186	0.000000	–1.234317
F	0.000000	–2.593499	1.910884
F	0.000000	2.593499	1.910884
Br	3.003923	0.000000	–2.080862
Br	–3.003923	0.000000	–2.080862

$[I(HF)_2(HI)_2]^-$

B3LYP(D3BJ)/def2-TZVPP

I 0.000000 0.000000 0.106405

H 0.000000 –2.034170 1.515033

H 0.000000 2.034170 1.515033

H –1.927296 0.000000 –1.310731

H 1.927296 0.000000 –1.310731

F 0.000000 –2.807844 2.053495

F 0.000000 2.807844 2.053495

I 3.280115 0.000000 –2.310317

I –3.280115 0.000000 –2.310317

SCS-MP2/def2-TZVPP

I 0.000000 0.000000 0.096166

H 0.000000 –2.037437 1.530367

H 0.000000 2.037437 1.530367

H –2.012879 0.000000 –1.342672

H 2.012879 0.000000 –1.342672

F 0.000000 –2.804479 2.065453

F 0.000000 2.804479 2.065453

I 3.332435 0.000000 –2.300549

I –3.332435 0.000000 –2.300549

HCN

B3LYP(D3BJ)/def2-TZVPP

N	0.000000	0.000000	–1.119691
C	0.000000	0.000000	0.026672
H	0.000000	0.000000	1.093019

SCS-MP2/def2-TZVPP

N	0.000000	0.000000	–1.128806
C	0.000000	0.000000	0.032387
H	0.000000	0.000000	1.096419

[Br(HCN)]$^{-}$

B3LYP(D3BJ)/def2-TZVPP

N	0.000000	0.000000	–1.981358
C	0.000000	0.000000	–0.830885
H	0.000000	0.000000	0.281576
Br	0.000000	0.000000	2.530666

SCS-MP2/def2-TZVPP

N	0.000000	0.000000	–1.989153
C	0.000000	0.000000	–0.825273
H	0.000000	0.000000	0.277276
Br	0.000000	0.000000	2.537150

$[ClO_4]^–$

B3LYP(D3BJ)/def2-TZVPP

Cl	0.000000	0.000000	0.000000
O	–0.841533	–0.841533	–0.841533
O	0.841533	0.841533	–0.841533
O	0.841533	–0.841533	0.841533
O	–0.841533	0.841533	0.841533

SCS-MP2/def2-TZVPP

Cl	0.000000	0.000000	0.000000
O	–0.833959	–0.833959	–0.833959
O	0.833959	0.833959	–0.833959
O	0.833959	–0.833959	0.833959
O	–0.833959	0.833959	0.833959

$[ClO_4(HF)_2]^–$

B3LYP(D3BJ)/def2-TZVPP

Cl	0.000000	0.000000	–0.152634
O	–1.188516	0.000000	–0.968721
O	0.000000	1.176691	0.729168
H	0.000000	2.714659	0.146034
F	0.000000	3.636671	–0.088983
O	1.188516	0.000000	–0.968721
O	0.000000	–1.176691	0.729168
H	0.000000	–2.714659	0.146034
F	0.000000	–3.636671	–0.088983

SCS-MP2/def2-TZVPP

Cl	0.000000	0.000000	–0.149360
O	–1.178670	0.000000	–0.960470
O	0.000000	1.167777	0.719627
H	0.000000	2.736501	0.138995
F	0.000000	3.652335	–0.082289
O	1.178670	0.000000	–0.960470
O	0.000000	–1.167777	0.719627
H	0.000000	–2.736501	0.138995
F	0.000000	–3.652335	–0.082289

$[I(HBr)_2]^–$

B3LYP(D3BJ)/def2-TZVPP

I	0.000000	0.000000	1.486489
H	–1.790372	0.000000	0.078445
Br	–3.013908	0.000000	–0.820384
H	1.790372	0.000000	0.078445
Br	3.013908	0.000000	–0.820384

SCS-MP2/def2-TZVPP

I	0.000000	0.000000	1.450730
H	–1.890524	0.000000	0.059807
Br	–3.094313	0.000000	–0.783867
H	1.890524	0.000000	0.059807
Br	3.094313	0.000000	–0.783867

f) Calculated Vibrational Spectra

Table S 17. HF.

B3LYP(D3BJ)/def2-TZVPP				SCS-MP2/def2-TZVPP			
Nr.	Symmetry	Wavenumber [cm^{-1}]	IR intensity [km mol^{-1}]	Nr.	Symmetry	Wavenumber [cm^{-1}]	IR intensity [km mol^{-1}]
1	Σ^+	4081.2	106	1	Σ^+	4146.9	111

Table S 18. HCl.

B3LYP(D3BJ)/def2-TZVPP				SCS-MP2/def2-TZVPP			
Nr.	Symmetry	Wavenumber [cm^{-1}]	IR intensity [km mol^{-1}]	Nr.	Symmetry	Wavenumber [cm^{-1}]	IR intensity [km mol^{-1}]
1	Σ^+	2949.0	35	1	Σ^+	3062.9	40

Table S 19. HBr.

B3LYP(D3BJ)/def2-TZVPP				SCS-MP2/def2-TZVPP			
Nr.	Symmetry	Wavenumber [cm^{-1}]	IR intensity [km mol^{-1}]	Nr.	Symmetry	Wavenumber [cm^{-1}]	IR intensity [km mol^{-1}]
1	Σ^+	2618.4	9	1	Σ^+	2719.9	10

Table S 20. HI.

B3LYP(D3BJ)/def2-TZVPP				SCS-MP2/def2-TZVPP			
Nr.	Symmetry	Wavenumber [cm^{-1}]	IR intensity [km mol–1]	Nr.	Symmetry	Wavenumber [cm^{-1}]	IR intensity [km mol–1]
1	Σ^+	2291.9	1	1	Σ^+	2427.4	1

Table S 21. Computed vibrational frequencies calculated on SCS-MP2(Cosmo)/def2-TZVPP (ε_r=83.6).

HF				HCl			
Nr.	Symmetry	Wavenumber [cm^{-1}]	IR intensity [km mol–1]	Nr.	Symmetry	Wavenumber [cm^{-1}]	IR intensity [km mol–1]
1	Σ^+	4053.3	223	1	Σ^+	3017.3	116
HBr				HI			
Nr.	Symmetry	Wavenumber [cm^{-1}]	IR intensity [km mol–1]	Nr.	Symmetry	Wavenumber [cm^{-1}]	IR intensity [km mol–1]
1	Σ^+	2691.0	47	1	Σ^+	2412.6	4

Table S 22. $[ClHCl]^–$.

B3LYP(D3BJ)/def2-TZVPP				SCS-MP2/def2-TZVPP			
Nr.	Symmetry	Wavenumber [cm–1]	IR intensity [km mol–1]	Nr.	Symmetry	Wavenumber [cm–1]	IR intensity [km mol–1]
1	Σ_g^+	324.5	0	1	Σ_g^+	342.5	0
2	Σ_u^-	744.0	5192	2	Σ_u^-	492.9	6365
3	Π_u	834.3	13	3	Π_u	892.0	16
4	Π_u	834.3	13	4	Π_u	892.0	16

Table S 23. $[Br(HCl)]^–$.

B3LYP(D3BJ)/def2-TZVPP				SCS-MP2/def2-TZVPP			
Nr.	Symmetry	Wavenumber [cm–1]	IR intensity [km mol–1]	Nr.	Symmetry	Wavenumber [cm–1]	IR intensity [km mol–1]
1	Σ^+	183.6	53	1	Σ^+	165.6	55
2	Π	717.5	8	2	Π	725.8	12
3	Π	717.5	8	3	Π	725.8	12
4	Σ^+	1484.3	4504	4	Σ^+	1810.2	4392

Table S 24. $[I(HCl)]^–$.

B3LYP(D3BJ)/def2-TZVPP				SCS-MP2/def2-TZVPP			
Nr.	Symmetry	Wavenumber [cm–1]	IR intensity [km mol–1]	Nr.	Symmetry	Wavenumber [cm–1]	IR intensity [km mol–1]
1	Σ^+	135.1	20	1	Σ^+	123.5	18
2	Π	604.4	7	2	Π	607.0	10
3	Π	604.4	7	3	Π	607.0	10
4	Σ^+	1893.0	4061	4	Σ^+	2262.3	3282

Table S 25. $[Cl(HCl)_2]^–$.

B3LYP(D3BJ)/def2-TZVPP				SCS-MP2/def2-TZVPP			
Nr.	Symmetry	Wavenumber [$cm^{–1}$]	IR intensity [km $mol^{–1}$]	Nr.	Symmetry	Wavenumber [$cm^{–1}$]	IR intensity [km $mol^{–1}$]
1	A_1	23.3	0	1	A_1	21.3	0
2	A_1	194.8	23	2	A_1	181.4	25
3	B_1	198.4	168	3	B_1	182.5	156
4	B_1	677.2	0	4	B_1	693.6	2
5	B_2	689.4	30	5	B_2	706.0	38
6	A_2	697.1	0	6	A_2	710.7	0
7	A_1	752.1	16	7	A_1	747.1	21
8	B_1	1683.0	5745	8	B_1	1965.9	5412
9	A_1	1912.2	1683	9	A_1	2156.4	1545

Table S 26. $[Br(HCl)_2]^-$.

B3LYP(D3BJ)/def2-TZVPP				SCS-MP2/def2-TZVPP			
Nr.	Symmetry	Wavenumber [cm^{-1}]	IR intensity [km mol^{-1}]	Nr.	Symmetry	Wavenumber [cm^{-1}]	IR intensity [km mol^{-1}]
1	A_1	17.0	0	1	A_1	16.4	0
2	B_1	149.4	51	2	A_1	139.6	11
3	A_1	149.5	11	3	B_1	141.5	47
4	B_1	608.5	0	4	B_1	621.8	3
5	B_2	618.3	22	5	B_2	636.3	28
6	A_2	626.0	0	6	A_2	640.6	0
7	A_1	665.0	12	7	A_1	661.5	15
8	B_1	1923.9	5030	8	B_1	2214.9	4462
9	A_1	2064.6	1801	9	A_1	2324.2	1520

Table S 27. $[I(HCl)_2]^-$.

B3LYP(D3BJ)/def2-TZVPP				SCS-MP2/def2-TZVPP			
Nr.	Symmetry	Wavenumber [cm^{-1}]	IR intensity [km mol^{-1}]	Nr.	Symmetry	Wavenumber [cm^{-1}]	IR intensity [km mol^{-1}]
1	A_1	12.9	0.1	1	A_1	12.3	0
2	A_1	121.5	7	2	A_1	111.8	6
3	B_1	122.7	22	3	B_1	114.2	19
4	B_1	537.0	0.3	4	B_1	546.9	3
5	B_2	544.9	16	5	B_2	557.8	23
6	A_2	551.6	0	6	A_2	561.6	0
7	A_1	580.9	10	7	A_1	576.0	13
8	B_1	2104.2	4588	8	B_1	2426.6	3550
9	A_1	2186.6	1942	9	A_1	2484.1	1462

Table S 28. $[Cl(HCl)_3]^-$.

B3LYP(D3BJ)/def2-TZVPP				SCS-MP2/def2-TZVPP			
Nr.	Symmetry	Wavenumber [cm^{-1}]	IR intensity [km mol^{-1}]	Nr.	Symmetry	Wavenumber [cm^{-1}]	IR intensity [km mol^{-1}]
1	*E*	17.5	0	1	A_1	16.1	0
2	*E*	17.5	0	2	*E*	17.0	0
3	A_1	18.9	0	3	*E*	17.0	0
4	A_1	160.1	6	4	A_1	144.5	4
5	*E*	173.9	69	5	*E*	166.7	68
6	*E*	173.9	69	6	*E*	166.7	68
7	A_2	585.8	0	7	A_2	603.0	0
8	*E*	594.3	7	8	*E*	612.8	8
9	*E*	594.3	7	9	*E*	612.8	8
10	A_1	630.5	39	10	A_1	632.5	51
11	*E*	649.9	11	11	*E*	652.1	15
12	*E*	649.9	11	12	*E*	652.1	15
13	*E*	2102.3	3774	13	*E*	2346.8	3406
14	*E*	2102.3	3774	14	*E*	2346.8	3406
15	A_1	2289.9	632	15	A_1	2496.0	393

Table S 29. $[Br(HCl)_3]^-$.

B3LYP(D3BJ)/def2-TZVPP				SCS-MP2/def2-TZVPP			
Nr.	Symmetry	Wavenumber [cm^{-1}]	IR intensity [km mol^{-1}]	Nr.	Symmetry	Wavenumber [cm^{-1}]	IR intensity [km mol^{-1}]
1	A_1	13.5	0	1	A_1	11.1	0
2	*E*	13.9	0	2	*E*	13.9	0
3	*E*	13.9	0	3	*E*	13.9	0
4	A_1	130.0	3	4	A_1	120.5	2
5	*E*	133.9	28	5	*E*	130.4	27
6	*E*	133.9	28	6	*E*	130.4	27
7	A_2	541.5	0	7	A_2	563.1	0
8	*E*	546.8	4	8	*E*	571.3	6
9	*E*	546.8	4	9	*E*	571.3	6
10	A_1	576.2	30	10	A_1	585.0	39
11	*E*	593.0	7	11	*E*	601.3	9
12	*E*	593.0	7	12	*E*	601.3	9
13	*E*	2197.5	3609	13	*E*	2450.5	3133
14	*E*	2197.5	3609	14	*E*	2450.5	3133
15	A_1	2331.0	724	15	A_1	2552.4	407

Table S 30. $[I(HCl)_3]^-$.

B3LYP(D3BJ)/def2-TZVPP				SCS-MP2/def2-TZVPP			
Nr.	Symmetry	Wavenumber [cm^{-1}]	IR intensity [km mol^{-1}]	Nr.	Symmetry	Wavenumber [cm^{-1}]	IR intensity [km mol^{-1}]
1	A_1	10.9	0	1	A_1	10.2	0
2	E	11.4	0	2	E	11.6	0
3	E	11.4	0	3	E	11.6	0
4	A_1	111.2	2	4	A_1	101.7	2
5	E	113.1	14	5	E	107.1	13
6	E	113.1	14	6	E	107.1	13
7	A_2	491.0	0	7	A_2	503.5	0
8	E	493.9	2	8	E	507.9	6
9	E	493.9	2	9	E	507.9	6
10	A_1	519.9	23	10	A_1	522.1	31
11	E	533.3	5	11	E	534.0	7
12	E	533.3	5	12	E	534.0	7
13	E	2278.0	3524	13	E	2558.6	2704
14	E	2278.0	3524	14	E	2558.6	2704
15	A_1	2368.3	906	15	A_1	2620.7	557

Table S 31. $[Cl(HCl)_4]^-$ (T_d).

B3LYP(D3BJ)/def2-TZVPP				SCS-MP2/def2-TZVPP			
Nr.	Symmetry	Wavenumber [cm^{-1}]	IR intensity [km mol^{-1}]	Nr.	Symmetry	Wavenumber [cm^{-1}]	IR intensity [km mol^{-1}]
1	E	12.7	0	1	E	13.2	0
2	E	12.7	0	2	E	13.2	0
3	T_2	15.1	0	3	T_2	17.0	0
4	T_2	15.1	0	4	T_2	17.0	0
5	T_2	15.1	0	5	T_2	17.0	0
6	A_1	122.4	0	6	A_1	116.7	0
7	T_2	159.7	41	7	T_2	153.6	36
8	T_2	159.7	41	8	T_2	153.6	36
9	T_2	159.7	41	9	T_2	153.6	36
10	T_1	521.8	0	10	T_1	543.1	0
11	T_1	521.8	0	11	T_1	543.1	0
12	T_1	521.8	0	12	T_1	543.1	0
13	T_2	562.5	34	13	T_2	579.3	45
14	T_2	562.5	34	14	T_2	579.3	45
15	T_2	562.5	34	15	T_2	579.3	45
16	E	580.4	0	16	E	589.8	0
17	E	580.4	0	17	E	589.8	0
18	T_2	2326.2	2846	18	T_2	2541.6	2356
19	T_2	2326.2	2846	19	T_2	2541.6	2356
20	T_2	2326.2	2846	20	T_2	2541.6	2356
21	A_1	2487.8	0	21	A_1	2668.3	0

Table S 32. $[Br(HCl)_4]^-$ (T_d).

B3LYP(D3BJ)/def2-TZVPP				SCS-MP2/def2-TZVPP			
Nr.	Symmetry	Wavenumber [cm^{-1}]	IR intensity [km mol^{-1}]	Nr.	Symmetry	Wavenumber [cm^{-1}]	IR intensity [km mol^{-1}]
1	E	11.1	0	1	E	11.4	0
2	E	11.1	0	2	E	11.4	0
3	T_2	11.7	0	3	T_2	11.7	0
4	T_2	11.7	0	4	T_2	11.7	0
5	T_2	11.7	0	5	T_2	11.7	0
6	A_1	110.0	0	6	A_1	105.4	0
7	T_2	124.2	19	7	T_2	120.9	16
8	T_2	124.2	19	8	T_2	120.9	16
9	T_2	124.2	19	9	T_2	120.9	16
10	T_1	490.6	0	10	T_1	519.2	0
11	T_1	490.6	0	11	T_1	519.2	0
12	T_1	490.6	0	12	T_1	519.2	0
13	T_2	519.9	21	13	T_2	544.9	31
14	T_2	519.9	21	14	T_2	544.9	31
15	T_2	519.9	21	15	T_2	544.9	31
16	E	540.9	0	16	E	556.0	0
17	E	540.9	0	17	E	556.0	0
18	T_2	2367.5	2867	18	T_2	2591.5	2286
19	T_2	2367.5	2867	19	T_2	2591.5	2286
20	T_2	2367.5	2867	20	T_2	2591.5	2286
21	A_1	2492.2	0	21	A_1	2685.5	0

Table S 33. $[I(HCl)_4]^-$ (T_d).

B3LYP(D3BJ)/def2-TZVPP				SCS-MP2/def2-TZVPP			
Nr.	Symmetry	Wavenumber [cm^{-1}]	IR intensity [km mol^{-1}]	Nr.	Symmetry	Wavenumber [cm^{-1}]	IR intensity [km mol^{-1}]
1	T_2	10.0	0	1	E	10.0	0
2	T_2	10.0	0	2	E	10.0	0
3	T_2	10.0	0	3	T_2	10.8	0
4	E	10.0	0	4	T_2	10.8	0
5	E	10.0	0	5	T_2	10.8	0
6	A_1	98.5	0	6	A_1	91.3	0
7	T_2	106.6	11	7	T_2	100.9	9
8	T_2	106.6	11	8	T_2	100.9	9
9	T_2	106.6	11	9	T_2	100.9	9
10	T_1	452.2	0	10	T_1	469.5	0
11	T_1	452.2	0	11	T_1	469.5	0
12	T_1	452.2	0	12	T_1	469.5	0
13	T_2	474.9	14	13	T_2	487.8	25
14	T_2	474.9	14	14	T_2	487.8	25
15	T_2	474.9	14	15	T_2	487.8	25
16	E	496.3	0	16	E	500.0	0
17	E	496.3	0	17	E	500.0	0
18	T_2	2402.4	2985	18	T_2	2651.8	2167
19	T_2	2402.4	2985	19	T_2	2651.8	2167
20	T_2	2402.4	2985	20	T_2	2651.8	2167
21	A_1	2494.4	0	21	A_1	2714.8	0

Table S 34. $[Cl(HCl)_4]^-$ (C_{4v}).

B3LYP(D3BJ)/def2-TZVPP				SCS-MP2/def2-TZVPP			
Nr.	Symmetry	Wavenumber [cm–1]	IR intensity [km mol–1]	Nr.	Symmetry	Wavenumber [cm–1]	IR intensity [km mol–1]
1	B_2	–10.6	0	1	B_2	–10.3	0
2	A_1	15.7	0	2	A_1	10.5	0
3	E	18.9	0	3	E	19.1	0
4	E	18.9	0	4	E	19.1	0
5	B_1	22.1	0	5	B_1	21.9	0
6	B_2	99.7	0	6	B_2	96.5	0
7	A_1	131.9	1	7	A_1	117.2	0
8	E	170.4	72	8	E	166.5	71
9	E	170.4	72	9	E	166.5	71
10	B_2	489.8	0	10	B_2	512.2	0
11	A_2	492.6	0	11	A_2	516.6	0
12	E	518.7	14	12	E	546.7	18
13	E	518.7	14	13	E	546.7	18
14	E	539.6	35	14	A_1	550.4	75
15	E	539.6	35	15	E	553.1	34
16	A_1	539.6	58	16	E	553.1	34
17	B_1	604.7	0	17	B_1	611.1	0
18	E	2354.5	3929	18	E	2562.4	3451
19	E	2354.5	3929	19	E	2562.4	3451
20	B_2	2367.4	0	20	B_2	2580.6	0
21	A_1	2522.4	177	21	A_1	2697.6	26

Table S 35. $[Br(HCl)_4]^-$ (C_{4v}).

B3LYP(D3BJ)/def2-TZVPP				SCS-MP2/def2-TZVPP			
Nr.	Symmetry	Wavenumber [cm–1]	IR intensity [km mol–1]	Nr.	Symmetry	Wavenumber [cm–1]	IR intensity [km mol–1]
1	B_2	–7.1	0	1	B_2	–6.1	0
2	E	14.6	0	2	A_1	13.9	0
3	E	14.6	0	3	E	16.1	0
4	A_1	14.7	0	4	E	16.1	0
5	B_1	18.7	0	5	B_1	18.5	0
6	B_2	93.8	0	6	B_2	93.4	0
7	A_1	117.9	2	7	A_1	105.1	0
8	E	127.1	27	8	E	128.5	30
9	E	127.1	27	9	E	128.5	30
10	A_2	462.1	0	10	B_2	505.6	0
11	B_2	471.8	0	11	A_2	505.9	0
12	E	479.7	14	12	A_1	530.2	62
13	E	479.7	14	13	E	531.2	33
14	E	502.4	18	14	E	531.2	33
15	E	502.4	18	15	E	532.4	0
16	A_1	512.6	40	16	E	532.4	0
17	B_1	554.0	0	17	B_1	580.7	0
18	E	2397.9	3639	18	E	2612.9	3381
19	E	2397.9	3639	19	E	2612.9	3381
20	B_2	2400.2	0	20	B_2	2631.0	0
21	A_1	2527.9	433	21	A_1	2717.4	0

Table S 36. $[I(HCl)_4]^-$ (C_{4v}).

B3LYP(D3BJ)/def2-TZVPP				SCS-MP2/def2-TZVPP			
Nr.	Symmetry	Wavenumber [cm^{-1}]	IR intensity [km mol^{-1}]	Nr.	Symmetry	Wavenumber [cm^{-1}]	IR intensity [km mol^{-1}]
1	B_2	–3.0	0	1	B_2	–5.2	0
2	E	10.6	0	2	A_1	9.0	0
3	E	10.6	0	3	E	11.3	0
4	A_1	12.9	0	4	E	11.3	0
5	B_1	14.7	0	5	B_1	13.8	0
6	B_2	87.3	0	6	B_2	83.2	0
7	A_1	105.5	2	7	A_1	94.5	1
8	E	107.2	14	8	E	104.0	13
9	E	107.2	14	9	E	104.0	13
10	A_2	426.6	0	10	A_2	450.0	0
11	E	437.4	11	11	B_2	454.0	0
12	E	437.4	11	12	E	461.3	16
13	B_2	443.2	0	13	E	461.3	16
14	E	457.5	11	14	E	474.6	9
15	E	457.5	11	15	E	474.6	9
16	A_1	476.5	28	16	A_1	477.3	43
17	B_1	496.3	0	17	B_1	508.0	0
18	B_2	2428.3	0	18	E	2668.7	2859
19	E	2433.5	3487	19	E	2668.7	2859
20	E	2433.5	3487	20	B_2	2673.7	0
21	A_1	2530.6	778	21	A_1	2737.2	327

Table S 37. $[Cl(HCl)_4]^-$ (D_{4h}).

B3LYP(D3BJ)/def2-TZVPP				SCS-MP2/def2-TZVPP			
Nr.	Symmetry	Wavenumber [cm^{-1}]	IR intensity [km mol^{-1}]	Nr.	Symmetry	Wavenumber [cm^{-1}]	IR intensity [km mol^{-1}]
1	A_{2u}	–13.3	0	1	B_1u	–19,5	0
2	B_{1u}	–11.9	0	2	A_{2u}	17,8	0
3	E_u	16.4	0	3	E_u	19,5	0
4	E_u	16.4	0	4	E_u	19,5	0
5	B_{1g}	20.2	0	5	B_{1g}	21,9	0
6	B_{2g}	101.0	0	6	B_{2g}	96,6	0
7	A_{1g}	119.4	0	7	A_{1g}	114,7	0
8	E_u	173.9	80	8	E_u	167,2	72
9	E_u	173.9	80	9	E_u	167,2	72
10	B_{1u}	478.4	0	10	A_{2g}	516,7	0
11	A_{2g}	493.6	0	11	B_{1u}	527,6	0
12	A_{2u}	517.2	65	12	E_u	550,5	52
13	E_u	527.0	46	13	E_u	550,5	52
14	E_u	527.0	46	14	A_{2u}	563,6	78
15	E_g	528.6	0	15	E_g	564,4	0
16	E_g	528.6	0	16	E_g	564,4	0
17	B_{1g}	601.5	0	17	B_{1g}	609,5	0
18	E_u	2354.2	4242	18	E_u	2562,4	3491
19	E_u	2354.2	4242	19	E_u	2562,4	3491
20	B_{2g}	2381.1	0	20	B_{2g}	2582,2	0
21	A_{1g}	2525.6	0	21	A_{1g}	2698,0	0

Table S 38. $[Br(HCl)_4]^-$ (D_{4h}).

B3LYP(D3BJ)/def2-TZVPP				SCS-MP2/def2-TZVPP			
Nr.	Symmetry	Wavenumber [cm^{-1}]	IR intensity [km mol^{-1}]	Nr.	Symmetry	Wavenumber [cm^{-1}]	IR intensity [km mol^{-1}]
1	A_{2u}	–11.1	0	1	E_u	16.3	0
2	B_{1u}	–10.2	0	2	E_u	16.3	0
3	E_u	10.5	0	3	B_{1u}	18.5	0
4	E_u	10.5	0	4	B_{1g}	18.6	0
5	B_{1g}	15.9	0	5	A_{2u}	18.8	0
6	B_{2g}	95.2	0	6	B_{2g}	93.3	0
7	A_{1g}	107.0	0	7	A_{1g}	105.0	0
8	E_u	130.6	34	8	E_u	128.6	30
9	E_u	130.6	34	9	E_u	128.6	30
10	B_{1u}	452.5	0	10	A_{2g}	505.8	0
11	A_{2g}	465.1	0	11	B_{1u}	506.9	0
12	A_{2u}	476.2	52	12	E_u	531.1	33
13	E_u	483.2	28	13	E_u	531.1	33
14	E_u	483.2	28	14	A_{2u}	531.5	62
15	E_g	496.4	0	15	E_g	533.8	0
16	E_g	496.4	0	16	E_g	533.8	0
17	B_{1g}	551.1	0	17	B_{1g}	580.7	0
18	E_u	2397.6	4264	18	E_u	2612.9	3381
19	E_u	2354.2	4242	19	E_u	2612.9	3381
20	B_{2g}	2381.1	0	20	B_{2g}	2631.0	0
21	A_{1g}	2525.6	0	21	A_{1g}	2717.4	0

Table S 39. $[I(HCl)_4]^-$ (D_{4h}).

B3LYP(D3BJ)/def2-TZVPP				SCS-MP2/def2-TZVPP			
Nr.	Symmetry	Wavenumber [cm^{-1}]	IR intensity [km mol^{-1}]	Nr.	Symmetry	Wavenumber [cm^{-1}]	IR intensity [km mol^{-1}]
1	A_{2u}	–10.2	0	1	B_{1u}	1.0	0
2	B_{1u}	–9.5	0	2	A_{2u}	10.7	0
3	E_u	5.0	0	3	E_u	11.7	0
4	E_u	5.0	0	4	E_u	11.7	0
5	B_{1g}	12.4	0	5	B_{1g}	14.5	0
6	B_{2g}	88.2	0	6	B_{2g}	84.5	0
7	A_{1g}	95.9	0	7	A_{1g}	90.6	0
8	E_u	110.1	19	8	E_u	106.0	15
9	E_u	110.1	19	9	E_u	106.0	15
10	B_{1u}	413.9	0	10	A_{2g}	449.5	0
11	A_{2g}	428.7	0	11	B_{1u}	461.1	0
12	A_{2u}	428.9	42	12	E_u	461.6	24
13	E_u	434.5	18	13	E_u	461.6	24
14	E_u	434.5	18	14	A_{2u}	475.7	54
15	E_g	454.7	0	15	E_g	487.2	0
16	E_g	454.7	0	16	E_g	487.2	0
17	B_{1g}	494.6	0	17	B_{1g}	504.0	0
18	E_u	2434.2	4409	18	E_u	2672.8	3163
19	E_u	2434.2	4409	19	E_u	2672.8	3163
20	B_{2g}	2458.4	0	20	B_{2g}	2686.6	0
21	A_{1g}	2540.1	0	21	A_{1g}	2744.3	0

Table S 40. $[Cl(HF)_3]^-$.

B3LYP(D3BJ)/def2-TZVPP				SCS-MP2/def2-TZVPP			
Nr.	Symmetry	Wavenumber [cm^{-1}]	IR intensity [km mol^{-1}]	Nr.	Symmetry	Wavenumber [cm^{-1}]	IR intensity [km mol^{-1}]
1	A_1	23.7	0	1	A_1	24.2	0
2	*E*	27.1	0	2	*E*	27.7	0
3	*E*	27.1	0	3	*E*	27.7	0
4	A_1	199.8	1	4	A_1	191.2	2
5	*E*	231.1	18	5	*E*	223.6	20
6	*E*	231.1	18	6	*E*	223.6	20
7	A_2	712.4	0	7	A_2	743.0	0
8	*E*	728.0	46	8	*E*	758.1	47
9	*E*	728.0	46	9	*E*	758.1	47
10	A_1	764.5	228	10	A_1	786.2	259
11	*E*	788.9	73	11	*E*	809.4	87
12	*E*	788.9	73	12	*E*	809.4	87
13	*E*	3349.5	2284	13	*E*	3513.6	2100
14	*E*	3349.5	2284	14	*E*	3513.6	2100
15	A_1	3439.7	399	15	A_1	3594.0	308

Table S 41. $[Br(HF)_3]^-$.

B3LYP(D3BJ)/def2-TZVPP				SCS-MP2/def2-TZVPP			
Nr.	Symmetry	Wavenumber [cm^{-1}]	IR intensity [km mol^{-1}]	Nr.	Symmetry	Wavenumber [cm^{-1}]	IR intensity [km mol^{-1}]
1	A_1	18.8	2	1	A_1	16.5	1
2	*E*	23.0	1	2	*E*	22.8	1
3	*E*	23.0	1	3	*E*	22.8	1
4	A_1	168.6	1	4	A_1	163.8	1
5	*E*	181.0	7	5	*E*	177.4	8
6	*E*	181.0	7	6	*E*	177.4	8
7	A_2	654.6	0	7	A_2	683.0	0
8	*E*	664.8	43	8	*E*	694.5	44
9	*E*	664.8	43	9	*E*	694.5	44
10	A_1	694.3	196	10	A_1	715.3	222
11	*E*	718.2	56	11	*E*	739.3	67
12	*E*	718.2	56	12	*E*	739.3	67
13	*E*	3434.6	2193	13	*E*	3593.9	2005
14	*E*	3434.6	2193	14	*E*	3593.9	2005
15	A_1	3497.6	445	15	A_1	3649.7	326

Table S 42. $[I(HF)_3]^-$.

B3LYP(D3BJ)/def2-TZVPP				SCS-MP2/def2-TZVPP			
Nr.	Symmetry	Wavenumber [cm^{-1}]	IR intensity [$km\ mol^{-1}$]	Nr.	Symmetry	Wavenumber [cm^{-1}]	IR intensity [$km\ mol^{-1}$]
1	A_1	17.0	2	1	A_1	17.7	1
2	E	19.8	1	2	E	20.7	1
3	E	19.8	1	3	E	20.7	1
4	A_1	146.0	1	4	A_1	139.8	1
5	E	152.2	3	5	E	145.8	4
6	E	152.2	3	6	E	145.8	4
7	A_2	589.1	0	7	A_2	621.9	0
8	E	595.3	41	8	E	628.2	51
9	E	595.3	41	9	E	628.2	51
10	A_1	624.8	171	10	A_1	650.6	197
11	E	643.3	46	11	E	666.3	56
12	E	643.3	46	12	E	666.3	56
13	E	3511.4	2131	13	E	3690.7	1799
14	E	3511.4	2131	14	E	3690.7	1799
15	A_1	3552.8	587	15	A_1	3725.7	461

Table S 43. $[F(HF)_4]^-$.

B3LYP(D3BJ)/def2-TZVPP				SCS-MP2/def2-TZVPP			
Nr.	Symmetry	Wavenumber [cm^{-1}]	IR intensity [$km\ mol^{-1}$]	Nr.	Symmetry	Wavenumber [cm^{-1}]	IR intensity [$km\ mol^{-1}$]
1	E	39.4	0	1	E	38.1	0
2	E	39.4	0	2	E	38.1	0
3	T_2	55.4	2	3	T_2	55.7	1
4	T_2	55.4	2	4	T_2	55.7	1
5	T_2	55.4	2	5	T_2	55.7	1
6	A_1	247.7	0	6	A_1	240.7	0
7	T_2	310.8	26	7	T_2	299.2	28
8	T_2	310.8	26	8	T_2	299.2	28
9	T_2	310.8	26	9	T_2	299.2	28
10	T_1	800.1	0	10	T_1	838.0	0
11	T_1	800.1	0	11	T_1	838.0	0
12	T_1	800.1	0	12	T_1	838.0	0
13	E	946.8	0	13	E	970.7	0
14	E	946.8	0	14	E	970.7	0
15	T_2	955.9	335	15	T_2	989.1	348
16	T_2	955.9	335	16	T_2	989.1	348
17	T_2	955.9	335	17	T_2	989.1	348
18	T_2	3291.0	1857	18	T_2	3390.4	1830
19	T_2	3291.0	1857	19	T_2	3390.4	1830
20	T_2	3291.0	1857	20	T_2	3390.4	1830
21	A_1	3529.2	0	21	A_1	3618.2	0

Table S 44. $[Cl(HF)_4]^-$.

B3LYP(D3BJ)/def2-TZVPP				SCS-MP2/def2-TZVPP			
Nr.	Symmetry	Wavenumber [cm^{-1}]	IR intensity [km mol^{-1}]	Nr.	Symmetry	Wavenumber [cm^{-1}]	IR intensity [km mol^{-1}]
1	E	23.2	0	1	E	19.6	0
2	E	23.2	0	2	E	19.6	0
3	T_2	26.4	1	3	T_2	25.5	1
4	T_2	26.4	1	4	T_2	25.5	1
5	T_2	26.4	1	5	T_2	25.5	1
6	A_1	171.1	0	6	A_1	164.9	0
7	T_2	211.2	11	7	T_2	202.3	12
8	T_2	211.2	11	8	T_2	202.3	12
9	T_2	211.2	11	9	T_2	202.3	12
10	T_1	646.2	0	10	T_1	677.3	0
11	T_1	646.2	0	11	T_1	677.3	0
12	T_1	646.2	0	12	T_1	677.3	0
13	T_2	705.9	214	13	T_2	733.2	242
14	T_2	705.9	214	14	T_2	733.2	242
15	T_2	705.9	214	15	T_2	733.2	242
16	E	726.3	0	16	E	747.5	0
17	E	726.3	0	17	E	747.5	0
18	T_2	3499.2	1899	18	T_2	3648.7	1682
19	T_2	3499.2	1899	19	T_2	3648.7	1682
20	T_2	3499.2	1899	20	T_2	3648.7	1682
21	A_1	3593.3	0	21	A_1	3730.8	0

Table S 45. $[Br(HF)_4]^-$.

B3LYP(D3BJ)/def2-TZVPP				SCS-MP2/def2-TZVPP			
Nr.	Symmetry	Wavenumber [cm^{-1}]	IR intensity [km mol^{-1}]	Nr.	Symmetry	Wavenumber [cm^{-1}]	IR intensity [km mol^{-1}]
1	E	21.2	0	1	E	20.4	0
2	E	21.2	0	2	E	20.4	0
3	T_2	21.8	2	3	T_2	21.6	1
4	T_2	21.8	2	4	T_2	21.6	1
5	T_2	21.8	2	5	T_2	21.6	1
6	A_1	152.9	0	6	A_1	148.2	0
7	T_2	168.8	5	7	T_2	163.6	5
8	T_2	168.8	5	8	T_2	163.6	5
9	T_2	168.8	5	9	T_2	163.6	5
10	T_1	601.9	0	10	T_1	631.2	0
11	T_1	601.9	0	11	T_1	631.2	0
12	T_1	601.9	0	12	T_1	631.2	0
13	T_2	645.9	179	13	T_2	673.8	203
14	T_2	645.9	179	14	T_2	673.8	203
15	T_2	645.9	179	15	T_2	673.8	203
16	E	672.2	0	16	E	693.9	0
17	E	672.2	0	17	E	693.9	0
18	T_2	3545.5	1897	18	T_2	3693.7	1667
19	T_2	3545.5	1897	19	T_2	3693.7	1667
20	T_2	3545.5	1897	20	T_2	3693.7	1667
21	A_1	3615.8	0	21	A_1	3754.5	0

Table S 46. $[I(HF)_4]^-$.

B3LYP(D3BJ)/def2-TZVPP				SCS-MP2/def2-TZVPP			
Nr.	Symmetry	Wavenumber [cm^{-1}]	IR intensity [km mol^{-1}]	Nr.	Symmetry	Wavenumber [cm^{-1}]	IR intensity [km mol^{-1}]
1	T_2	18.1	2	1	E	10.6	0
2	T_2	18.1	2	2	E	10.6	0
3	T_2	18.1	2	3	T_2	13.0	2
4	E	18.6	0	4	T_2	13.0	2
5	E	18.6	0	5	T_2	13.0	2
6	A_1	135.9	0	6	A_1	129.2	0
7	T_2	145.1	2	7	T_2	138.1	3
8	T_2	145.1	2	8	T_2	138.1	3
9	T_2	145.1	2	9	T_2	138.1	3
10	T_1	548.2	0	10	T_1	583.9	0
11	T_1	548.2	0	11	T_1	583.9	0
12	T_1	548.2	0	12	T_1	583.9	0
13	T_2	582.9	154	13	T_2	614.2	184
14	T_2	582.9	154	14	T_2	614.2	184
15	T_2	582.9	154	15	T_2	614.2	184
16	E	609.7	0	16	E	632.1	0
17	E	609.7	0	17	E	632.1	0
18	T_2	3590.5	1954	18	T_2	3757.2	1613
19	T_2	3590.5	1954	19	T_2	3757.2	1613
20	T_2	3590.5	1954	20	T_2	3757.2	1613
21	A_1	3640.2	0	21	A_1	3798.0	0

Table S 47. Vibrational frequencies for $[F(HF)_4]^-$ and $[Cl(HF)_4]^-$ calculated on SCS-MP2(COSMO)/def2-TZVPP.

$[F(HF)_4]^-$				$[Cl(HF)_4]^-$			
Nr.	Symmetry	Wavenumber [cm^{-1}]	IR intensity [km mol^{-1}]	Nr.	Symmetry	Wavenumber [cm^{-1}]	IR intensity [km mol^{-1}]
1	E	34.2	0	1	T_2	19.6	1
2	E	34.2	0	2	T_2	19.6	1
3	T_2	49.6	3	3	T_2	19.6	1
4	T_2	49.6	3	4	E	20.1	0
5	T_2	49.6	3	5	E	20.1	0
6	A_1	252.3	0	6	A_1	169.4	0
7	T_2	300.3	42	7	T_2	202.1	19
8	T_2	300.3	42	8	T_2	202.1	19
9	T_2	300.3	42	9	T_2	202.1	19
10	T_1	806.7	0	10	T_1	585.2	0
11	T_1	806.7	0	11	T_1	585.2	0
12	T_1	806.7	0	12	T_1	585.2	0
13	T_2	897.9	490	13	T_2	609.5	389
14	T_2	897.9	490	14	T_2	609.5	389
15	T_2	897.9	490	15	T_2	609.5	389
16	E	911.7	0	16	E	631.6	0
17	E	911.7	0	17	E	631.6	0
18	T_2	3312.5	2257	18	T_2	3595.0	2261
19	T_2	3312.5	2257	19	T_2	3595.0	2261
20	T_2	3312.5	2257	20	T_2	3595.0	2261
21	A_1	3573.4	0	21	A_1	3683.7	0

Table S 48. Vibrational frequencies for $[Br(HF)_4]^-$ and $[I(HF)_4]^-$ calculated on SCS-MP2(COSMO)/def2-TZVPP.

$[Br(HF)_4]^-$				$[I(HF)_4]^-$			
Nr.	Symmetry	Wavenumber [cm^{-1}]	IR intensity [km mol^{-1}]	Nr.	Symmetry	Wavenumber [cm^{-1}]	IR intensity [km mol^{-1}]
1	*E*	16.7	0	1	*E*	5.9	0
2	*E*	16.7	0	2	*E*	5.9	0
3	T_2	21.9	2	3	T_2	16.7	2
4	T_2	21.9	2	4	T_2	16.7	2
5	T_2	21.9	2	5	T_2	16.7	2
6	A_1	151.0	0	6	A_1	130.5	0
7	T_2	164.8	10	7	T_2	139.2	6
8	T_2	164.8	10	8	T_2	139.2	6
9	T_2	164.8	10	9	T_2	139.2	6
10	T_1	526.5	0	10	T_2	457.9	356
11	T_1	526.5	0	11	T_2	457.9	356
12	T_1	526.5	0	12	T_2	457.9	356
13	T_2	536.0	363	13	T_1	458.5	0
14	T_2	536.0	363	14	T_1	458.5	0
15	T_2	536.0	363	15	T_1	458.5	0
16	*E*	561.6	0	16	*E*	483.0	0
17	*E*	561.6	0	17	*E*	483.0	0
18	T_2	3647.7	2354	18	T_2	3714.2	2336
19	T_2	3647.7	2354	19	T_2	3714.2	2336
20	T_2	3647.7	2354	20	T_2	3714.2	2336
21	A_1	3712.3	0	21	A_1	3753.4	0

Table S 49. $[Cl(HF)_2(HCl)]^-$.

B3LYP(D3BJ)/def2-TZVPP				SCS-MP2/def2-TZVPP			
Nr.	Symmetry	Wavenumber [cm^{-1}]	IR intensity [km mol^{-1}]	Nr.	Symmetry	Wavenumber [cm^{-1}]	IR intensity [km mol^{-1}]
1	*A′*	23.0	0	1	*A′*	15.0	0
2	*A″*	23.3	0	2	*A″*	17.3	0
3	*A′*	26.1	0	3	*A′*	20.3	0
4	*A′*	174.6	29	4	*A′*	159.0	26
5	*A′*	209.8	27	5	*A′*	201.7	27
6	*A″*	227.0	18	6	*A″*	221.3	21
7	*A′*	631.9	11	7	*A′*	632.3	16
8	*A″*	635.6	5	8	*A″*	636.1	10
9	*A″*	711.6	43	9	*A″*	750.5	46
10	*A′*	742.2	155	10	*A′*	772.8	179
11	*A″*	748.6	11	11	*A″*	775.4	9
12	*A′*	772.6	93	12	*A′*	801.5	110
13	*A′*	2082.1	2970	13	*A′*	2370.8	2480
14	*A″*	3379.2	2162	14	*A″*	3524.8	2051
15	*A′*	3437.2	1031	15	*A′*	3577.7	904

Table S 50. $[Br(HF)_2(HBr)]^-$.

B3LYP(D3BJ)/def2-TZVPP				SCS-MP2/def2-TZVPP			
Nr.	Symmetry	Wavenumber [cm^{-1}]	IR intensity [km mol^{-1}]	Nr.	Symmetry	Wavenumber [cm^{-1}]	IR intensity [km mol^{-1}]
1	*A′*	18.2	1	1	*A′*	–19.1	0
2	*A″*	18.4	0	2	*A″*	–15.4	0
3	*A′*	21.8	1	3	*A′*	20.9	1
4	*A′*	115.2	28	4	*A′*	102.9	24
5	*A′*	167.8	7	5	*A′*	165.0	8
6	*A″*	175.2	6	6	*A″*	174.7	8
7	*A′*	574.0	3	7	*A′*	564.8	5
8	*A″*	577.2	0	8	*A″*	581.4	2
9	*A″*	639.3	36	9	*A″*	680.9	38
10	*A′*	666.1	113	10	*A′*	710.7	146
11	*A″*	676.1	6	11	*A″*	719.3	4
12	*A′*	692.3	76	12	*A′*	725.7	83
13	*A′*	1711.4	3728	13	*A′*	2007.8	3015
14	*A″*	3481.7	1977	14	*A″*	3616.9	1902
15	*A′*	3520.1	1109	15	*A′*	3651.9	953

Table S 51. $[I(HF)_2(HI)]^-$.

B3LYP(D3BJ)/def2-TZVPP				SCS-MP2/def2-TZVPP			
Nr.	Symmetry	Wavenumber [cm^{-1}]	IR intensity [km mol^{-1}]	Nr.	Symmetry	Wavenumber [cm^{-1}]	IR intensity [km mol^{-1}]
1	*A″*	15.2	0	1	*A′*	8.7	1
2	*A′*	15.4	2	2	*A″*	15.6	0
3	*A′*	18.9	1	3	*A′*	15.7	2
4	*A′*	86.4	26	4	*A′*	70.7	21
5	*A′*	142.8	4	5	*A′*	137.0	4
6	*A″*	147.0	3	6	*A″*	141.8	4
7	*A′*	495.0	0	7	*A′*	483.9	0
8	*A″*	499.5	1	8	*A″*	489.7	0
9	*A″*	567.5	31	9	*A″*	616.5	37
10	*A′*	591.0	78	10	*A′*	627.1	111
11	*A″*	600.1	3	11	*A″*	631.4	3
12	*A′*	614.1	68	12	*A′*	652.4	84
13	*A′*	1395.2	4818	13	*A′*	1775.0	3594
14	*A″*	3561.5	1902	14	*A″*	3710.3	1694
15	*A′*	3585.7	1233	15	*A′*	3731.7	1015

Table S 52. $[Cl(HF)_2(HCl)_2]^-$.

B3LYP(D3BJ)/def2-TZVPP				SCS-MP2/def2-TZVPP			
Nr.	Symmetry	Wavenumber [cm^{-1}]	IR intensity [km mol^{-1}]	Nr.	Symmetry	Wavenumber [cm^{-1}]	IR intensity [km mol^{-1}]
1	A_1	14.8	0	1	A_1	15.4	0
2	A_2	18.4	0	2	A_2	19.8	0
3	B_2	20.5	0	3	B_2	22.3	0
4	B_1	21.7	1	4	A_1	23.6	0
5	A_1	24.0	1	5	B_1	23.8	1
6	A_1	138.1	6	6	A_1	129.6	6
7	B_1	159.5	38	7	B_1	151.7	33
8	A_1	195.4	17	8	A_1	188.8	16
9	B_2	210.5	12	9	B_2	203.9	14
10	B_1	533.5	2	10	B_1	546.6	5
11	A_2	547.8	0	11	A_2	569.7	0
12	B_2	554.2	16	12	B_2	576.7	24
13	A_1	576.5	25	13	A_1	580.0	28
14	B_2	660.9	63	14	B_2	695.1	70
15	B_1	686.6	147	15	A_2	718.8	0
16	A_2	688.9	0	16	B_1	721.8	174
17	A_1	710.6	95	17	A_1	742.0	112
18	B_1	2314.4	2928	18	B_1	2557.7	2332
19	A_1	2399.6	1365	19	A_1	2619.8	1092
20	B_2	3508.4	1837	20	B_2	3637.6	1662
21	A_1	3554.8	978	21	A_1	3679.5	892

Table S 53. $[Br(HF)_2(HBr)_2]^-$.

B3LYP(D3BJ)/def2-TZVPP				SCS-MP2/def2-TZVPP			
Nr.	Symmetry	Wavenumber [cm^{-1}]	IR intensity [km mol^{-1}]	Nr.	Symmetry	Wavenumber [cm^{-1}]	IR intensity [km mol^{-1}]
1	A_1	8.7	0	1	A_1	9.1	0
2	B_2	14.9	0	2	B_2	15.1	0
3	A_2	15.3	0	3	A_2	16.7	0
4	B_1	16.7	2	4	B_1	18.8	2
5	A_1	20.2	2	5	A_1	20.2	1
6	A_1	92.8	7	6	A_1	85.8	7
7	B_1	99.1	26	7	B_1	94.5	22
8	A_1	156.7	6	8	A_1	155.7	6
9	B_2	165.2	5	9	B_2	163.3	6
10	B_1	480.8	0	10	B_1	487.2	1
11	A_2	495.6	0	11	A_2	499.1	0
12	B_2	497.1	2	12	B_2	501.4	7
13	A_1	515.9	6	13	A_1	512.0	8
14	B_2	598.1	45	14	B_2	638.5	48
15	B_1	615.5	97	15	B_1	669.7	127
16	A_2	624.5	0	16	A_2	671.6	0
17	A_1	640.2	75	17	A_1	679.3	93
18	B_1	1966.6	3701	18	B_1	2213.9	2761
19	A_1	2061.7	1554	19	A_1	2278.4	1162
20	B_2	3578.0	1752	20	B_2	3700.4	1571
21	A_1	3609.8	1058	21	A_1	3729.0	951

Table S 54. $[I(HF)_2(HI)_2]^-$.

B3LYP(D3BJ)/def2-TZVPP				SCS-MP2/def2-TZVPP			
Nr.	Symmetry	Wavenumber [cm^{-1}]	IR intensity [km mol^{-1}]	Nr.	Symmetry	Wavenumber [cm^{-1}]	IR intensity [km mol^{-1}]
1	A_1	4.6	0	1	A_1	6.6	1
2	B_2	11.5	0	2	B_2	8.0	0
3	A_2	13.0	0	3	A_1	11.3	1
4	B_1	13.8	3	4	A_2	17.9	0
5	A_1	17.7	2	5	B_1	18.6	2
6	A_1	68.6	6	6	A_1	59.5	6
7	B_1	71.4	26	7	B_1	64.1	19
8	A_1	135.3	4	8	A_1	132.0	3
9	B_2	140.8	2	9	B_2	137.1	3
10	B_1	410.4	2	10	B_1	416.6	0
11	B_2	424.7	1	11	B_2	430.2	0
12	A_2	426.2	0	12	A_2	430.7	0
13	A_1	436.8	0	13	A_1	433.7	0
14	B_2	535.9	34	14	B_2	587.4	39
15	B_1	549.4	57	15	B_1	596.9	98
16	A_2	559.9	0	16	A_2	601.0	0
17	A_1	574.1	63	17	A_1	619.3	83
18	B_1	1646.8	4998	18	B_1	1965.4	3226
19	A_1	1746.5	1817	19	A_1	2022.3	1243
20	B_2	3628.1	1752	20	B_2	3763.5	1513
21	A_1	3648.9	1210	21	A_1	3781.8	986

Table S 55. HCN.

B3LYP(D3BJ)/def2-TZVPP				SCS-MP2/def2-TZVPP			
Nr.	Symmetry	Wavenumber [cm^{-1}]	IR intensity [km mol^{-1}]	Nr.	Symmetry	Wavenumber [cm^{-1}]	IR intensity [km mol^{-1}]
1	Π	767.8	37	1	Π	731.9	37
2	Π	767.8	37	2	Π	731.9	37
3	Σ^+	2201.1	1	3	Σ^+	2066.2	0
4	Σ^+	3443.5	65	4	Σ^+	3467.4	72

Table S 56. $[Br(HCN)]^-$.

B3LYP(D3BJ)/def2-TZVPP				SCS-MP2/def2-TZVPP			
Nr.	Symmetry	Wavenumber [cm^{-1}]	IR intensity [km mol^{-1}]	Nr.	Symmetry	Wavenumber [cm^{-1}]	IR intensity [km mol^{-1}]
1	Σ^+	159.1	16	1	Σ^+	158.7	16
2	Π	166.1	20	2	Π	194.5	20
3	Π	166.1	20	3	Π	194.5	20
4	Π	982.5	16	4	Π	1010.6	17
5	Π	982.5	16	5	Π	1010.6	17
6	Σ^+	2092.5	555	6	Σ^+	2013.6	223
7	Σ^+	2834.1	1339	7	Σ^+	2945.9	1398

Table S 57. $[ClO_4]^-$.

B3LYP(D3BJ)/def2-TZVPP				SCS-MP2/def2-TZVPP			
Nr.	Symmetry	Wavenumber [cm^{-1}]	IR intensity [km mol^{-1}]	Nr.	Symmetry	Wavenumber [cm^{-1}]	IR intensity [km mol^{-1}]
1	E	449.5	0	1	E	470.2	0
2	E	449.5	0	2	E	470.2	0
3	T_2	617.4	23	3	T_2	647.3	29
4	T_2	617.4	23	4	T_2	647.3	29
5	T_2	617.4	23	5	T_2	647.3	29
6	A_1	918.4	0	6	A_1	1001.7	0
7	T_2	1103.0	384	7	T_2	1175.3	429
8	T_2	1103.0	384	8	T_2	1175.3	429
9	T_2	1103.0	384	9	T_2	1175.3	429

Table S 58. $[ClO_4(HF)_2]^-$.

B3LYP(D3BJ)/def2-TZVPP				SCS-MP2/def2-TZVPP			
Nr.	Symmetry	Wavenumber [cm^{-1}]	IR intensity [km mol^{-1}]	Nr.	Symmetry	Wavenumber [cm^{-1}]	IR intensity [km mol^{-1}]
1	B_1	10.1	1	1	B_1	12.0	1
2	A_2	15.0	0	2	A_2	20.5	0
3	A_1	42.3	2	3	A_1	39.2	1
4	B_2	79.1	0	4	B_2	73.1	0
5	A_1	178.0	0	5	A_1	168.6	0
6	B_2	258.9	50	6	B_2	242.9	48
7	A_2	439.5	0	7	A_2	463.8	0
8	A_1	470.6	3	8	A_1	489.0	3
9	B_1	605.6	14	9	B_1	637.5	15
10	B_2	607.5	24	10	B_2	640.0	33
11	A_1	636.2	6	11	A_1	663.1	6
12	A_2	767.1	0	12	A_2	772.3	0
13	B_1	770.5	190	13	B_1	775.7	205
14	B_2	847.5	10	14	B_2	842.8	5
15	A_1	847.7	138	15	A_1	843.9	164
16	A_1	919.3	19	16	A_1	1001.6	18
17	B_2	1042.2	659	17	B_2	1116.0	710
18	A_1	1113.9	359	18	A_1	1185.4	394
19	B_1	1166.8	363	19	B_1	1237.2	406
20	B_2	3451.8	3239	20	B_2	3615.2	2882
21	A_1	3488.4	33	21	A_1	3646.4	27

Table S 59. $[I(HBr)_2]^-$.

B3LYP(D3BJ)/def2-TZVPP				SCS-MP2/def2-TZVPP			
Nr.	Symmetry	Wavenumber [cm^{-1}]	IR intensity [km mol^{-1}]	Nr.	Symmetry	Wavenumber [cm^{-1}]	IR intensity [km mol^{-1}]
1	A_1	10.3	0	1	A_1	10.1	0
2	A_1	98.2	9	2	A_1	85.9	9
3	B_1	98.8	39	3	B_1	88.2	34
4	B_1	526.1	3	4	B_1	525.1	0
5	B_2	533.6	4	5	B_2	531.2	7
6	A_2	542.7	0	6	A_2	537.8	0
7	A_1	580.1	1	7	A_1	558.9	2
8	B_1	1610.6	6297	8	B_1	1939.9	5161
9	A_1	1749.2	2345	9	A_1	2036.4	1831

g) Raman Spectra of Samples Cooled to –196 °C

Method for low temperature Raman spectroscopy of single crystals:

1) Transfer of the sample from the reaction flask into a cool nitrogen stream where a crystal can be selected (Figure S 23).
2) Transfer of the selected crystal onto a Teflon plate which is cooled by a copper block which is cooled by liquid nitrogen (Figure S 24, Figure S 25). By evaporation of the nitrogen a layer of cool gaseous nitrogen protects the sample from moisture.
3) Measuring of the Raman spectra using a Raman microscope (Figure S 26).

As often rather big needle shaped crystals were obtained often a crystal was split with a scalpel and one part was used for XRD while the other part was used for Raman spectroscopy.

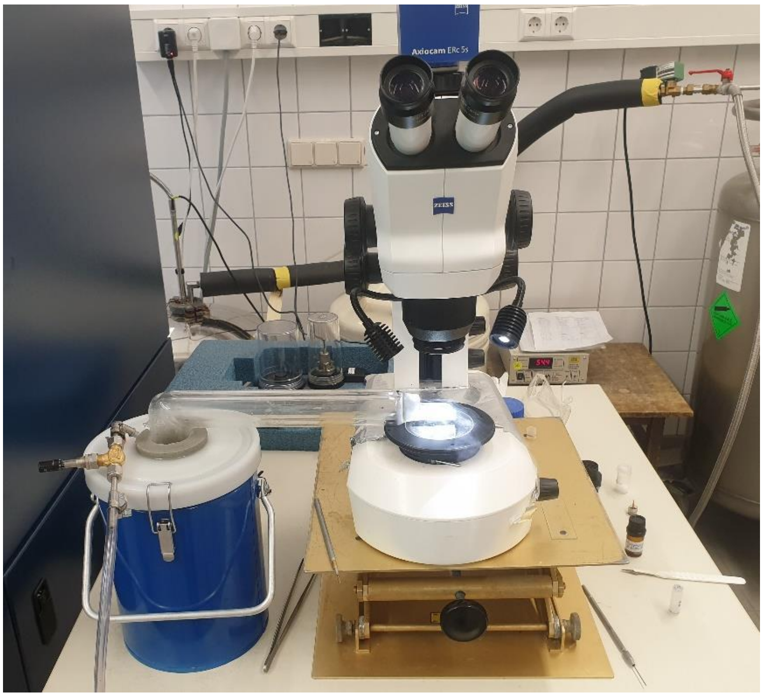

Figure S 23. Setting for preparing a single crystal for x-ray diffraction or low temperature Raman spectroscopy.

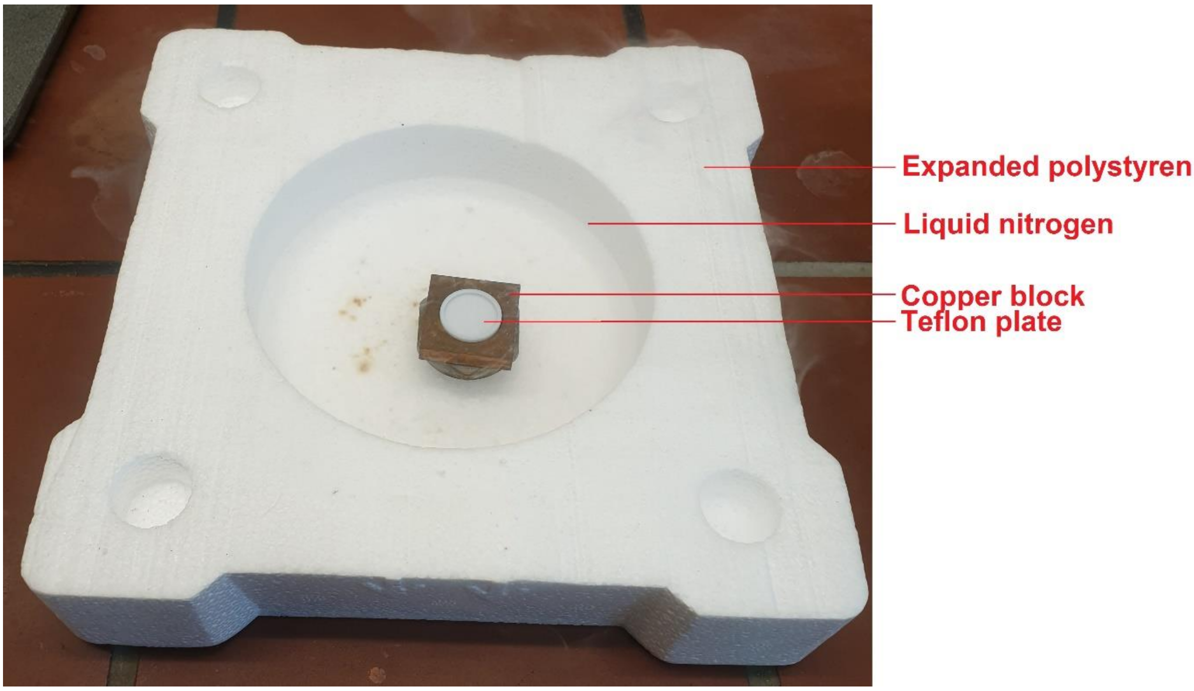

Figure S 24. Setting for cooling a sample to –196 °C for low temperature Raman spectroscopy.

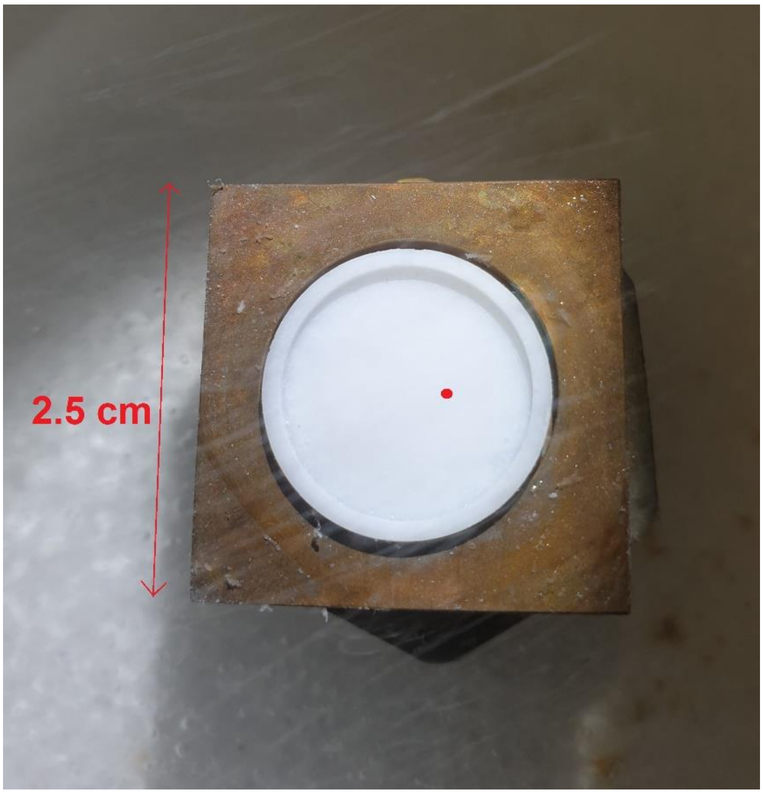

Figure S 25. Prepared crystal (red dot) on the Teflon plate for low temperature Raman spectroscopy.

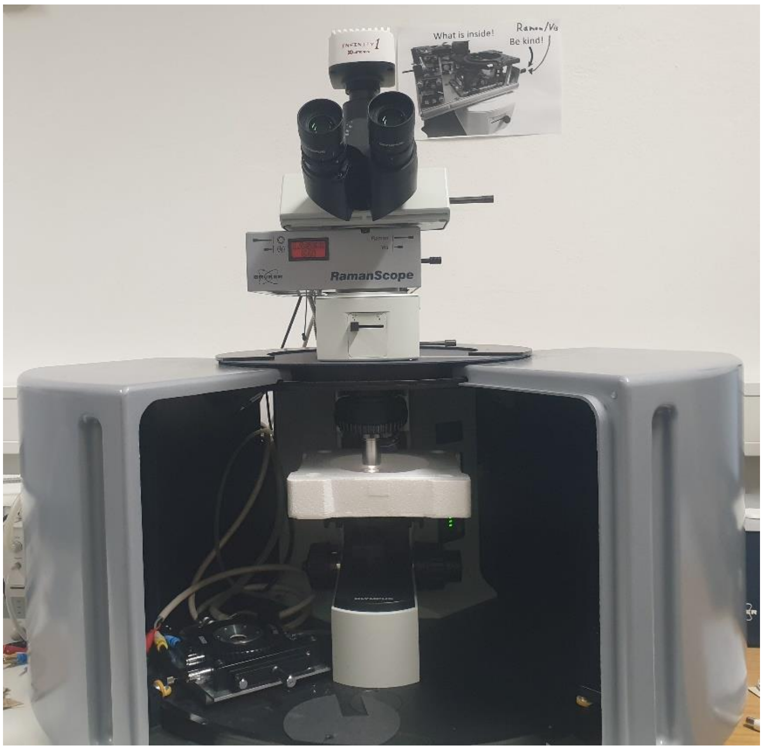

Figure S 26. Assembly in the RamanScope.
